# Implementation determinants of physical activity interventions in primary health care settings using the TICD framework: a systematic review

**DOI:** 10.1186/s12913-023-09881-y

**Published:** 2023-10-11

**Authors:** Catarina Santos Silva, Cristina Godinho, Jorge Encantado, Bruno Rodrigues, Eliana V. Carraça, Pedro J. Teixeira, Marlene Nunes Silva

**Affiliations:** 1 CIPER, Faculdade de Motricidade Humana, Universidade de Lisboa, Lisboa, Portugal; 2 Programa Nacional Para a Promoção da Atividade Física, Direção-Geral da Saúde, Lisboa, Portugal; 3 NOVA National School of Public Health, Public Health Research Centre, Comprehensive Health Research Center, CHRC, NOVA University Lisbon, Lisboa, Portugal; 4 CIAFEL, Faculdade de Desporto da Universidade Do Porto, Porto, Portugal; 5 CIDEFES, Centro de Investigação em Desporto, Educação Física, Exercício e Saúde, Universidade Lusófona, Lisboa, Portugal

**Keywords:** Determinants of implementation, Barriers, Facilitators, Physical activity interventions, Primary care

## Abstract

**Background:**

Translation into practice of effective physical activity interventions in primary care is difficult, due to a complex interaction of implementation determinants. We aimed to identify implementation barriers and facilitators of four primary care interventions: physical activity assessment, counselling, prescription, and referral.

**Methods:**

A systematic review of qualitative, quantitative and mixed-methods studies published since 2016 was conducted. The “Tailored Implementation for Chronic Diseases” (TICD) framework was adapted to extract and synthesize barriers and facilitators.

**Results:**

Sixty-two studies met the inclusion criteria. Barriers (*n* = 56) and facilitators (*n* = 55) were identified across seven domains, related to characteristics of the intervention, individual factors of the implementers and receivers, organizational factors, and political and social determinants. The five most frequently reported determinants were: professionals’ knowledge and skills; intervention feasibility/compatibility with primary health care routine; interventions’ cost and financial incentives; tools and materials; and professionals’ cognitions and attitudes. “Social, political and legal factors” domain was the least reported. Physical activity counselling, prescription, and referral were influenced by determinants belonging to all the seven domains.

**Conclusion:**

The implementation of physical activity interventions in primary care is influenced by a broader range of determinants. Barriers and facilitators related with health professionals, intervention characteristics, and available resources were the most frequently reported. A deep understanding of the local context, with particularly emphasis on these determinants, should be considered when preparing an intervention implementation, in order to contribute for designing tailored implementation strategies and optimize the interventions’ effectiveness.

**Supplementary Information:**

The online version contains supplementary material available at 10.1186/s12913-023-09881-y.

## Background

The importance of maintaining regular physical activity (PA) is well established both for preventive care [1] and as a therapeutic adjuvant [2], in several chronic conditions. However, worldwide physical inactivity prevalence remains high [3–5].

The critical role of health systems in the promotion of PA as a way of tackling non-communicable diseases has been highlighted by the World Health Organization (WHO) during the last decade [6] with primary health care services gaining more relevance particularly since 2016 [7, 8]. More recently, the WHO Global Action Plan for PA Promotion 2018–2030 [9] has established the development of PA promotion systems within health care services – directed at patients and implemented by appropriately trained health professionals – as a priority action. A toolkit specifically designed to primary care [10] has since been created, encompassing strategies developed to support countries implementing and strengthening systems of patients’ PA assessment and counselling, as part of universal health care. Despite efforts made, only 40% of countries reported having a national protocol in this regard in 2021 [3].

Several types of primary care intervention models have been developed. They can be grouped in four major intervention types [10]: i. *PA screening/assessment*, which corresponds to a systematic application of an enquiry to identify patients’ levels of PA and sedentary behaviour [10, 11]; ii. *PA brief counselling/advice*, comprising a verbal encouragement and/or a verbal or written recommendation for PA, performed by a professional during routine care, also involving an approach to motivations, barriers, preferences, readiness, patient's health, and opportunities to perform PA [10, 12, 13]; iii. *exercise prescription*, comprising an initial assessment of the patients’ physical and functional fitness, body composition, past PA and clinical history, and goals/motivations, followed by a detailed selection and explanation of exercises according to the patients’ initial assessment, and also including a systematic monitoring and evaluation of effects [12]; and iv. *exercise referral scheme*, made by a primary care professional to a third-party service, which is responsible to prescribe a tailored PA/exercise program to the patient [10, 13–15]. These intervention types can be implemented individually or in combination.

Previous research evaluating these interventions has revealed clinically relevant increases in patients’ PA levels [16–20]. However, studies assessing interventions’ external validity, when implemented in real-world settings and integrated in primary health care assistance activities, are lacking, limiting the generalizability of such results [20]. The current research-to-practice evidence gap highlights the importance of addressing contextual determinants (barriers and facilitators) to generate evidence for implementation strategies, thus contributing for the translation of evidence-based interventions into healthcare practice [13, 21–23].

Key determinants of healthcare practice may be related to environmental (e.g., socio-political and legal factors) or organizational characteristics (e.g., decision-making processes, capacity for organizational change, and the existence or absence of resources and incentives), but also with characteristics of implementers, receivers, and/or the intervention itself. These determinants have been systematized through different checklists, frameworks, taxonomies, and classification systems [24–29]. Based on these, a comprehensive and integrated checklist of determinants was specifically developed for healthcare professional practice – the “Tailored Implementation for Chronic Diseases” (TICD) checklist [30], to optimize reflection and data collection on determinants of implementation. When introducing quality improvements or new interventions in healthcare, a proper investigation of implementation barriers and facilitators is critical to reveal the most relevant intervention- and context-specific ones, aiming at the development of tailored implementation strategies and more effective interventions [30].

There is a limited number of systematic reviews aimed at reporting implementation barriers and facilitators of PA interventions [31]. Some have focused in the primary health care system, but have not included PA-only interventions alone (considering weight management programs and lifestyle interventions, for instance), and were limited to analysing stakeholders’ views [32] or health professionals’ determinants and views [33–35], and/or considered a single PA intervention type [19, 35]. Thus, there is a need for systematic identification of whole-system implementation barriers and facilitators of the most common PA-specific promotion interventions implemented in primary care.

This systematic review aimed to identify implementation barriers and facilitators, according to the TICD framework, within the four described PA promotion interventions delivered in primary health care settings by health professionals to adult patients.

## Methods

This systematic review was reported in accordance with the Preferred Reporting Items for Systematic reviews and Meta-Analyses (PRISMA) 2020 statement [36] (see Additional file 1).

### Eligibility criteria

We included peer-reviewed studies published since January 2016, the publication year of both the “Physical activity strategy for the WHO European Region 2016–2025” [7] and the guide “Integrating diet, physical activity and weight management services into primary care” [8]. Although there are studies on this topic published before this year, constant changes in health care systems, scientific knowledge, and population health pattern might make older studies not representative of today’s reality. Furthermore, 2016 marked a stronger and more focused WHO’s recommendation of PA promotion interventions in primary health care. Therefore, only studies published since 2016 were considered. We considered studies with primary care health professionals, patients (≥ 18 years), and stakeholders involved in one of the four types of PA promotion and/or sedentary time reduction interventions (i.e., PA assessment, counselling, prescription and/or referral), delivered in primary health care settings, at least in part, face-to-face. Included studies should formally assess interventions’ implementation barriers and facilitators. Several types of study design were included (i.e., qualitative, quantitative or mixed-methods).

Studies including rehabilitation patients, or patients with contraindications to perform PA autonomously, those testing interventions not specifically targeting PA promotion alone (e.g., lifestyle interventions, weight management interventions, etc.) or digital-only interventions, study protocols, literature reviews, opinion articles, conference books or papers, non-peer reviewed scientific literature (e.g., books, book chapters), and non-English or Portuguese written literature were excluded.

### Information sources

A systematic literature search for titles and abstracts was conducted in five electronic databases: Web of Science, Scopus, PsycInfo, PubMed, and Medline. Databases were last searched in July 12^th^, 2023.

### Search strategy

The search strategy comprised a combination of terms from four different categories: behaviours of interest, interventions of interest, implementation context, and review’s main outcomes (i.e., implementation determinants). The full search stem can be found in Additional file 2.

### Selection and data collection processes

Two reviewers (CSS and JE) independently screened titles and abstracts and three reviewers (CSS, JE, and BR) independently analysed full text articles against eligibility criteria. A consistency check between the authors was performed in 15% of randomly selected titles and abstracts and in 20% of randomly selected full-texts to obtain inter-reviewer agreement (Cohen’s kappa and Fleiss’ kappa, respectively). Authors were blind to each other’s decisions and, given that good to excellent agreement was found in their assessments (Cohen *k* = 1; Fleiss’ *k* = 0.615), they independently screened the other 85% of titles and abstracts and 80% of full text articles. Disagreements between individual decisions were discussed to reach consensus. CADIMA® online software was used to record decisions on title and abstract screening and full text analysis. When full text articles were unavailable, authors were contacted and readily made their work available in all cases. Three reviewers (CSS, JE, and BR) independently extracted data. An excel spreadsheet was used to record extracted data. TICD framework categories [30] were used to guide data extraction.

### Data items

Extracted data comprised the following outcome items of significance to the review objectives: guideline factors; individual health professional factors; patient factors; professional interactions; incentives and resources; capacity for organizational change; social, political, and legal factors; and any other factor assessed as a barrier and/or facilitator of implementation of the interventions of interest. Relevant statistical data on the outcomes of interest was also extracted, when applicable, as an indicator of its relevance. Other study information was also extracted: author; year; country of implementation; type of study; methodology; trial (if applicable); intervention; outcome; and participants’ characteristics (number of participants; health professional or stakeholder category or if the sample consisted of patients; mean age; sex distribution; patients’ chronic diseases, if applicable).

### Study quality assessment

Two authors (CSS and JE) independently performed a critical appraisal of all articles included in the review. A consistency check between the two authors was performed in 15% of randomly selected studies, having obtained a good inter-reviewer agreement (Cohen’s *k* = 0.653). Joanna Brigs Institute (JBI) critical appraisal tools [37] were used to assess studies’ quality. For studies using a mixed-methods methodology, the Mixed-Methods Appraisal Tool (MMAT) [38] was applied, as there is no specific JBI tool for mixed-methods studies. The critical appraisal assessment is presented for each study against each checklist item, in table format [39].

### Synthesis methods

As this systematic review includes very different studies and its output is qualitative, a narrative synthesis was performed. First, a preliminary synthesis was made using a thematic analysis approach [40], based on the TICD framework, and studies’ results were presented in tabular form, structured into the framework’s main themes/domains, barriers *vs*. facilitators, and type of PA promotion intervention. Then, a frequency table of the studies mentioning each kind of implementation barrier and facilitator was made. Last, the studies and their results were presented and relationships in the data were explored, to better interpret the facilitators and barriers of each type of PA promotion intervention. This allowed to understand the different implementation determinants in an articulated, integrated, and systematic way.

### Certainty assessment of the systematic review

The Supporting the Use of Research Evidence (SURE) checklist was used, to evaluate the identification, selection and appraisal of studies (5 criteria), how findings were analysed (5 criteria), and to reflect on other considerations (one criterion) [41].

## Results

### Study selection

**Fig. 1:**
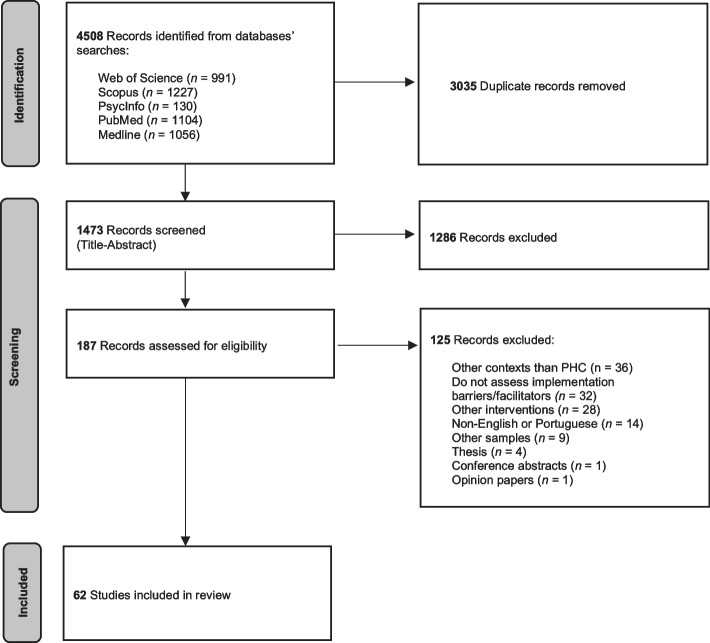
PRISMA flowchart

The search strategy identified a total of 4508 records (see Fig. 1). After duplicates removal and title and abstract screening, the full-text of 187 records were assessed for eligibility. After exclusion of 125 records for not meeting inclusion criteria, a total of 62 articles were included in this review [42–103].

### Study characteristics

From the 62 articles included, 48.4% (*n* = 30) employed a qualitative design [43, 46, 48, 49, 54, 55, 58, 60, 62, 65, 67, 68, 71, 76–80, 83, 84, 87–89, 92–97, 99], 37.1% (*n* = 23) a quantitative design [42, 44, 45, 52, 53, 57, 61, 63, 64, 66, 69, 72–75, 81, 82, 85, 91, 98, 100–102], and 14.5% (*n* = 9) a mixed-methods study design [47, 50, 51, 56, 59, 70, 86, 90, 103]. The majority of the studies (87.1%) were conducted in high income countries (United Kingdom, *n* = 15; Canada, *n* = 9; USA, *n* = 7; other countries, *n* = 23), while only 12.9% were conducted in upper middle income countries (Brazil, *n* = 4; Thailand, *n* = 2; Jordan, *n* = 1; Turkey, *n* = 1), according to the categorization of the World Bank [104]. Study characteristics are outlined in Table 1.

**Table 1:** Study characteristics

Refe-rence	Author & Year	Country	Study design	Data collection sources/method(s)	PA intervention type	Sample characteristics^a^	Quality (“yes” items/total nr. of items)
[42]	Kyei-Frimpong et al., 2021	USA	Quasi-experimental	retrospective chart reviews and practitioners’ surveys	PA prescription	Practitioners: *n* = 12 (11 medical doctors, 1 physician assistant) Patients: *n* = 125 patients; ≥ 18 years; without cognitive or physical impairment	4/9
[43]	Syrjälä et al., 2021	Sweden	Qualitative	semi-structured interviews	PA counselling	15 patients; average age = 55 years; 46,7% females; type 2 diabetes	7/10
[44]	Charles et al., 2022	France	Analytical cross-sectional	survey/self-administered questionnaire	PA prescription	283 GPs; average age = 46,6 years	5/8
[45]	Souza-Neto et al., 2021	Brazil	Analytical cross-sectional	self-administered questionnaire	PA counselling	591 health professionals (general practitioners, nurses, nurse assistants, community health workers); average age = 43,2 years; 78,6% females	6/8
[46]	Huntington et al., 2020	Canada	Qualitative	semi-structured interviews	PA counselling	20 registered dietitians; age categories: 25–34 years, 65%; 35–44 years, 15%; 45–54 years, 5%; 55–64 years, 15%; 95% females	8/10
[47]	Buckley et al., 2020	UK	Mixed-methods	online survey and semi-structured interviews	PA counselling and referral	Survey: 56 GPs; average age = 45,5 years; 50% female Interviews: 7 GPs	3/7
[48]	Sissons et al., 2020	UK	Qualitative	semi-structured interviews	PA counselling	15 primary care professionals (8 GPs—average age = 38.3 years, 50% females; 7 community pharmacists—average age = 42.4 years, 29% females)	6/10
[49]	Carstairs et al., 2020	UK	Qualitative	semi-structured interviews	PA referral scheme	14 professionals (64,3% GPs; 35,7% practice nurses): 57,1% age category 45–54 years; 50% females 14 patients: 35,7% age category 45–54 years and 28,6% age category 65 years or above; 57,1% females	8/10
[50]	Alghafri et al., 2020	Oman	Mixed-methods	participants exit survey—interviewer-led questionnaire; health professionals exit survey—self-reported questionnaire	PA prescription	82 patients (Intervention Group) (54% females; type 2 diabetes); 16 health professionals (8 doctors, 8 nurses, 4 dietitians; 88% females)	6/7
[51]	Parjanen, 2021	Finland	Mixed-methods	questionnaire, facilitated workshops on questionnaire themes, focus group interviews	PA counselling and referral	Questionnaire: 39 Health care professionals (physicians, nurses, public health nurses and physiotherapists) Workshops: nurses and physiotherapists (*n* not indicated) Focus groups: physical activity counsellors (*n* = 8) and potential clients (*n* = 13)	7/7
[52]	Yonis et al., 2020	Jordan	Analytical cross-sectional	self-reported survey	PA counselling	218 Primary care physicians: average age (SD) = 33,7 years (9,87); 67% males	6/8
[53]	Agadayi et al., 2019	Turkey	Prevalence	questionnaire applied by interview	PA prescription	145 research assistant and specialist physicians of Family Medicine; age categories: 61,4% 25–29 years, 27,6% 30–39 years, 11% 40 years or above; 71,7% females	2/9
[54]	Joelsson et al., 2020	Sweden	Qualitative	semi-structured interviews	PA prescription	20 patients; average age = 58 years (25–73); 45% females; physically inactive with one or more metabolic syndrome components	8/10
[55]	Christiansen et al., 2020	Canada	Qualitative	semi-structured interviews	PA prescription	11 primary care physicians	7/10
[56]	Bird et al., 2019	UK	Mixed-methods	follow-up questionnaires, qualitative telephone semi-structured interviews, and programme-related documentation	PA referral scheme	Questionnaires: 326 patients—age distribution (1,8% 18–34 years; 4,3% 35–50 years; 38,3% 51–69 years; 55,2% 70 years or above); 62,6% females; inactive adults diagnosed with type 2 diabetes, pre-diabetes, hypertension and/or obesity and overweight Semi-structured telephone interviews: Program stakeholders (1 programme manager, 1 practising General Practitioner—and member of the project steering group -, 3 the exercise specialists responsible for delivery) and 10 CLICK into Activity participants	7/7
[57]	Alahmed & Lobelo, 2019	Saudi Arabia	Analytical cross sectional	self-reported questionnaire	PA counselling	147 PHC physicians; median age = 31 years; 70.8% female	5/8
[58]	Westland et al., 2018	Netherlands	Qualitative	semi-structured interviews	PA counselling	14 nurses; average age = 48.9 years (range 24–63); 100% female	8/10
[59]	Harris et al., 2018	UK	Mixed-methods	Accelerometry, economic evaluation, focus groups, individual interviews	PA counselling	RCT: 1023 patients, without PA contraindications, 64% female, aged 45–75 years Process evaluation: 7 nurses (demographic data not available) 43 patients, 20 participants in the 45- to 59-year-old age group, 29 participants being female	7/7
[60]	Leenaars et al., 2018	Netherlands	Qualitative	interviews	PA referral scheme	15 Care sport connectors; average age = 38 years (min 27 years, max 57 years); 67% female	6/10
[61]	Baillot et al., 2018	Canada (Quebec)	Analytical cross sectional	questionnaire; medical chart audit	PA assessment; PA counselling	439 patients (median age: 59.1, Inter quartile range [IQR]: 49.3 to 67.2; 66.1% women); 40 family physicians (median age: 49.9 years, IQR: 43.9 to 57.3 years, 62.5% women); 24 nurses (median age: 33.5 years, IQR: 26.7 to 41.4 years, 91.7% women)	4/8
[62]	Gustavsson et al., 2018	Sweden	Qualitative	semi-structured interviews	PA prescription	18 stakeholders in SPAP in two regional healthcare organizations in Sweden (eighteen stakeholders were interviewed: five stakeholders at management level from each region plus five and three health professionals from each region respectively)	7/10
[63]	Al-Ghamdi et al., 2018	Saudi Arabia	Prevalence	self-report questionnaire	PA counselling	803 primary health care providers (309 physicians, 424 nurses, 26 nurse assistants, 31 dietician and 13 health educators); average age = 33.06 ± 8.45 years; 60% female	6/9
[64]	Fowles et al., 2018	Canada	Quasi-experimental	self-reflexion questionnaire	PA counselling and prescription	46 Physicians, age 49 (9), 50% female	5/9
[65]	Henderson et al., 2018	UK	Qualitative	semi-structured interviews	PA referral scheme	17 stakeholders (7 exercise professionals, 5 health professionals and 5 strategic managers)	10/10
[66]	Johnson et al., 2018	Australia	Analytical cross sectional	self-administered questionnaire, medical summary sheets	PA counselling	132 patients; 57 ± 13 years; 72% woman; 74% ≤ 1 chronic diseases and 22.7% ≥ 2 chronic diseases	6/8
[67]	Alghafri et al., 2017	Oman	Qualitative	focus group discussions	PA counselling	29 health professionals (17 doctors, 5 nurses, 4 dietitians, 3 health educators); 69% females	9/10
[68]	Bélanger et al., 2017	Canada	Qualitative	4 interviews during a 12-month period (0, 2, 4 and 12 months)	PA prescription	11 family physicians; 64% women; no prior experience in writing physical activity prescriptions but had a desire to implement it into their practice	7/10
[69]	Cottrell et al., 2017	UK	Prevalence	self-reported questionnaire survey	PA counselling	835 GPs; NR; 51% woman	8/9
[70]	Barrett et al., 2017	Republic of Ireland	Mixed-methods	modified Delphi approach	PA counselling and referral	41 physiotherapists; NR; 39% woman	5/7
[71]	Matthews et al., 2017	UK	Qualitative	Observation, interviews, documentation	PA counselling	8 stakeholders (1 HPEP programme tutor, 1 Generation Games programme manager, 1 OCDS Clinical Lead, 1 OCDS diabetes nurse, 4 practice nurses), 4 diabetes patients who were also Generation Games participants	7/10
[72]	O'Brian et al., 2017	Canada	Quasi-experimental	self-reflection questionnaire	PA counselling and prescription	113 physicians, 54 allied health professionals and 23 exercise professionals; 49 ± 11 years, 43 ± 12 years; 38 ± 10 years; 42.5% women, 94.4% women, 73.9% women	3/9
[73]	Leavitt, 2017	USA	Quasi-experimental	patient records/chart audit records	PA prescription	6 part-time volunteer primary care providers and 3 part-time nursing support staff form the patient care team	4/9
[74]	Hidalgo et al., 2016	Brazil	Prevalence	questionnaire	PA counselling	798 health professionals (82 physicians, 347 nurses, and 269 community health workers)—Age categories: 35,6% (20–29 years), 35,5% (30–39 years), 16,8% (40–49 years), 9,2% (50–59 years), 2,9% (> 60 years); 76,8% % female	4/9
[75]	Aittasalo et al., 2016	Finland	Quasi-experimental	Patient and professionals’ questionnaires, professionals’ record sheets on patient visits, telephone interviews	PA counselling	75, physician, nurse, physiotherapist; Age 54.1 (17.8) years; 65% female	4/9
[76]	Dutton et al., 2016	Australia	Qualitative	2 interviews during a 12-month period	PA assessment and counselling	9 family physicians, 10 practice nurses,	7/10
[77]	Leenaars et al., 2016	Netherlands	Qualitative	Focus group	PA counselling and referral	10 sport professionals; primary health care 20; welfare 7	7/10
[78]	Avery et al., 2016	UK	Qualitative	Semi-structured interviews	PA counselling	professionals (*n* = 9) and patients (*n* = 14)	6/10
[79]	Clark et al., 2021	Canada	Qualitative	interviews and focus groups	PA assessment	9 Physicians/Nurse Practitioners; 7 Nurses; 11 Other healthcare professionals (physical therapists, nurses, or occupational therapist); 3 Other stakeholders; 4 Patients—average age (SD): 40.8 years (13.9); 60.5% female	8/10
[80]	Attwood et al., 2016	UK	Qualitative	Semi-structured telephone interviews	PA counselling	16 patients; average age (SD) = 52,2 years (10,0); 63% female	7/10
[81]	Taylor et al., 2020	UK	Randomized controlled trial	accelerometry; questionnaires; semi-structured interviews	PA referral scheme	Quant: 450 participants; average age (SD) = 50 years (12); average BMI (SD) = 32.6 (4.4) kg/m^2^, with hypertension, prediabetes, type 2 diabetes, lower limb osteoarthritis or a current/recent history of treatment for depression, who were also inactive Qual: 26 participants; 76,9% female; age range: 28–69 years (females), 39–72 years males; health conditions: weight loss need, low mood, other morbidities	11/13
[82]	Hefnawi et al., 2021	Canada	Prevalence	self-reported online survey	PA counselling	38 physicians; 34.84 (13.54); 58% woman	2/9
[83]	Hanson et al. 2019	UK	Qualitative	longitudinal approach using semi-structured interviews	PA referral scheme	11 referred patients; NR; 63.6% woman; range of noncommunicable diseases, such as cardiovascular disease, mental health issues, diabetes, overweight/obesity, and musculoskeletal problems	8/10
[84]	Wattanapisit et al., 2019	Thailand	Qualitative	in-depth interviews	PA counselling	17 GPs; 29.8 ± 3.4 years; 64.7% woman	7/10
[85]	Omura et al., 2018	USA	Prevalence	web-based panel survey	PA counselling	1045 PCPs; 54.6% ≥ 45 years; 37.1% woman	3/9
[86]	Lewis et al., 2017	USA	Mixed-methods	Pedometer, electronic activity monitor, focus group	PA counselling	36 stakeholders; NR; NR	4/7
[87]	Brandborg et al., 2022	Denmark	Qualitative	semi-structured interviews	PA counselling and referral	9 GPs; 55.6% female; average age = 49 years (range: 40–60)	8/10
[88]	Bowen et al., 2021	USA, Alabama	Qualitative	semi-structured interviews	PA counselling	10 health care professionals (6 physicians; 4 nurse practitioners); average age (SD) = 59 (9.29) years; 40% females	8/10
[89]	Downey et al., 2021	UK	Qualitative	participant observation, interviews, document analysis, and reflexive journaling	PA referral scheme	8 practitioners	10/10
[90]	Huebschmann et al., 2022	USA	Mixed-methods	accelerometer steps/week; costs; interviews with patients and clinic coaches and clinicians	PA counselling	50 patients with type 2 diabetes—22 Control group: average age (SD) = 66.5 years (7.1); 59.1% females. 28 Intervention group: average age (SD) = 65.5 (7.6); 46.4% females	6/7
[91]	Dranebois et al., 2022	France (French Guiana)	Analytical cross sectional	questionnaire	PA counselling	73 GPs (42 men and 31 women); aged 27–73 years—median of 43 years (43% were under 40 years old; 17% were over 60 years old)	4/8
[92]	Morgan et al., 2021	UK	Qualitative	semi-structured interviews	PA referral scheme	50 individual stakeholders (9 scheme referrers, 22 scheme deliverers and 19 scheme participants);	8/10
[93]	Albert et al., 2021	Australia	Qualitative	semi-structured individual telephone interviews	PA referral scheme	8 GPs (0% female; average age = 44 years); 15 patients (80% female; average age = 61 years), and 17 Exercise Physiologists (65% female; average age = 31)	8/10
[94]	Wattanapisit et al., 2021	Thailand	Qualitative	focus group discussions	PA counselling	16 health care professionals (12 nurses; 3 public health officers; 1 physician); 93.8% females; median age = 38.5 years (range 24–56 years)	8/10
[95]	Calonge-Pascual et al., 2023	Spain	Qualitative	semi-structured group interviews	PA prescription	5 GPs and 5 nurses (randomly selected—the selection included males and females of different ages, with accredited PHC experience, representing both urban and rural areas of the region of Madrid)	8/10
[96]	Wangler & Jansky, 2023	Germany	Qualitative	semi-standardized interviews	PA counselling	76 GPs (38 male, 38 female); mean age: 54 years	7/10
[97]	Buckley et al., 2023	UK	Qualitative	group discussion and written reflections	PA referral scheme	5 stakeholders: 1 senior academic and exercise psychologist for the Co-PARS project; 1 exercise referral service user; 1 exercise referral practitioner; 1 fitness centre area manager; 1 GP and public health commissioner	9/10
[98]	Alyafei et al., 2023	Qatar	Quantitative	self-administered questionnaire	PA counselling	306 PHC physicians from the the 27 health centers ( from central, western, and northern regions of Qatar): 58.1% males; average age = 45.8 (7.9) years; 51.0% Family physician consultant, 30.2% GPs; average years of experience = 14 (8.3)	4/8
[99]	De Guzman et al., 2022	USA	Qualitative	open-ended survey, electronic medical record documentation (information on PA vital sign, PA prescription and primary care providers' discussion)	PA prescription	316 adult patients: 64.2% female; age categories—8.2% with 18–29 years, 35.1% with 30–49 years, 51.9% with 50–80 years, 4.7% with > 80 years	6/10
[100]	Moraes et al., 2022	Brazil	Quantitative	self-administered questionnaire	PA counselling	587 PHC professionals: 85.4% female; age groups—14.0% with 20–29 years, 68.8% with 30–49 years, 17.2% with 50 or more years; 87.7% from Family Health Team/Oral Health Team and 12.3% from Family Health Support Centers	7/8
[101]	Snége et al., 2022	Brazil	Quantitative	questionnaire applied by a health professional	Physical activity (sedentary behaviour) counselling	779 adult patients: 69.8% female; age groups—45.2% with 18–39 years, 36.9% with 40–59 years, 17.9% with 70 years or more; nr. Of chronic diseases—42.9% with 0, 39–9% with 1–2, 17.2 with 3 or more	7/8
[102]	Pellerine et al., 2022	Canada	Quantitative	self-reflection questionnaire; quantitative textual analysis in open-ended questions	PA counselling	114 PHC physicians: mean age = 52 (12) years; 54.9% women; 23 (13) years of practice 48 PHC nurses: mean age = 50 (10) years; 97.9% women; 27 (9) years of practice	4/9
[103]	Albert et al., 2022	Australia	Mixed-methods	questionnaires; semi-structured telephone interviews	PA referral scheme	Quantitative phase (Survey): 207 health professionals: 102 GPs (42% female; age groups—11% with 27 years or less, 30% with 28–37 years, 59% with 38 years or more); 105 exercise professionals (54% female; age groups—38% with 27 years or less, 43% with 28–37 years, 19% with 38 years or more) Qualitative phase (Interviews): 25 participants (8 GPs; 17 exercise professionals); 56% males	6/7

*BMI* body mass index, *GPs* general practitioners, *PA* physical activity, *PCP* primary care professionals, *PHC* primary health care, *SD* standard deviation

^a^Uniform demographic data not available for all references (data extracted as it is available in the articles)

Quality assessment of the studies is reported in Additional file 3. The main issues found in qualitative studies were the lack of a clear statement of the authors’ philosophical perspective, not addressing researcher’s cultural and theoretical location, as well as researcher-research influence. In mixed-methods studies, the main issue was the non-accomplishment of quality criteria for both study components (qualitative and quantitative). In analytical cross-sectional studies, the main issue was related to the validity and reliability of the instruments used. In prevalence studies, it was unclear whether health conditions were identified using validated methods, and there were also issues related with insufficient coverage of sample subgroups in data analysis. In quasi-experimental studies, the main issues were related to the absence of an independent control group and of a description and analysis of differences between groups at follow-up. As for the analysed randomized controlled trial, the only not fulfilled quality criteria was participants’ blinding.

### Barriers and facilitators to implementation of physical activity interventions in primary care

A total of 56 barriers and 55 facilitators to implementation were identified across the seven domains/themes. A supporting codebook, based on TICD framework [30], is available in Additional file 4 and a full list of these implementation determinants is organized in Table 2. Detailed data extraction information is available in Additional file 5.

**Table 2:** Barriers and facilitators to implementation of PA interventions in primary care and their reporting frequency

Theme/Domain	Implementation determinants	Reporting frequency			References
		**Total**	**By determinants' typology [Barrier (B) / Facilitator(F)]**		
**1.Intervention/ Guideline factors** Any factor of the intervention / program itself – it can be related to the scientific evidence, compatibility with regular tasks (feasibility in the way it is designed/how it fits the context), intervention’s materials, cost, and observability (the degree to which benefits of the recommended behaviour are visible). Is also includes factors associated with the intervention’s development and patients’ recruitment strategies	**(Lack of) Feasibility/compatibility**	32	**B**	28	[47–50, 52, 57–60, 62–64, 67, 69, 72, 78, 79, 84, 85, 87, 88, 91, 92, 94–97, 103]
			**F**	4	[46, 58, 88, 103]
	**Intervention components/ characteristics/ content**	22	**B**	5	[59, 62, 76, 78, 79]
			**F**	17	[49, 50, 54, 56, 58, 59, 67, 69, 70, 76, 79, 81, 84, 87, 96, 97, 99]
	**(Lack of) Evidence for effectiveness**	11	**B**	5	[62, 64, 72, 77, 78]
			**F**	6	[51, 54, 58, 77, 97, 103]
	**Devices/ technology**	10	**B**	4	[59, 81, 86, 94]
			**F**	6	[43, 50, 81, 90, 94, 96]
	**Tailored intervention/ patient-centred**	8	**B**	1	[99]
			**F**	7	[47, 54, 56, 67, 69, 80, 96]
	**Recruitment strategy**	6	**F**	6	[48, 56, 70, 80, 81, 92]
	**(Lack of) Clarity**	4	**B**	3	[52, 62, 92]
			**F**	1	[97]
	**(Lack of) Accessibility of the guideline/ recommendation**	3	**B**	1	[72]
			**F**	2	[48, 103]
	**(Lack of) Protocols**	3	**B**	1	[89]
			**F**	2	[89, 95]
	**(Lack of) Flexibility/ adaptability**	2	**B**	1	[59]
			**F**	1	[97]
	**Setting**	2	**B**	2	[80, 95]
**2.Individual health professional factors** Any factors related with the intervention’s deliverers / health professionals, including knowledge, skills, qualities, cognitions, attitudes, beliefs, motivation, and other characteristics need to implement the intervention	**Knowledge and skills**	50	**B**	28	[44–46, 48–52, 55, 57, 58, 60, 62, 64, 67, 69, 71, 72, 74, 76–79, 84, 85, 95, 96, 103]
			**F**	22	[44, 47, 50, 51, 53, 57–59, 62, 64, 67, 70, 71, 76–78, 82, 84, 89, 93, 97, 100]
	**Cognitions/ attitudes**	30	**B**	19	[44, 47–49, 51, 55–58, 64, 65, 68, 71, 72, 77, 85, 87, 91, 92]
			**F**	11	[45, 49, 56, 62, 64, 68, 69, 77, 82, 84, 91]
	**Professional behaviour**	18	**B**	5	[49, 51, 57, 60, 68]
			**F**	13	[43, 51, 56, 61, 68, 78, 83, 89, 90, 93, 94, 96, 100]
	**Scope of practice/ professional role**	13	**B**	11	[46–49, 55, 57, 67, 78, 82, 95, 96]
			**F**	2	[90, 95]
	**Health profile**	7	**B**	2	[51, 92]
			**F**	5	[44, 45, 53, 57, 92]
	**Professional profile**	5	**B**	2	[52, 57]
			**F**	3	[57, 61, 100]
	**Motivation**	4	**B**	1	[47]
			**F**	3	[44, 60, 67]
	**Sociodemographic characteristics**	4	**B**	1	[57]
			**F**	3	[57, 85, 98]
	**Established professional habits**	1	**B**	1	[78]
	**Expectations**	1	**B**	1	[60]
**3. Patient factors** Any factors related to the patients’/intervention’s recipients, such as needs, preferences, beliefs, behaviour, motivation, and engagement	**Motivation**	26	**B**	13	[44, 50, 52, 55, 57, 63, 64, 67, 72, 80, 82, 92, 103]
			**F**	13	[43, 49, 54, 56, 59, 69, 80, 83, 87, 90, 92, 96, 103]
	**Health status**	17	**B**	8	[56, 71, 72, 74, 80, 81, 83, 96]
			**F**	9	[61, 63, 66, 68, 74, 84, 88, 96, 101]
	**(Lack of) Compliance/engagement**	12	**B**	9	[48–50, 68, 81, 85, 87, 88, 91]
			**F**	3	[49, 68, 91]
	**Expectations**	11	**B**	9	[47, 54–57, 63, 79, 87, 90]
			**F**	2	[54, 66]
	**Preferences**	11	**B**	7	[49, 52, 64, 69, 72, 87, 88]
			**F**	4	[43, 49, 50, 65]
	**Beliefs and knowledge**	8	**B**	5	[71, 79, 80, 92, 95]
			**F**	3	[54, 78, 93]
	**Awareness/ attitudes**	8	**B**	6	[48, 49, 78, 83, 95, 99]
			**F**	2	[83, 92]
	**Behaviour and feedback**	8	**B**	5	[48, 50, 51, 57, 92]
			**F**	3	[92, 96, 101]
	**Sociodemographic characteristics**	6	**B**	2	[61, 91]
			**F**	4	[56, 66, 96, 101]
	**Adverse events and contingencies**	4	**B**	4	[78, 80, 81, 83]
	**Previous experiences**	3	**B**	2	[51, 83]
			**F**	1	[83]
	**Needs**	2	**B**	1	[49]
			**F**	1	[49]
	**Trust**	2	**F**	2	[56, 96]
**4. Professional interactions** Any factors related to professionals’ opinions and communication influences, local/regional networks, peer’s influences, system/organizational characteristics (teamwork, team interactions, etc.), and local collaborations with other partners	**Team processes (constraints)**	21	**B**	7	[47, 57, 62, 77, 95, 97, 103]
			**F**	14	[46, 51, 60, 65, 67, 75, 77, 87, 90, 91, 93, 95–97]
	**Networks**	20	**B**	7	[65, 67, 77, 95–97, 103]
			**F**	13	[49–51, 60, 65, 75, 77, 87, 91, 95–97, 100]
	**Team communication (constraints)**	10	**B**	3	[47, 51, 65]
			**F**	7	[47, 56, 60, 89, 92, 93, 103]
	**Referral processes (constraints)**	10	**B**	6	[47, 66, 67, 81, 97, 103]
			**F**	4	[47, 49, 81, 97]
	**Lack of mutual trust**	3	**B**	3	[60, 65, 87]
	**System/organizational characteristics**	1	**F**	1	[62]
**5. Incentives and resources** Any factors related to the necessary resources to implementation, namely financial and human resources, facilities, equipment, information system, and any other resources needed to implement the intervention (financial or non-financial). It also includes continuing education systems, assistance for clinicians, patient safety systems, and quality monitoring. It also includes trial incentives/disincentives	**(Cost and lack of) Financial incentives**	32	**B**	19	[44, 54, 56, 57, 62, 63, 65, 70, 72, 77, 81, 82, 85, 87, 91, 93, 95, 96, 103]
			**F**	13	[44, 54, 56, 62, 86, 87, 91–93, 95, 97, 102, 103]
	**Assistance tools and materials (constraints)**	31	**B**	15	[44, 49, 50, 57, 62–64, 67, 68, 75, 82, 94–96, 103]
			**F**	16	[42, 44, 51, 67, 68, 70, 73, 79, 86, 87, 90, 91, 95, 96, 102, 103]
	**(Lack of) Continuing education system**	15	**B**	4	[47, 63, 91, 95]
			**F**	11	[42, 62, 64, 73, 75, 91, 93, 95, 96, 102, 103]
	**Information system (constraints)**	14	**B**	4	[67, 68, 92, 95]
			**F**	10	[46, 47, 50, 60, 68, 75, 91, 92, 94, 95]
	**Physical activity opportunities (constraints)**	14	**B**	9	[44, 57, 60, 67, 85, 91, 95, 96, 103]
			**F**	5	[77, 96, 97, 99, 102]
	**Human resources (constraints)**	9	**B**	5	[44, 46, 50, 67, 77]
			**F**	4	[70, 79, 95, 102]
	**(Lack of) Team support/supervision**	5	**B**	2	[91, 96]
			**F**	3	[62, 94, 97]
	**Trial (des)incentives**	5	**B**	2	[50, 97]
			**F**	3	[80, 95, 97]
	**Health facilities (constraints)**	4	**B**	3	[50, 67, 95]
			**F**	1	[95]
	**Non-financial incentives**	2	**F**	2	[67, 103]
	**Patient safety systems**	1	**F**	1	[65]
**6. Capacity for organizational change** Any factors related to organizational characteristics which influence implementation, as mandate, decision making, leadership, organizational regulations/rules/policies, and the priority given to make necessary changes	**(Lack of) Capable leadership**	10	**B**	2	[62, 65]
			**F**	8	[51, 56, 62, 75, 89, 92, 93, 97]
	**System/organizational functioning**	5	**B**	3	[67, 80, 96]
			**F**	2	[62, 95]
	**(Lack of) Priority of necessary change**	4	**B**	2	[47, 48]
			**F**	2	[62, 77]
	**Organizational regulations, rules, policies**	3	**B**	3	[65, 67, 71]
	**Monitoring and feedback**	1	**F**	1	[65]
	**Lack of planning**	1	**B**	1	[89]
**7. Social, political, and legal factors** Any factors related to the social and political environment, including economic constraints and funding policies (macro budget), legislation, corruption, political stability, and political health agenda. It also includes geographic accessibility	**(Lack of) Funder policies**	5	**B**	2	[97, 103]
			**F**	3	[50, 96, 97]
	**(Economic constraints on the) health care budget**	5	**B**	3	[62, 91, 97]
			**F**	2	[95, 102]
	**Influential people**	4	**F**	4	[77, 91, 95, 102]
	**Geographic accessibility (constraints)**	4	**B**	3	[47, 81, 92]
			**F**	1	[51]
	**Legislation**	1	**F**	1	[44]
	**Public unsafety**	1	**B**	1	[56]
	**Neighbourhood socioeconomic profile**	1	**B**	1	[92]

#### Intervention/guideline factors

“(Lack of) feasibility/compatibility” and “intervention components/characteristics/content” were the most reported determinants within this domain.

The **absence of feasibility/compatibility** of PA interventions’ implementation within health professionals’ usual tasks and activities was a key highlighted barrier. Extended time was emphasized as a requirement to implement interventions regularly, while simultaneously addressing the primary reason for the patient’s visit and parallel professional demands and responsibilities. PA interventions requiring a more structured local organization (e.g., a specific PA consultation) were also associated to complex logistics (e.g., specific space, more time needed), more difficult to accommodate. Some studies [46, 58, 88] reported ways by which increasing feasibility/compatibility of the intervention would be a facilitator, for instance, transferring the implementation responsibility to health care professionals who have more consultation time (as dietitians or nurse practitioners).

Some “**intervention components/characteristics/content**” were reported as key facilitators, namely goal setting, action planning, self-monitoring and social support components. Interventions incorporating written prescriptions and regular follow-ups were also seen as facilitators, both by health care professionals and patients. On the other hand, complex methods requiring extensive knowledge by implementers and intervention activities considered chores by the patients (e.g., PA diaries) exemplify the barriers reported in primary studies.

Other *intervention/guideline factors* were less studied or reported. Evidence is suggestive of the potential facilitator role of “tailored intervention/patient-centred” and “recruitment strategy” used.

#### Individual health professional factors

“Knowledge and skills”, “cognitions/attitudes”, and “professional behaviour” were the most highlighted determinants within this domain.

Health professional’s “**knowledge and skills**” to promote PA was the most frequently reported/studied determinant, both as barrier and facilitator (50 times in 62 studies). The lack of training or expertise in the area of PA and behaviour change techniques, unfamiliarity with guidelines, lack of knowledge on safety issues concerning PA practice by people with chronic conditions, and unfamiliarity with suitable PA opportunities in the community illustrate the barriers highlighted by the studies’ participants. Receiving training in medical school about PA promotion, training the health care teams working in health surgeries, especially regarding PA promotion in chronically ill patients and in behaviour change techniques, and attending local activities with information about local PA offers were examples of reported facilitators.

Health professionals’ “**cognitions and attitudes**” were also reported both as barriers and as facilitators. Health professionals’ belief that PA is not a relevant and/or effective prevention strategy or treatment, giving it a low priority or finding other lifestyle changes more important, was reported in several studies as barriers. Having a good attitude towards PA promotion, an increased understanding of the importance of PA promotion in healthcare, perception of no barriers to counselling, and considering PA as an important behaviour for good health were in turn emphasised by health professionals as implementation facilitators.

Although less reported than the previous, “**professional behaviour**” was also frequently reported, especially as a facilitator. For instance, patients appreciated trustworthy, supportive, and non-judgmental advice by genuinely interested health professionals. A previous assessment of PA levels and patients’ readiness to change facilitated the implementation of PA counselling and prescription, according to health professionals. Feeling that patients’ PA promotion is outside their professional “**scope of practice/professional role**”, or that it is a role shared by all healthcare professionals and not exclusively by themselves was the third most highlighted barrier.

#### Patient factors

“Motivation” and “health status” were the two most frequently reported patient-related determinants, being considered both as barrier and facilitator.

Health care professionals perceiving lack of “**motivation**” by their patients was referred as a key barrier. From the patients’ view, no interest in receiving PA counselling was reported, for instance, when they felt they were already sufficiently active or when they already had pre-existing conditions requiring regular contact with health services and did not desire further testing. On the other hand, patients’ perception of PA positive effects on health, the social recognition and feelings of enjoyment derived from PA practice, contributed to their motivation, working as a facilitator.

Patients’ “**health status**”, namely some comorbidities, prevent patients to fully engage in the intervention, while in other cases, the “perceived threat” (e.g., type 2 diabetes) was not sufficient to mobilize change. For health professionals, patients’ illnesses, and the implementation of treatments other than PA competed for attention. Specifically, for some diseases, such as cancer, a significantly low proportion of health professionals recommended PA. On the other hand, health professionals were more likely to recommend PA to patients with overweight or obesity, type 2 diabetes or pre-diabetes, dyslipidaemia, and hypertension.

Although less studied/reported, two other determinants gathered evidence of relevance, as they were the second most reported barriers within this theme: health professionals perceived “**lack of compliance/engagement**” by patients and frustration of patients’ “**expectations**” (e.g., health professionals felt that some patients expected drug treatment instead of exercise, whereas other patients felt that the program was missing more intense exercise training options).

#### Professional interactions

*Professional interactions* were mainly reported as facilitators. “Team processes” and “networks” were the two most relevant, playing a key facilitating role in implementation.

Highlighted positive “**team processes**” were mainly related with a good cooperation between PA counsellors and health care professionals, or with a good functioning dynamic of the family health teams.

Another key facilitator was “**networks**”. Health professionals stressed the importance of a connection between sectors, which may result in increased referral of patients, and the importance of involving all stakeholders in a shared mission.

Although less studied/reported, “team communication (constraints)” and “referral processes (constraints)” were the third most reported determinants.

#### Incentives and resources

“(Cost and lack of) financial incentives” and “assistance tools and materials” were the most frequently highlighted determinants, both as barriers and as facilitators.

“**Cost and lack of financial incentives**” was often felt as a barrier. Patients and health professionals frequently reported expensive memberships in PA facilities for patients. Health professionals also highlighted the lack of financial reimbursements to implementers. Indeed, health professionals’ reimbursements of PA prescriptions and economic subsidies for patients to reduce the cost of joining an exercise facility, or even having a trial period before membership, were often reported as a “financial incentives” facilitator.

Regarding “**assistance tools and materials**” constraints, health professionals often highlighted lack of instructional material and effective tools and educational information to give to patients. On the other hand, the availability of specific intervention support tools and materials (e.g., practitioner toolbox; standardized and up-to-date information about where to refer patients, as a "community mapping” including PA facilities within the geographical area; decision algorithms) were believed to facilitate the implementation process, with technological tools being especially welcomed by health professionals.

Indeed, the “**information system**” was mainly reported as facilitator. Health professionals welcomed procedures’ digitalization to reduce time and money, namely through the integration of PA promotion tools in the electronic health system, as referral forms, prescription pads, and modules for PA counselling, for instance. Having access to patients’ interdisciplinary health care charts was also reported by health professionals to support tailored counselling.

Providing a “**continuing education system**” offer for health care staff (e.g., regarding PA promotion, its pathways and modes of delivery) was also highlighted as a relevant facilitator.

#### Capacity for organizational change

“**Capable leadership**” was the most frequently reported implementation determinant. Health professionals and stakeholders identified the election of a formal coordinator/leader, regularly present in the working group and providing support and updated information/knowledge sharing to the implementation team, as an implementation facilitating factor. Managers’ championing and endorsement of the intervention was also emphasized. Cases where the primary care management was not explicitly fulfilling this role hindered the implementation.

Other determinants within this theme were less studied.

#### Social, political and legal factors

Determinants within this domain were the least studied/reported. “**(Lack of) funder policies**” and “**(economic constraints on the) health care budget**” were reported in five studies, both as barriers and facilitators.

### Implementation determinants’ themes according to primary care physical activity intervention type

Table 3 provides a summary of the implementation determinants (main themes) reported in each intervention type.

**Table 3:** Reporting frequency of the main themes of implementation barriers and facilitators according to primary care intervention type

Main Themes		Reporting frequency according to intervention type						
		**Intervention Types**				**Combinations**		
		**PA assessment**	**PA counselling**	**PA prescription**	**PA referral scheme**	**PA assessment & counselling**	**PA counselling & prescription**	**PA counselling & referral**
**Barriers**	1. Intervention/ guideline factors	1	21	5	8	1	4	3
	2. Individual health professional factors	1	18	6	7	1	2	5
	3. Patient factors	1	20	8	8	-	3	4
	4. Professional interactions	-	5	2	5	-	-	5
	5. Incentives and resources	-	13	8	9	-	3	4
	6. Capacity for organizational change	-	6	1	2	-	-	1
	7. Social, political, and legal factors	-	1	1	5	-	-	1
**Facilitators**	1. Intervention/ guideline factors	1	15	5	9	1	-	5
	2. Individual health professional factors	1	20	7	9	1	1	4
	3. Patient factors	1	14	3	8	-	-	1
	4. Professional interactions	-	9	3	14	-	-	5
	5. Incentives and resources	1	10	9	7	-	1	5
	6. Capacity for organizational change	-	1	3	6	-	-	2
	7. Social, political, and legal factors	-	3	3	1	-	-	2

Legend: *PA* physical activity

Three interventions – PA counselling; PA prescription; PA referral schemes – and one combination – PA counselling and referral – gathered implementation barriers and facilitators from all domains, whereas those involving PA assessment seemed to be more influenced by determinants pertaining to intervention/guideline-, deliverers-, and patient-related factors. *Intervention/guideline factors* and *individual health professional factors* were reported in all intervention types and combinations, proving to be key determinants to consider when implementing PA interventions in primary healthcare. *Patient factors* and *incentives and resources’* barriers and facilitators were also central to implementation, being reported in the four intervention types. *Professional interactions*, *capacity for organizational change*, and *social, political, and legal factors* did not seem to be considered pivotal in implementation processes of simpler interventions, as PA assessment alone. These groups of determinants played a more relevant role in interventions with more complexity, requiring further delivering resources, as PA counselling, PA prescription, and those involving referral processes.

Considering the reporting frequency of the main themes by each intervention type, PA counselling implementation seems to be mainly hindered by factors related to the intervention/guideline, individual health professionals and patients, and mainly facilitated by individual health professional factors. PA prescription implementation seems to be particularly influenced by barriers and facilitators pertaining incentives and resources, whereas PA referral schemes are predominantly facilitated by factors related to professional interactions.

### Certainty assessment of the systematic review

The SURE tool indicated that this is a good quality systematic review with minor limitations regarding selection procedure: i. language bias, as only studies written in English were selected; and ii. status of publication, as only published studies were included (see Additional file 6). A more comprehensive search avoiding these limitations could, thus, have retrieved a higher number of studies. Even so, English is the universal language for science communication, the best available science works tend to be published, and a seven-year time interval can be considered adequate to have an updated picture of today’s health services panorama. Considering the critical appraisal of the included studies and that the output of this systematic review is qualitative, the three quality criteria that probably most negatively influence certainty of the evidence were the non-accomplishment of quality criteria for both study components (qualitative and quantitative) in mixed-methods studies, issues related with the validity and reliability of the instruments used in analytical cross-sectional studies, as well as insufficient coverage of sample subgroups in data analysis in some prevalence studies. However, it is important to stress that the vast majority of the included studies did not present any of these issues. Together, the findings of the present systematic review can be considered reliable for evidence-informed health policymaking. Results of this review should, nevertheless, be interpreted taking these minor limitations into consideration.

## Discussion

This systematic review assessed implementation barriers and facilitators in real-world PA promotion and/or sedentary time reduction interventions (i.e., PA assessment, brief counselling, prescription, and referral scheme) delivered in primary healthcare settings, using the TICD framework [30]. Five determinants of implementation success stood out from our review, given their reported frequency: having health professionals with a good degree of knowledge and skills regarding PA and its promotion; the need for the intervention to be feasible/compatible with professionals’ and health services’ usual tasks; interventions’ cost and the provision of financial incentives; having adequate tools and materials to implement the intervention; and fostering positive health professionals’ cognitions and attitudes, while minimizing negative ones. These determinants belong to three domains: *individual health professional factors*; *intervention/guideline factors*, and *incentives and resources*. Despite being less or rarely reported, other determinants may play a particularly facilitating or hindering role regarding interventions’ implementation (e.g., networks). Apart from PA assessment, implementation of all intervention types (excluding combinations) is influenced by factors belonging to all the seven main domains, although some domains were predominantly highlighted in a certain type of intervention: PA counselling seems to be particularly hampered by intervention/guideline and individual (health professionals and patients) factors and facilitated by individual health professionals’ ones; PA prescription seems to be particularly influenced by incentives and resources’ barriers and facilitators; and PA referral schemes seem to be specially facilitated by factors related to professional interactions. PA assessment seems to be more dependent on individual factors (from patients or professionals) and available resources – whereas more complex interventions seem to rely also on organisational, political, and social determinants –, but the limited number of primary studies assessing PA assessment alone can be biasing this specific result.

Health professionals’ knowledge and skills was the most frequent reported determinant and has been previously highlighted as important for proper implementation [13, 32, 33, 35, 105]. WHO’s monitoring of the implementation of the Global Action Plan for Physical Activity also reinforced that more pre- and post-graduated training of health professionals is needed – also for professionals outside the health sector – combined with the provision of adequate tools and guidance [3]. However, training is not always sufficient to determine health professionals’ PA counselling behaviours [106, 107]. Despite this, PA promotion in medical schools’ curricula is still a hot topic, as there seems to be a recurrent gap in the pre-graduate medical training [108–110]. The importance of knowing PA pathways to community resources and behaviour change techniques was mentioned in several works. This reinforces the need for proper training of health professionals, not only in terms of PA content, but also in modes of delivery. Adequate and innovative information systems may be promising tools in supporting face-to-face delivery of behaviour change techniques applied to PA promotion [111]. A continuing education system that can support in-service professionals (the third most reported facilitator within *incentives and resources*’ theme) can also play a relevant role in this regard.

Concerning interventions’ feasibility/compatibility, a recent systematic review on the views of stakeholders also identified the congruence of the intervention with team activities as key facilitator [32]. The (lack of) compatibility of the intervention with usual tasks may be interrelated with other reported determinants (for instance, having enough human resources). Of these, a significant one is the optimization of the information system, the second most reported facilitator within the “incentives and resources” domain. Indeed, the availability of computerised solutions that help health professionals save time and efforts during interventions’ delivery may be, once more, paramount.

Interventions’ cost has long been a concern regarding PA promotion in primary care and health system sustainability. Particularly, PA counselling and referral brief interventions are very well positioned to be nationally/locally endorsed, as they are considered a “best-buy” to tackle non-communicable diseases, giving their evidence of cost-effectiveness [10, 112]. Financial incentives for patients have also gathered evidence in increasing patients’ PA in the short and long term [113], which can be an effect of an increased patients’ adherence to the intervention. The establishment of networks between healthcare and community PA programmes and resources that brings reduced costs or even free PA options for patients can offer a solution in this regard. Also, a specific budget allocated to health-enhancing physical activity promotion is considered strategic [114]. Financial incentives for healthcare professionals could, thus, be analysed in this context.

Adequate assistance tools and materials and health professionals’ cognitions and attitudes were also found to be key determinants. This result was shown in other works [32, 33], including community-based interventions [31]. Positive attitudes were linked with patients engagement and facilitated adaptation processes throughout implementation, whereas placing low value on the intervention hindered the implementation [31]. The relevance attributed to PA promotion in healthcare by medical doctors had also been identified as a significant predictor of clinical practice in this area [106].

“Social, political, and legal factors” were the least reported domain. Considering that national public health policy and legislation is recognized as crucial by international guidelines [9], this finding may reflect the scarcity of research specifically addressing health policy/legislation impact in this area. In fact, only one of the included studies [44] assessed the impact of a legislative framework on PA prescription.

Although the frequency of reporting is useful to obtain a picture of the most and least studied implementation determinants, it does not necessarily reflect the degree of importance of each barrier and facilitator. Caution is needed, as interpretation bias may be introduced if one equates the relevance of each determinant with its reporting frequency. Even so, the identified implementation determinants were under the seven domains of the TICD framework, with even distribution between barriers and facilitators in each domain, evidencing that the studies included explored an extensive set of factors influencing implementation.

This review presents suggestive evidence that other determinants may play an important role and should not be overlooked: patients’ motivation (barrier/facilitator); intervention components/characteristics/content (facilitator); positive team processes (facilitator); and the establishment of networks between sectors/stakeholders (facilitator). Having the knowledge and skills to implement an intervention evidencing compatibility/feasibility with routine care does not mean that implementation cannot be easily hindered by other determinants in place. Together, this evidence suggests that there are some more general implementation determinants and others more context-specific. A broad assessment of implementation barriers and facilitators should, thus, be made when preparing an intervention implementation to understand the local context.

The entire chain of interacting actors within and outside the health sector, influences implementation success. Each one brings unique contributions to the implementation and scaling-up phases. Planning beforehand to identify and engage all relevant stakeholders from the entire delivery chain is of outmost importance to tackle future translational challenges. Nonetheless, primary studies often overlooked the views of politicians, health coordinators or community stakeholders, suggesting an evidence gap. The need for a coordinated systems-approach to foster the implementation of PA interventions in healthcare settings, involving several key stakeholders, has been reported in multiple works in this area [13, 105, 115–117].

Another finding was that adequate implementation of more complex interventions implies the commitment of more structures, beyond the specific contexts of local health facilities, professionals and patients. In line with the “PA vital sign” proposal [118], it can be hypothesised that the universal implementation of PA assessment should be the first step for PA promotion in primary care, with the more complex ones being gradually introduced. Implementing PA assessment was even reported in primary studies as a facilitator of the subsequent implementation of PA counselling. However, the limited number of primary studies addressing PA assessment alone do not allow to draw firm conclusions on this issue.

Generating knowledge about key implementation barriers and facilitators of PA promotion interventions in primary healthcare contributes to define tailored implementation strategies to improve the adoption, implementation, sustainability, and scaling-up of such interventions [23]. An iterative planning process should occur to potentiate success: 1) characterizing the delivery context and anticipating barriers and facilitators; 2) designing tailored implementation strategies; 3) monitoring implementation and dealing with implementation determinants that effectively emerge during translation and scale-up; and 4) incorporating these outcomes in the implementation processes to optimize them [119–121].

### Strengths and limitations

To our knowledge, this is the first systematic review analysing theoretically framed implementation barriers and facilitators of four PA interventions (assessment, counselling, prescription, referral) implemented in the primary health care, integrating the views of patients, health professionals and stakeholders. The framework used herein to systematize barriers and facilitators of implementation also constitutes a strength of this review, as it was specifically developed to identify determinants of practice in healthcare contexts, facilitating its identification and organisation in a parsimonious way.

Still, this review is not without limitations. Attention should be paid to the fact that more than one third of the included studies used quantitative designs. As such, some determinants may be intentionally selected and more frequently studied by researchers (e.g., in questionnaires with closed-ended questions), as opposed to implementation determinants that unintentionally emerge from qualitative data. Furthermore, only 31% of the primary studies clearly reported the use of a published framework when identifying implementation determinants, which presents a high risk of bias, as acknowledged barriers and facilitators could have been overlooked. Also, further studies including the views of stakeholders, outside the health sector, remain scarce, precluding a more comprehensive picture of implementation determinants. Most studies included in this systematic review reflect interventions implemented in high income countries, suggesting that the findings presented may not necessarily play a similar role in implementation processes occurring in countries of other income levels. Also, lack of sufficient detail in studies’ description of the PA promotion interventions was common, which may have led to an incorrect classification of the interventions. Earlier described methodological limitations of the primary studies are also concerning factors, as they could have biased the results. Lastly, the time limitation of the literature search poses a methodological limitation, as studies published before 2016 were not considered. Despite this, and together with the reasonable number of included studies obtained (*n* = 62), a fair picture of today’s reality of implementation determinants of PA promotion interventions in primary care was probably achieved. Caution is needed, however, when analysing the results for PA assessment, as only two primary studies addressed this type of intervention alone.

### Future research

In order to bridge the gap between research and practice, future research should focus on proper implementation preparation of evidence-based interventions and enhanced dissemination, considering: a) the wide range of agents that should be involved (stakeholders from all levels); b) implementation barriers and facilitators, considering mixed-methods design studies (combining quantitative components, that estimate the degree of influence of each determinant in real-world conditions, with qualitative components that allow the identification of potential barriers and facilitators), with proper interventions’ descriptions, and investing in studies of interventions also delivered in upper middle and low income contexts; c) tailored implementation strategies and implementation plans. In implementing interventions in real-world conditions, an adaptation phase should always be expected, involving constant loops of monitoring and feedback to increase the effect, aligning with the evidence, while fully embed the intervention in a new system and carefully keeping its active ingredients – future research agenda should support these processes as well.

## Conclusion

The present review identifies the most relevant implementation determinants of PA-specific promotion interventions in primary health care, from the point of view of health professionals, patients, and stakeholders. These findings address a research-to-practice gap and will support the translation process of science-based interventions.

Although implementation of PA promotion interventions in primary care is determined by a wide set of barriers and facilitators, health professionals-, intervention-, and resources-specific ones seem to be particularly relevant. As such, a careful consideration of these factors is needed when preparing interventions’ delivery. Tailored implementation strategies should be designed for successful implementation, particularly those addressing deliverers’ knowledge/skills, attitudes and cognitions; interventions’ feasibility/compatibility with routine care and cost; and the availability of adequate supporting materials and tools. Suggestive evidence also highlights some barriers and facilitators related with patients’ motivation, intervention characteristics, and professionals’ interactions as relevant. Moreover, implementation determinants are modulated by the type of PA intervention. From a practical implication perspective, there seems to be more context- and intervention-specific determinants, so a deep understanding of the local context combined with intervention’s characteristics is highly recommended when preparing an intervention implementation.

The findings of this review should be considered by primary care authorities and coordination teams aiming to optimize interventions’ implementation and effectiveness in real world conditions – from the design of tailored implementation strategies to the development of national policies, tools and systems to support regional or nationwide scale-up.

### Registration and protocol

This systematic review was registered in PROSPERO (CRD42022318632). The protocol was not previously published.

### Supplementary Information

**Additional file 1.** PRISMA 2020 Checklist.

**Additional file 2.** Search steam.

**Additional file 3: **Critical appraisal of the included studies.

**Additional file 4: **Codebook of the implementation determinants.

**Additional file 5: **Detailed report of implementation determinants, with supporting extracted data.

**Additional file 6.** Certainty assessment of the systematic review (SURE checklist).

## Data Availability

All relevant data used for the current study are within the paper and its supporting information.

## Abbreviations

JBI: Joanna Brigs Institute
MMAT: Mixed-Methods Appraisal Tool
PA: Physical activity
PRISMA: Preferred Reporting Items for Systematic reviews and Meta-Analyses
SURE: Supporting the Use of Research Evidence
TICD: Tailored Implementation for Chronic Diseases
WHO: World Health Organization
