# Supplementary material for: Implementation determinants of physical activity interventions in primary health care settings using the TICD framework: a systematic review
Source: BMC Health Serv Res. 2023 Oct 11;23:1082. doi: 10.1186/s12913-023-09881-y (PMC10568782; doi:10.1186/s12913-023-09881-y)
Supplement: Supplementary file 2 — Additional file 2. Search steam. [file 12913_2023_9881_MOESM2_ESM.pdf]

S2 File – Search steam

TITLE-ABS(("physical activity" OR "physical inactivity" OR exercis* OR "sedentary behav*" OR "sitting time") AND (counsel* OR advice OR "brief intervention*" OR "brief advice" OR "brief counsel*" OR prescription* OR referral OR consultation OR "physical activity screening" OR "physical activity assessment") AND ("primary care" OR "primary health care" OR "primary healthcare" OR "general practice*" OR "general practitioner*" OR "nurse practitioner*" OR "family physician*" OR midwives OR physiotherapist* OR "exercise professional*" OR "exercise physiologist*") AND (barriers OR impediment* OR obstacle* OR hurdle* OR hindrance* OR challenge* OR facilitat* OR enabler* OR implement* OR determinants OR "implementation evaluation" OR "process evaluation" OR process* OR "qualitative evaluation" OR "qualitative study" OR "qualitative research" OR qualitative OR "mixed method*" OR "mixed-method*" OR perspectives OR experienc* OR translat* OR interview* OR "focus group*")).
