# Supplementary material for: Implementation determinants of physical activity interventions in primary health care settings using the TICD framework: a systematic review
Source: BMC Health Serv Res. 2023 Oct 11;23:1082. doi: 10.1186/s12913-023-09881-y (PMC10568782; doi:10.1186/s12913-023-09881-y)
Supplement: Supplementary file 3 — Additional file 3: Critical appraisal of the included studies. [file 12913_2023_9881_MOESM3_ESM.pdf]

S3 Table – Critical appraisal of the included studies.

	**Randomized Controlled Trials**													
Manuscript Reference	Study Citation	1.Randomization	2.Group allocation concealing	3.Baseline similarity of groups	4.Participants' blinding	5.Deliverers' blinding	6.Outcome assessors' blinding	7.Identically treated groups (other than intervention)	8.Follow-up completion and differences	9.Participants' analysis according to randomization	10.Similar outcome measurement between groups	11.Reliability of outcome measurement	12.Statistical analysis	13.Design and deviations
(79)	Taylor et al., 2020	Y	Y	Y	N	Y	Y	Y	Y	Y	Y	Y	Y	U

	**Qualitative studies**										
Manuscript Reference	Study citation	1.Philosophical perspective and methodology congruity	2.Methodology and research question/objectives congruity	3.Methodology and collection methods congruity	4.Methodology and representation and analysis of data congruity	5.Methodology and interpretation of results congruity	6.Reasearcher's cultural and theoretical location	7.Researcher-research influence	8.Participants' representation	9.Ethics	10.Conclusions drawning from data
(41)	Syrjälä et al., 2021	U	Y	Y	Y	Y	N	U	Y	Y	Y
(44)	Huntington et al., 2020	U	Y	Y	Y	Y	N	Y	Y	Y	Y
(46)	Sissons et al., 2020	U	N	Y	Y	Y	N	N	Y	Y	Y
(47)	Carstairs et al., 2020	U	Y	Y	Y	Y	N	Y	Y	Y	Y
(52)	Joelsson et al., 2020	U	Y	Y	Y	Y	N	Y	Y	Y	Y
(53)	Christiansen et al., 2020	Y	Y	Y	Y	Y	N	U	Y	N	Y
(56)	Westland et al., 2018	U	Y	Y	Y	Y	N	Y	Y	Y	Y
(58)	Leenaars et al., 2018	U	Y	Y	Y	Y	N	U	Y	N	Y
(60)	Gustavsson et al., 2018	U	Y	Y	Y	Y	N	U	Y	Y	Y
(63)	Henderson et al., 2018	Y	Y	Y	Y	Y	Y	Y	Y	Y	Y
(65)	Alghafri et al., 2017	Y	Y	Y	Y	Y	Y	U	Y	Y	Y
(66)	Bélanger et al., 2017	U	Y	Y	Y	Y	N	N	Y	Y	Y
(69)	Matthews et al., 2017	U	Y	Y	Y	Y	Y	U	Y	N	Y
(74)	Dutton et al., 2016	U	Y	Y	Y	Y	N	N	Y	Y	Y
(75)	Leenaars et al., 2016	U	Y	Y	Y	Y	N	N	Y	Y	Y
(76)	Avery et al., 2016	U	Y	Y	Y	Y	N	N	N	Y	Y
(77)	Clark et al., 2021	U	Y	Y	Y	Y	N	Y	Y	Y	Y
(78)	Attwood et al., 2016	U	Y	Y	Y	Y	N	N	Y	Y	Y
(81)	Hanson et al., 2019	U	Y	Y	Y	Y	N	Y	Y	Y	Y

(82)	Wattanapisit et al., 2019	U	Y	Y	Y	Y	N	U	Y	Y	Y
(85)	Brandborg et al., 2022	Y	Y	Y	Y	Y	N	Y	Y	N	Y
(87)	Downey et al. 2021	Y	Y	Y	Y	Y	Y	Y	Y	Y	Y
(90)	Morgan et al., 2021	Y	Y	Y	Y	Y	N	N	Y	Y	Y
(91)	Albert et al., 2021	Y	Y	Y	Y	Y	N	N	Y	Y	Y
(92)	Wattanapisit et al., 2021	Y	Y	Y	Y	Y	N	N	Y	Y	Y
(86)	Bowen et al. 2021	Y	Y	Y	Y	Y	N	Y	Y	N	Y
(95)	Calonge-Pascual et al., 2023	Y	Y	Y	Y	Y	N	N	Y	Y	Y
(96)	Wangler & Jansky, 2023	U	Y	Y	Y	Y	N	N	Y	Y	Y
(97)	Buckley et al., 2023	Y	Y	Y	Y	Y	Y	Y	Y	N	Y
(99)	De Guzman et al., 2022	U	U	Y	Y	Y	N	N	Y	Y	Y
	**Quasi-experimental studies (includes pre-post design studies)**										
Manuscript Reference	Study citation	1.Clear cause vs. effect	2.Groups similarity	3.Treatment/care similarity (other than the intervention)	4.Control group	5.Pre and post outcome measurement	6.Follow up completion and differences	7.Outcome measurement similarity	8.Reliability of outcome measurent	9.Statistical analysis	
(40)	Kyei-Frimpong et al., 2021	Y	Y	N	N	Y	N	Y	U	N	
(62)	Fowles et al., 2018	Y	Y	Y	U	Y	N	Y	U	U	
(70)	O'Brian et al., 2017	Y	Y	Y	N	U	N	U	U	U	
(71)	Leavitt, 2017	Y	Y	U	N	Y	U	Y	U	U	
(73)	Aittasalo et al., 2016	Y	Y	N	N	Y	U	Y	U	U	
	**Prevalence studies (includes descriptive cross sectional studies)**										
Manuscript Reference	Study citation	1.Sample frame	2.Sampling	3.Sample size	4.Subjects and setting description	5.Data analysis coverage	6.Validity of methods to identify the condition	7.Standardization and reliability of condition measurement	8.Statistical analysis	9.Response rate	
(51)	Agadayi et al., 2019	N	N	U	Y	N	N	Y	U	U	
(61)	Al-Ghamdi et al., 2018	Y	Y	Y	Y	U	N	Y	U	Y	
(67)	Cottrell et al., 2017	Y	Y	Y	Y	Y	N	Y	Y	Y	
(72)	Hidalgo et al., 2016	Y	Y	N	Y	N	N	Y	N	N	
(80)	Hefnawi et al., 2021	N	N	N	Y	N	N	Y	U	N	
(83)	Omura et al., 2018	N	N	U	Y	U	N	Y	Y	N	
(102)	Pellerine et al., 2022	N	N	Y	Y	U	N	Y	N	Y	

Manuscript Reference	Study citation	1.Inclusion criteria	2.Subjects and setting description	3.Validity and reliability of exposure measurement	4.Objective and standard criteria for condition measurement	5.Confounding factors identification	6.Confounding factors dealing	7.Validity and reliability of outcomes measurement	8.Statisitcal analysis
	**Analytical cross sectional studies**								
(42)	Charles et al., 2022	Y	Y	NA	N	Y	Y	N	Y
(43)	Souza-Neto et al., 2021	Y	Y	NA	Y	Y	Y	U	Y
(50)	Beni-Yonis et al., 2020	Y	Y	NA	Y	Y	Y	N	Y
(55)	Alahmed & Lobelo, 2019	Y	Y	Y	Y	U	U	N	Y
(59)	Baillot et al., 2018	Y	Y	N	Y	U	U	N	Y
(64)	Johnson et al., 2018	Y	Y	U	U	Y	Y	Y	Y
(89)	Dranebois et al. 2022	Y	Y	NA	Y	N	N	N	Y
(98)	Alyafei et al., 2023	Y	Y	NA	Y	Y	N	N	N
(100)	Moraes et al., 2022	Y	Y	NA	Y	Y	Y	Y	Y
(101)	Snége et al., 2022	Y	Y	NA	Y	Y	Y	Y	Y

Manuscript Reference	Study citation	1.Clear research question(s)	2.Collected data addresses research question(s)	3.Adequate rationale vs. research question(s) for using a mixed methods design	4.Integration of different study components	5.Output interpretation of the integration of both components (qualitative and quantitative)	6.Addressing of divergencies between qualitative and quantitative results	7.Methods quality criteria of the different study components
	**Mixed-methods studies**							
(45)	Buckley et al., 2020	Y	Y	Y	N	U	U	N
(48)	Alghafri et al., 2020	Y	Y	Y	Y	Y	Y	N
(49)	Parjanen, 2021	Y	Y	Y	Y	Y	Y	Y
(54)	Bird et al., 2019	Y	Y	Y	Y	Y	Y	Y
(57)	Harris et al., 2018	Y	Y	Y	Y	Y	Y	Y
(68)	Barrett et al., 2017	Y	Y	N	Y	Y	Y	N
(84)	Lewis et al., 2017	Y	Y	U	Y	Y	U	N
(88)	Huebschmann et al., 2022	Y	Y	Y	Y	Y	Y	N
(103)	Albert et al., 2022	Y	Y	Y	Y	Y	Y	N

Y, yes; N, no; U, unclear; NA, not applicable
