# Supplementary material for: Implementation determinants of physical activity interventions in primary health care settings using the TICD framework: a systematic review
Source: BMC Health Serv Res. 2023 Oct 11;23:1082. doi: 10.1186/s12913-023-09881-y (PMC10568782; doi:10.1186/s12913-023-09881-y)
Supplement: Supplementary file 4 — Additional file 4: Codebook of the implementation determinants. [file 12913_2023_9881_MOESM4_ESM.pdf]

S4 Table – Codebook of the implementation determinants.

Domain/Main theme	Subtheme	
**1.Intervention/ Guideline factors**<br><br>Any factor of the intervention / program itself – it can be related to the scientific evidence, compatibility with regular tasks (feasibility in the way it is designed/how it fits the context), intervention's materials, cost, and observability (the degree to which benefits of the recommended behaviour are visible). Is also includes factors associated with the intervention's development and patients' recruitment strategies.	**(Lack of) Evidence for effectiveness**	Any factor related to the existence or absence of scientific evidence concerning the intervention/ guideline for physical activity promotion and/or physical activity for health.
	**(Lack of) Tailored intervention/ patient-centred**	The degree in which the intervention/ guideline is or not tailored to the patient needs and characteristics.
	**(Lack of) Feasibility/compatibility**	The degree in which the intervention/ guideline is compatible with health surgeries' and health professionals' usual tasks and activities. It includes available time to implement the intervention.
	**Recruitment strategy**	The degree in which the strategy or methods chosen for patients' recruitment promote or hinders the implementation.
	**Intervention components/ characteristics/ content**	Any intervention characteristic or component which promotes or hinders the implementation (e.g. intervention "active ingredients, intervention resources, etc.).
	**(Lack of) Clarity**	The degree of clarity of the intervention procedures or guidelines for implementers in a way influencing the implementation itself.
	**(Lack of) Flexibility/ adaptability**	The possibility or not of adapting some procedures of the intervention implementation and the way it influences the degree of the implementation itself.
	**(Lack of) Accessibility of the guideline/ recommendation**	The degree in which the guideline or recommendation is available to the implementers.
	**(Lack of) Protocols**	The way the availability or absence of protocols influences the implementation.
	**Setting**	The way the setting in which the guideline or intervention is implemented enables or hinders implementation.
	**Devices/ technology**	Any intervention's devices or technology used that influences the implementation of the intervention itself.
**2.Individual health professional factors**<br><br>Any factors related with the intervention's deliverers / health professionals, including knowledge, skills, qualities, cognitions, attitudes, beliefs, motivation, and other characteristics need to implement the intervention.	**Professional behaviour**	Professional behaviours or types of interaction adopted by the implementors.
	**Health profile**	Health status and other individual health characteristics (or personal health behaviours) of the implementers.
	**Cognitions/ attitudes**	Any cognition, attitude, or perspective of the health professionals related to any characteristic of the intervention or its implementation.
	**Knowledge and skills**	The degree of knowledge and skills of the health professionals necessary to the implementation, including the need to improve them.
	**Professional profile**	Professional characteristics (profession, specialty, etc.)
	**Established professional habits**	Usual professional functions or habits.
	**Scope of practice/ professional role**	The degree in which the intervention/guideline implementation is within the scope of practice of the health professionals. It includes the way the health professional itsef *feels* that the implementation is (or is not) part of his/her professional role.
	**Motivation**	The health professionals' motivation to implement the intervention/guideline.
	**Sociodemographic characteristics**	Any sociodemographic characteristics of the health professionals associated with better or worst levels of implementation.
	**Expectations**	Any expectations of the health professionals related to the intervention/ guideline implementation.
**3. Patient factors**<br><br>Any factors related to the patients'/intervention's recipients, such as needs, preferences, beliefs, behaviour, motivation, and engagement.	**Expectations**	Any patients' expectations related to the intervention/ guideline implementation.
	**Awareness/ attitudes**	The degree to which the patient is aware of physical activity importance, as well as about the existence of the intervention. It also includes patients' attitudes regarding physical activity and its promotion.
	**Motivation**	The patients' motivation related to the adherence to the intervention / guideline implementation.
	**Needs**	Real or perceived needs or demands of the patient
	**Behaviour and feedback**	Patients' behaviours and/or feedback that promotes or hinder implementation or adherence.
	**(Lack of) Compliance/engagement**	Real or perceived patients' involvement in the intervention.
	**Health status**	Health indicators or health profile of the patients.
	**Adverse events and contingencies**	Any unpredictable life event of the patient which influences adherence or implementation.
	**Preferences**	Patients' values and preferences.
	**Beliefs and knowledge**	Real or perceived patients' beliefs, knowledge, ability or skills in relation with guideline/intervention.
	**Sociodemographic characteristics**	Any sociodemographic characteristics of the patients associated with better or worst levels of implementation.
	**Previous experiences**	Patients' previous physical activity or intervention experiences influencing their adherence to the guideline/intervention.
	**(Lack of) Trust**	Patients' trust and confidence in the process/intervention and/or in the healthcare professionals responsible for the intervention implementation.
**4. Professional interactions**<br><br>Any factors related to professionals' opinions and communication influences, local/regional networks, peer's influences, system/organizational characteristics	**Networks**	Work relationships between different sectors or entities, besides health surgeries, regarding the implementation of the guideline or intervention.
	**Team processes (constraints)**	The way team interactions and processes related to the implementation hinders or facilitates implementation.
	**Team communication (constraints)**	Communication flow between the professionals of the implementation team.

(teamwork, team interactions, etc.), and local collaborations with other partners.	**Referral processes (constraints)**	The way patients' referral between professionals hinders or facilitates implementation.
	**(Lack of) Mutual trust**	Trust between professionals or entities involved in the intervention, regarding the necessary abilities and responsibilities for its implementation.
**5. Incentives and resources**<br><br>Any factors related to the necessary resources to implementation, namely financial and human resources, facilities, equipment, information system, and any other resources needed to implement the intervention (financial or non-financial). It also includes continuing education systems, assistance for clinicians, patient safety systems, and quality monitoring. It also includes trial incentives/desincentives	**(Lack of) Continuing education system**	The way the characteristics of the continuing education systems of the graduated health professionals influences implementation.
	**Non-financial (des)incentives**	Real or perceived incentives not related with financial topics influencing patients/professionals adherence or implementation (e.g. existence of health groups).
	**(Cost and lack of) Financial incentives**	Real or perceived incentives or disincentives related with financial topics influencing patients/professionals adherence or implementation.
	**Assistance tools and materials (constrainsts)**	Tools and materials considered to assist professionals and patients regarding intervention/guideline implementation.
	**Human resources (constrainsts)**	The extent to which human resources needed are available.
	**Physical activity opportunities (constraints)**	The extent to which physical activity opportunities (programmes, community resources, etc.) are available.
	**Health facilities (constraints)**	Physical facilities of the health surgeries (e.g. proper/dedicated spaces; physical challenges, etc.).
	**(Lack of) Team support/supervision**	Available of support and/or supervision of the implementation teams, regarding intervention/guideline implementation.
	**Patient safety systems**	The extent to which patient safety systems influences adherence and overall implementation.
	**Trial (des)incentives**	The extent to which an eventual coupled trial and its procedures influence adherence and overall intervention implementation.
	**Information system (constrainsts)**	Characteristics of the digital health information system influencing implementation (e.g. limitations, resources, improvements needed, etc.)
**6. Capacity for organizational change**<br><br>Any factors related to organizational characteristics which influence implementation, as mandate, decision making, leadership, organizational regulations/rules/policies, and the priority given to make necessary changes.	**(Lack of) Capable leadership**	Health management leadership regarding guideline/intervention implementation (e.g. superior decisions, central meetings, updates on changes, etc.).
	**System/organizational functioning**	The extent to which local work routines and mode of operation influences adherence and overall implementation.
	**(Lack of) Priority of necessary change**	The relative local strategic priorities for health care for making necessary changes for guideline/intervention implementation.
	**Organizational regulations, rules, policies**	Local health regulations, clinical standards, treatment directions, rules, and policies adopted.
	**(Lack of) Monitoring and feedback**	Monitoring procedures and feedback provision about service quality or improvements needed.
	**(Lack of) Planning**	Intervention/guideline implementation planning procedures.
**7. Social, political, and legal factors**<br><br>Any factors related to the social and political environment, including economic constraints and funding policies (macro budget), legislation, corruption, political stability, and political health agenda. It also includes geographic accessibility.	**(Lack of) Legislation**	The extent to which legislation influences guideline/intervention implementation and necessary changes.
	**(Lack of) Funder policies**	The extent to which national funder policies or budgets influence guideline/intervention implementation and necessary changes.
	**Geographic accessibility (constraints)**	Real or perceived patients' difficulties or easiness in accessing health facilities or community physical activity resources.
	**Public (un)safety**	Real or perceived public safety and the extent it influences adherence and implementation.
	**Neighborhood socioeconomic profile**	The extent to which neighborhoods' socioeconomic profile (e.g. areas of deprivation) influences adherence or intervention implementation.
	**(Economic constraints on the) health care budget**	National financial constraints or enablers related to health care budget.
	**Influential people**	The extent to which influential people or organizations (outside targeted healthcare organizations) influence guideline/intervention implementation (e.g. municipalities, mass media, etc.).
