# Supplementary material for: Implementation determinants of physical activity interventions in primary health care settings using the TICD framework: a systematic review
Source: BMC Health Serv Res. 2023 Oct 11;23:1082. doi: 10.1186/s12913-023-09881-y (PMC10568782; doi:10.1186/s12913-023-09881-y)
Supplement: Supplementary file 5 — Additional file 5: Detailed report of implementation determinants, with supporting extracted data. [file 12913_2023_9881_MOESM5_ESM.pdf]

S5 Table – Detailed report of implementation determinants, with supporting extracted data.

Theme	Information/Data extracted				
	Outcome category	Description	Intervention	Type of study	Reference
1. Intervention/Guideline factors	Barriers	Evidence for effectiveness: Lack of evidence for effectiveness of exercise (non sig PTPT change p = 0.104)	Physical activity counselling and prescription	Quantitative	(64)
1. Intervention/Guideline factors	Barriers	Evidence for effectiveness: Lack of evidence for effectiveness of exercise	Physical activity counselling and prescription	Quantitative	(72)
1. Intervention/Guideline factors	Barriers	Evidence for effectiveness: the results of the CSCs ’ work were so far not of sufficient value to these professionals.	Physical activity counselling and referral	Qualitative	(77)
1. Intervention/Guideline factors	Barriers	HEALTH PROFESSIONALS:<br>Feasibility/compatibility: logistical challenges - busy clinics, long questionnaires, difficulty of managing appointments, difficulty of handling accelerometers.<br><br>Feasibility/compatibility: Consultations are too long sometimes (qual)	Physical activity prescription	Mixed-methods	(50)
1. Intervention/Guideline factors	Barriers	STAKEHOLDERS| Evidence for efectiveness: The lack of confidence in the SPAP method was highlighted as an important barrier to implementation	Physical activity prescription	Qualitative	(62)
1. Intervention/Guideline factors	Barriers	PHC HEALTH PROFESSIONALS| Feasibility/compatibility - Group consultations were not welcomed due to associated complex arrangements (time, space, and logistics).	Physical activity counselling	Qualitative	(67)
1. Intervention/Guideline factors	Barriers	PROFESSIONALS | Evidence for effectiveness: pessimism (i.e. that the intervention is unlikely to help change PA behaviour of patients); beliefs about consequences (i.e. the intervention will only work with a minority of patients).	Physical activity counselling	Qualitative	(78)
1. Intervention/Guideline factors	Barriers	HEALTHCARE PROFESSIONALS | Intervention components/characteristics/content: HCPs indicated that standardizing documentation of patient physical activity levels could make the process confrontational for the patient.<br>Feasibility/compatibility: HCPs emphasized the limited time for each patient and the need to address the primary reason for the visit first.	Physical activity assessment	Qualitative	(79)
1. Intervention/Guideline factors	Barriers	PATIENTS:<br>Intervention components/characteristics/content: social influences (i.e. a preference to complete the programme alone/privately without social support).	Physical activity counselling	Qualitative	(78)
1. Intervention/Guideline factors	Barriers	PATIENTS:<br>Setting: non-participants tend to express that primary care is not necessarily the most appropriate setting for physical activity promotion (internet, television, print media or more physically active friends as their most common and favoured sources of information and norms regarding PA).	Physical activity counselling	Qualitative	(80)
1. Intervention/Guideline factors	Barriers	RESEARCHERS:<br>Devices/technology: Researchers' perception that people had flagged up that they were disappointed with how basic the website is; researchers felt that e-couchER downside with the competition now from all the apps and so many things that are out there that it maybe does look less appealing to patients (qual.)<br>PATIENTS:<br>Devices/technology: aspects of the website intervention were often used only initially and then some people switched to using their phones or other devices to record activity; Many participants felt that e-coachER could offer more in terms of functionality and that it did not provide as much information or as many features as could readily be obtained from other ‘app’-based package; (qual.); not user-friendly IT facilities (qual.)	Physical activity referral scheme	Mixed-methods	(81)
1. Intervention/Guideline factors	Barriers	HCPs:<br>Devices/Technology: Patients—especially older people in rural areas—had difficulties using the technology; It was impractical to ask patients to	Physical activity counselling	Qualitative	(94)

		remember their username and password.			
1. Intervention/Guideline factors	Barriers	REFERRERS:<br>Clarity: lack of understanding of what the scheme actually entails, with the latter being identified as a key barrier to referral.	Physical activity referral scheme	Qualitative	(92)
1. Intervention/Guideline factors	Barriers	PGC PHYSICIANS:<br>Clarity: No specific guidelines (23.9%)	Physical activity counselling	Quantitative	(52)
1. Intervention/Guideline factors	Barriers	PHC PHYSICIANS:<br>Feasibility/compatibility: not enough time (70,6%)	Physical activity counselling	Quantitative	(57)
1. Intervention/Guideline factors	Barriers	NURSES:<br>Feasibility/compatibility: a few nurses questioned the usability of such tools in their routine practice as some patients did not completely understand the user instructions and faced practical and technical problems; most nurses reported that adhering to the consultation structure was more difficult than expected; they thought that using the intervention in routine practice might conflict with other clinical demands during routine consultations.	Physical activity counselling	Qualitative	(58)
1. Intervention/Guideline factors	Barriers	PATIENTS:<br>Intervention components/characteristics/content: The Diary was chore; to do the consultations with the couple.<br><br>Devices/technology: comments about equipment from the participants' perspective was upon the accuracy (or otherwise) of the pedometer;<br><br>PROFESSIONALS:<br>Flexibility/adaptability: given that the trial ran over 12 months, pragmatic adaptations were made by the nurses (in consultation with the team) in response to the specific circumstances of participants, for example, working around holiday periods (e.g. Christmas) or periods of religious observance, such as Ramadan.<br><br>Feasibility/compatibility: they observed that within the time constraints of routine practice, they would not be able to replicate the full intervention as it stood within a routine nurse consultation.	Physical activity counselling	Mixed-methods	(59)
1. Intervention/Guideline factors	Barriers	STAKEHOLDERS:<br>Intervention components/characteristics/content: SPAP is a complex method requiring extensive knowledge on principles for behavioural medicine.<br>Clarity: health professionals perceived that they did not have access to central policy documents that provided enough guidance and direction for undertaking SPAP.<br>Feasibility/compatibility: SPAP method as time-consuming and that they often felt they did not have sufficient time to work according to the method during normal consultations.	Physical activity prescription	Qualitative	(62)
1. Intervention/Guideline factors	Barriers	PHC PROVIDERS:<br>Feasibility/compatibility: don't have enough time to promote PA to their patients (99,7% physicians, 96,4% nurses, 65,4% nurse assistants, 22,6% dietitians and 30,8% health educators Agree or Strongly agree)	Physical activity counselling	Quantitative	(63)
1. Intervention/Guideline factors	Barriers	PHYSICIANS:<br>Feasibility/compatibility: Lack of time (sig PTPT change p = .017).	Physical activity counselling and prescription	Quantitative	(64)
1. Intervention/Guideline factors	Barriers	GPs:<br>Feasibility/compatibility: Insufficient time in consultations	Physical activity counselling	Quantitative	(69)
1. Intervention/Guideline factors	Barriers	PHC PROFESSIONALS:<br>Accessibility of the guideline/recommentation: Lack of guidance/resources in exercise for those with chronic disease.<br>Feasibility/compatibility: Lack of time.	Physical activity counselling and prescription	Quantitative	(72)
1. Intervention/Guideline factors	Barriers	PHC PROFESSIONALS:<br>Intervention components/characteristics/content: Clinicians indicated that the brevity of the instrument was not indicative of the time taken to complete the questionnaire, and inconsequential in deciding their preferences	Physical activity assessment and counselling	Qualitative	(76)
1. Intervention/Guideline factors	Barriers	PROFESSIONALS:<br>Feasibility/compatibility: environmental context and resources (i.e. lack of time to complete training and practice intervention delivery).	Physical activity counselling	Qualitative	(78)
1. Intervention/Guideline factors	Barriers	GPs:<br>Feasibility/compatibility: Time constraints hinder PA counselling due to the large number of patients seen by physicians	Physical activity counselling	Qualitative	(84)
1. Intervention/Guideline factors	Barriers	PHC PROFESSIONALS:<br>Feasibility/compatibility: 60.9% of respondents selected "Not enough time during visit"	Physical activity counselling	Quantitative	(85)

1. Intervention/Guideline factors	Barriers	STAKEHOLDERS:<br>Devices/technology: They were concerned with the cost of the monitors and their accuracy. .Some limitations cited by the stakeholders include: flimsy, bulky, sensitive to measurement, difficult to read, short battery life, and easy to lose	Physical activity counselling	Mixed-methods	(86)
1. Intervention/Guideline factors	Barriers	GPs:<br>Feasibility/compatibility: Time constraints.	Physical activity counselling and referral	Qualitative	(87)
1. Intervention/Guideline factors	Barriers	HEALTH CARE PROFESSIONALS:<br>Feasibility/compatibility: Most noted a lack of time in scheduled appointments as the reason they did not go into detail about exercise or failed to mention it at all.	Physical activity counselling	Qualitative	(88)
1. Intervention/Guideline factors	Barriers	PRACTITIONERS:<br>Protocols: Lack of behaviour change protocols impacted upon their motivation to use behaviour change practices due to a diminished role clarity.	Physical activity referral scheme	Qualitative	(89)
1. Intervention/Guideline factors	Barriers	GPs:<br>Feasibility/compatibility: Lack of time in consultation (medium: 1.49, scale 0-4).	Physical activity counselling	Quantitative	(91)
1. Intervention/Guideline factors	Barriers	REFERRERS:<br>Feasibility/compatibility: the constraints of typical GP practice appointments as a key challenge to referral (difficulty of meeting competing demands; having to balance wider health complaints and the needs of a patient in a short space of time).	Physical activity referral scheme	Qualitative	(92)
1. Intervention/Guideline factors	Barriers	HCPs:<br>Feasibility/compatibility: managing an app is an additional task for providers who deliver PA advice (ie, managing the new technology); Using the application on mobile phones was considered difficult because the providers were distracted by a different electronic device; additional task that required extra time.<br><br>Devices/technology: Technical difficulties - Certain processes were required before providers could start to use PAC app.	Physical activity counselling	Qualitative	(94)
1. Intervention/Guideline factors	Barriers	GPs:<br>Feasibility/compatibility: Lack of time within consultations (other priorities) (61%; quant.) (barrier).	Physical activity counselling and referral	Mixed-methods	(47)
1. Intervention/Guideline factors	Barriers	PHC PROFESSIONALS:<br>Feasibility/compatibility: Time and resource pressures (GPs believed they already have too many responsibilities, it is not always feasible) (barrier)	Physical activity counselling	Qualitative	(48)
1. Intervention/Guideline factors	Barriers	PHC PROFESSIONALS:<br>Feasibility/compatibility: Lack of time in consultations to discuss PA and to seek out information about PA opportunities (barrier);	Physical activity referral scheme	Qualitative	(49)
1. Intervention/Guideline factors	Barriers	PHC PHYSICIANS:<br>Feasibility/compatibility: Time constraint/insufficient time (54.0%)	Physical activity counselling	Quantitative	(52)
1. Intervention/Guideline factors	Barriers	CARE SPORTS CONNECTORS| Feasibility/compatibility: PHC professionals lack of time; sport clubs collaborators lack of time.	Physical activity referral scheme	Qualitative	(60)
1. Intervention/Guideline factors	Barriers	GPs:<br>Setting: PA promotion should be done outside of the health-care centers, because the patient is ill when she/he visits PHC.<br>Feasibility/compatibility: Lack of time - elevated patients' ratio attended by physicians daily.<br><br>NURSES:<br>Feasibility/compatibility: Lack of time in consultation.	Physical activity prescription	Qualitative	(95)
1. Intervention/Guideline factors	Barriers	Feasibility or compatibility: GPs: more than half of the interviewees referred to the fact that everyday office routine would often not allowthem to "manage a full, long-term consultations" (I-38 m). This would be particularly challenging in case of older people, as more specifics (e.g. previous diseases, compatibility with ongoing therapies) must be taken into consideration and the need for care would be greater; lack of time.	Physical activity counselling	Qualitative	(96)
1. Intervention/Guideline factors	Barriers	EXERCISE PRACTITIONER:<br>Feasibility/compatibility: GPs have only 10 minutes to complete the patient referral form (on top of all the other clinical stuff that is likely more urgent) - We therefore can't be expecting a lot of baseline information to be provided at the GP level.	Physical activity referral scheme	Qualitative	(97)
1. Intervention/Guideline factors	Barriers	PATIENTS:<br>Tailored/patients centred: "[Program] is against providing individualized care based on needs"	Physical activity prescription	Qualitative	(99)
1. Intervention/Guideline factors	Barriers	QUANTITATIVE PHASE:<br>Feasibility/compatibility: Inadequate consultation time (22% GPs; 46% EPs; 11% total).	Physical activity referral scheme	Mixed-methods	(103)

1. Intervention/Guideline factors	Facilitators	Tailored intervention/patient-centred: Tailored exercise to meet individual patient needs; general exercise better than local exercise	Physical activity counselling	Quantitative	(69)
1. Intervention/Guideline factors	Facilitators	Evidence for effectiveness: Showing results and successes can lead to more involvement by professionals and organizations.	Physical activity counselling and referral	Qualitative	(77)
1. Intervention/Guideline factors	Facilitators	Tailored intervention/patient-centred: tailor referral to patients' characteristics (qual.) (facilitator).	Physical activity counselling and referral	Mixed-methods	(47)
1. Intervention/Guideline factors	Facilitators	Evidence for effectiveness: H14 need to evaluate effectiveness also in a qualitative manner (benefits that the client will experience and communicate them to health professionals); essential to collect evaluation data from different stakeholders	Physical activity counselling and referral	Mixed-methods	(51)
1. Intervention/Guideline factors	Facilitators	PROFESSIONALS | Recruitment strategy - a wide ranging and long-term promotional strategy is essential for successful recruitment (not only at the beginning of the project) (qual); project recruitment increase following GP surgeries mailed-out all patients fulfilling inclusion criteria (quant.).	Physical activity referral scheme	Mixed-methods	(56)
1. Intervention/Guideline factors	Facilitators	NURSES| Intervention components/characteristics/content: Most nurses found that goal setting and action planning enabled them to stimulate patients in formulating their goals and actions, which in turn facilitated patients' goal attainment; The use of the activity log to review patients' level of goal attainment facilitated them in giving feedback on their behaviour nurses were convinced that the combination of the accelerometer, activity log and their subsequent and structural support incentivised patients' goal attainment in changing their physical activity, which strengthened their positive beliefs about the feasibility of the intervention in their routine practice; The use of self-monitoring tools such as the accelerometer and activity log were seen as additional motivators and incentives for patients, as they provided insight into patients' level of physical activity and challenged patients to goal attainment.<br><br>Evidence for effectiveness: Nurses' beliefs about the use of the intervention in their routine practice strongly depended on their beliefs about the effectiveness of the intervention to increase patients' level of physical activity and health outcomes. Nurses were convinced that the effectiveness of the intervention relied on patients' engagement to set goals and having a reasonable level of health literacy to understand the intervention materials.<br><br>Feasibility/compatibility: The nurses believed that the use of such tools would help them to deliver the intervention in their routine practice; Nurses needed more time to deliver the intervention, which may adversely influence the feasibility due to time constraints in their routine practice.	Physical activity counselling	Qualitative	(58)
1. Intervention/Guideline factors	Facilitators	PHC HEALTH PROFESSIONALS | Intervention components/characteristics/content: provision of the available social (family) support; Consultations were the most desirable intervention.<br>Tailored intervention/patient-centred: provision of personalized PA interventions to patients.	Physical activity counselling	Qualitative	(67)
1. Intervention/Guideline factors	Facilitators	GPs | Intervention components/characteristics/content: follow-up; 413 (57%) stated they would promote exercise by referring the patient to a physiotherapist	Physical activity counselling	Quantitative	(69)
1. Intervention/Guideline factors	Facilitators	PHYSIOTHERAPISTS | Recruitment strategy: recruitment pathway should be open to all patients attending physiotherapy; recruitment pathway should be incorporated into other preventative or chronic disease programmes.<br>Intervention components/characteristics/content: May be appropriate to follow up some patients more frequently than 3 and 6 months; Coaching of patients who relapse should be dependent on their motivation to change.	Physical activity counselling and referral	Mixed-methods	(70)
1. Intervention/Guideline factors	Facilitators	RESEARCHERS:<br>Recruitment strategy: Researchers suggested that a more direct approach to recruitment would improve rates (e.g. more refined criteria); researchers suggested that simplifying the referral and paperwork, streamlining the referral system, contacting participants via the ERS rather than via the GP, or contacting the participants directly about the study after the ERS practitioner had sought permission to pass on contact details to the researcher were found to be helpful (qual.)	Physical activity referral scheme	Mixed-methods	(81)
1. Intervention/Guideline factors	Facilitators	GPs | Intervention components/characteristics/content: GPs counselled patients to participate in PA according to the FITT mnemonic however, the intensity of PA was rarely discussed with patients; Healthcare providers including nurses, physiotherapists and other exercise specialists could	Physical activity counselling	Qualitative	(84)

		support physicians in PA counselling			
1. Intervention/Guideline factors	Facilitators	PATIENTS:<br>Intervention components/characteristics/content:<br>HPs connecting patients to tangible options is favourable because they perceive it as helping them towards implementing the changes instead of just being told 'you should get more active'.	Physical activity referral scheme	Qualitative	(49)
1. Intervention/Guideline factors	Facilitators	PATIENTS | Intervention components/characteristics/content: Add diet advice to PA intervention	Physical activity prescription	Mixed-methods	(50)
1. Intervention/Guideline factors	Facilitators	PATIENTS | Evidence for effectiveness: to receive a detailed description of the PAP concept, and to be informed about possible health effects.<br><br>Tailored intervention/patient centred: individualized PAP.<br><br>Intervention components/characteristics/content: crucial to have a clear plan for when, where, and how the PA should start and continue (agreement or a duty that they wanted to uphold); have a written prescription, for less motivated patients (more motivated, taken more seriously, good to show when entering the gym)<br>regular follow-ups to maintain motivation because the follow-ups provided a visualisation of the effects of metabolic riskfactors on health; participants appreciated and desired more follow-ups (extra motivating factor and opportunity for ongoing support; high degree of perceived safety); social support (family, friends, group PA sessions); participants make their own choices of PA with the support of the health care provider.	Physical activity prescription	Qualitative	(54)
1. Intervention/Guideline factors	Facilitators	PATIENTS:<br>Intervention components/characteristics/content: social engagement with members of the local community with similar health profiles (qual)<br><br>Tailored intervention/patient centred: appropriate tailored physical activity (qual).<br><br>PROFESSIONALS:<br>Tailored intervention/patient-centred: appropriate tailored physical activity (qual).	Physical activity referral scheme	Mixed-methods	(56)
1. Intervention/Guideline factors	Facilitators	PATIENTS:<br>Intervention components/characteristics/content: Patients agreed that the process needs to be more than just a series of questions; patients indicated they would be receptive to discussing physical activity, although they acknowledged that not everyone would feel this way.	Physical activity assessment	Qualitative	(79)
1. Intervention/Guideline factors	Facilitators	PATIENTS:<br>Tailored intervention/patient-centred: intervention content would need to be sensitively tailored to the physical limitations patients faced.<br><br>Recruitment strategy: shortened and more concise invitation letters, with potential benefits of participation clearly highlighted and outlining the exact time requirements of participation (recruitment); initial contact by email or text messaging would be preferable (recruitment); to overcome time constraints, extending recruitment deadlines or offering more flexible appointment times or settings.	Physical activity counselling	Qualitative	(80)
1. Intervention/Guideline factors	Facilitators	PATIENTS:<br>Devices/technology: user-friendly IT facilities; lack of support from the ERS and that e-coachER was able to mitigate the effects of this (qual.); other participants found that, if their health condition had a negative impact on their motivation or ability to exercise, e-coachER provided reassurance and fostered their sense of competence (if they haven't achieve everything, that's ok - easy-to-understand, flexible and supportive resource); reminder utility; goal-setting aspects of the e-coachER support package, particularly pedometer and setting step-count goals, were particularly important components of the package; e-coachER as a prompt to the ERS; being able to choose their preferred level of engagement with e-coachER was a valuable feature and allowed participants autonomy over their exercise choices; e-coachER package was instrumental in helping to ameliorate any feelings of guilt experienced when participants perceived their efforts to increase PA were in some way unsatisfactory; e-coachER was therefore an important alternative or seen as separate to ERS (qual.)<br><br>Intervention components/characteristics/content: desire to avoid not meeting these goals was also motivating and participants would set their goals to avoid feelings of failureand/or guilt, with one participant using paper-based goal-setting to avoid setting goals online with e-coachER;participant fund that just having the interview was 'motivational'; social support (including from research staff) (qual.)	Physical activity referral scheme	Mixed-methods	(81)
1. Intervention/Guideline factors	Facilitators	HCPs:<br>Devices/technology: The application could involve the patient in two different ways - the patient could use the application without any support from providers (see the results generated by the application or discuss them with providers); the patient could receive information and counselling from	Physical activity counselling	Qualitative	(94)

		providers based on results provided by the application; The app had the potencial to synthesize patient information and offer individualized advice and was thus able to facilitate personalized PA counselling; helps to assess information and provides data.			
1. Intervention/Guideline factors	Facilitators	PHC PROFESSIONALS:<br>Recruitment strategy: an intervention that PCPs could signpost to, but ultimately based in the community where patients self-refer themselves (facilitator)	Physical activity counselling	Qualitative	(48)
1. Intervention/Guideline factors	Facilitators	GPs:<br>Intervention components/chraracteristics/content: GPs identified the "patient to link-worker"-setup to potentially facilitate the process and relieve the pressure on general practice (the rationale behind<br>the link-worker role is that without support, navigating and accessing PAs in the community can be extremely challenging for some groups of people) - link-worker<br>was considered a key component of successful Social Prescribing for vulnerable patients;<br>potential facilitator of successful Social Prescribing that the PAs take place<br>outside the healthcare setting (incorporate PA as a natural and permanent part of daily life, it should be experienced as a pleasant way to spend one's leisure time rather than a temporary medical treatment)	Physical activity counselling and referral	Qualitative	(87)
1. Intervention/Guideline factors	Facilitators	HEALTH CARE PROFESSIONALS:<br>Feasibility/compatibility: nurse practitioners might have more time to address the issue; patients should be referred to other providers for exercise counseling.	Physical activity counselling	Qualitative	(88)
1. Intervention/Guideline factors	Facilitators	REFERRERS:<br>Recruitment strategy: patients could self-refer directly to the scheme and by passsign-off from a health professional (desirable time saving approach for referral)	Physical referral scheme	Qualitative	(92)
1. Intervention/Guideline factors	Facilitators	PATIENTS | Devices/technology: The activity tracker was used by all participants; Those who appreciated the SMS reminders found them inspiring and helpful (facilitators).	Physical activity counselling	Qualitative	(43)
1. Intervention/Guideline factors	Facilitators	PHC PROFESSIONALS:<br>Accessibility of the guideline/recommendation: promoting accessible and reliable online sources and encouraging PCPs to provide a brief intervention opportunistically (facilitator)	Physical activity counselling	Qualitative	(48)
1. Intervention/Guideline factors	Facilitators	HEALTH PROFESSIONALS:<br>Devices/technology: want to keep pedometers (project sustainability)	Physical activity prescription	Mixed-methods	(50)
1. Intervention/Guideline factors	Facilitators	PATIENTS<br>Intervention components/characteristics/content: There were fewer comments about the diary, which was seen as being motivating<br>PROFESSIONALS<br>Intervention components/characteristics/content: to do the consultations with the couple (social support)	Physical activity counselling	Mixed-methods	(59)
1. Intervention/Guideline factors	Facilitators	PHC PROFESSIONALS:<br>Intervention components/characteristics/content: The questionnaires prompted patients to think about their activity; Clinicians referred to using the instrument as a mechanism for starting a conversation with the patient about PA, rather than raising with topic independently.	Physical activity assessment and counselling	Qualitative	(76)
1. Intervention/Guideline factors	Facilitators	PRACTITIONERS:<br>Protocols: behaviour change protocols including practice manuals, service plans, and resources for behaviour change interactions were important for implementation.	Physical activity referral scheme	Qualitative	(89)
1. Intervention/Guideline factors	Facilitators	PATIENTS:<br>Devices/technology: Valued the Physical Activity (PA) tracker to provide an accountable measure of progress.	Physical activity counselling	Mixed-methods	(90)
1. Intervention/Guideline factors	Facilitators	DIETITIANS:<br>Feasibility/compatibility: consultation time/time with patients (e.g. dietitians appointments were typically 30–60 min, whereas physician appointments were shorter, such as10–15 min) (facilitator).	Physical activity counselling	Qualitative	(46)
1. Intervention/Guideline factors	Facilitators	GPs:<br>Protocols: Use of common protocol for PA promotion and prescription.	Physical activity prescription	Qualitative	(95)
1. Intervention/Guideline factors	Facilitators	GPs:<br>Content: GPs: sensitisation plays a central role, particularly in case of patients with pre-existing diseases; you can become active as a coach yourself, by agreeing with the patient on specific targets, but this requires time reserves and follow-up procedures; GPs' advice is taken seriously by most patients and submitted recommendations are accepted; of central importance not just to appeal to the patient to do exercises, but rather encourage an "intrinsic motivation" in him or her, which can be "internalised quickly in the sense of a natural daily or weekly rhythm".	Physical activity counselling	Qualitative	(96)

		Tailored/parient centred: advocated that upon suggesting certain activities the general practitioner shall anticipate “how well the chosen activity would be compatible with the patient’s personality” (I-40 m) and how good it could be “integrated into the patient’s everyday life and living conditions”.<br>Devices: potentials of health apps were seen particularly in the areas of motivation, information and lifestyle change, as well as the playful element which was considered to be highly useful in promoting exercise and maintaining health among older people.			
1. Intervention/Guideline factors	Facilitators	EXERCISE PRACTITIONER:<br>Intervention components/content/characteristics: With the correct (funding and) procedures in place, it can be done.<br><br>FITNESS CENTRE AREA MANAGER:<br>Flexibility/adaptability: allowing us to adapt and make changes to the intervention - We were making the changes that were driven by real-world problems, not problems we just came up with and this helped implement improvements to facilitate intervention adherence.<br><br>PUBLIC HEALTH COMMISSIONER AND GP:<br>Flexibility/adaptability: "can we challenge the current rules that are in place for the system. If not, fine let’s adapt – if we can challenge the status quo, let’s innovate!".<br>Clarity: "at the very beginning, we need to make clear everyone understands ‘what there is now’ and ‘this is what control or power we have’ before we go trying to adapt or create anything".<br><br>ACADEMIC:<br>Evidence for effectiveness: importance of research having an impact on practice, and with it the need to move beyond traditional positivist approaches - importance of working with those on the ground who will be delivering and receiving services, and how evidence-based approaches can be embedded in practice within a relatively short timescale (when compared with the commonly estimated 17 years for research to reach practice) (Morris, Wooding, and Grant 2011); Unlike more traditional researcher-led interventions, delivery was not dependent on the research team. When the research ended, the co-produced intervention continued to operate; maintaining the co-production throughout the entire project phases; research and practice need to go hand in hand, and stakeholder involvement lies at the heart of successful intervention design.	Physical activity referral scheme	Qualitative	(97)
1. Intervention/Guideline factors	Facilitators	PATIENTS:<br>Intervention components/characteristics/content: “no change was needed. [The patient] was very happy the way it is.”	Physical activity prescription	Qualitative	(99)
1. Intervention/Guideline factors	Facilitators	QUANTITATIVE PHASE:<br>Evidence for efectiveness: Patient-reported improved health outcome (improved health condition due to PA programme) (75% GPs; 90% Eps; 42% total); Presence of objectively measured outcome (The health gains can be measured) (53% GPs; 74% EPs; 32% total).<br>Feasibility/compatibility: Reduces the work burden placed on doctors/GPs (44% GPs; 60% EPs; 26% total).<br>Accessibility: Easily accessible or ease ofuse of PARS (46% GPs; 41% EPs; 13% total).<br><br>QUALITATIVE PHASE:<br>Evidence for effectiveness: Both GPs and EPs indicated that PA referral schemes had enhanced patients’ health outcomes.	Physical activity referral scheme	Mixed-methods	(103)
2. Individual health professional factors	Barriers	GENERAL PRACTITIONERS | Cognitions/attitudes: GP not convinced about PAP interest (mean=1,91 - score from 1 to 10) (is not an important barrier);<br>Knowledge and skills: Physician do not know where to refer (mean=6,01 - score from 1 to 10) (barrier); Physician’s lack of training (mean=5,46 - from 1 to 10) (barrier).	Physical activity prescription	Quantitative	(44)
2. Individual health professional factors	Barriers	PHC HEALTH PROFESSIONALS| Knowledge and skills: poor knowledge about PA couselling (24% responses) (barrier)	Physical activity counselling	Quantitative	(45)
2. Individual health professional factors	Barriers	DIETITIANS| Scope of practice/professional role: feeling that it is outside the scope of practice (barrier).<br>Knowledge and skills: lack of expertise, which included lacking knowledge, skills, and/or training to go into more depth with PA counselling (barrier)	Physical activity counselling	Qualitative	(46)
2. Individual health professional factors	Barriers	GENERAL PRACTITIONERS | Cognitions/attitudes: Lack of belief in effectiveness (2%; quant.); Personally don’t believe exercise is an effective treatment (0%; quant.); not always relevant (n=1; quant.) (barriers).<br>Motivation: lack of motivation (39%; quant.)<br>Scope of practice/professional role: Role for non-clinical support workers (n=1; quant.)(barrier); belief that PA promotion is a responsibiity of all heathcare providers, the work is always being dumped on the GP that has less time (qual.) (barrier).	Physical activity counselling and referral	Mixed-methods	(47)

2. Individual health professional factors	Barriers	PHC PROFESSIONALS| Cognitions/attitudes: Community Pharmacists reported not feeling comfortable with opportunistically providing health promotion (barrier); Fear of offending was a barrier preventing participants providing opportunistic guidance, in which weight and image were deemed sensitive topics - difficulty in identifying preconception patients (barrier); consider self- management options as having greater potential (barrier).<br><br>Knowledge and skills: Unaware of any guidelines on preconception care and PA; no search for guidelines as they think they are transferable from general population guidelines (barriers); Not skilled enough - Belief that guidance on PA may not be as effective as other sources of information (e.g personal trainers, physiotherapists)	Physical activity counselling	Qualitative	(48)
2. Individual health professional factors	Barriers	PHC PROFESSIONALS:<br>Cognitions/attitudes: Health professional-patient interaction, perceived by the health professional (barrier or facilitator).<br><br>Scope of practice/professional role: perception of self professional role/identity - should not be solely responsible for this task (barrier)<br><br>Knowledge and skills: Lack of knowledge about available PA opportunities (barrier); do not know the appropriateness and danger-free character of the PA opportunities available (barrier).	Physical activity referral scheme	Qualitative	(49)
2. Individual health professional factors	Barriers	HEALTH PROFESSIONALS:<br>Knowledge and skills: half reported to require more information on the PA behaviour change techniques (BCT); need information on the available PA facilities in the nearby community (qual.)	Physical activity prescription	Mixed-methods	(50)
2. Individual health professional factors	Barriers	HEALTH PROFESSIONALS:<br>Cognitions/attitudes: personal attitudes (e.g. do not believe in the possibilities of PA) avoided bringing up PA with the client.<br><br>Knowledge and skills: health professionals do not provide enough information to patients about the counselling and referral program and/or patients feel forced to the program (social skills of the health professional).	Physical activity counselling and referral	Mixed-methods	(51)
2. Individual health professional factors	Barriers	PHC PHYSICIANS| Professional profile: Family medicine physicians significantly counseled more patients, more frequently, and had an increased desire to counsel more patients than internists and general practitioners (P = 0.002)<br><br>Knowledge and skills: Lack of knowledge (26.1%); Lack of evidence (11.9%)	Physical activity counselling	Quantitative	(52)
2. Individual health professional factors	Barriers	PHC PHYSICIANS| Cognitions/attitudes: low priority to exercise as a treatment.<br><br>Scope of practice/professional role: Prescribing exercise is perceived as being outside of most PCPs' scope of practice.<br><br>Knowledge and skills: PCPs are not certain that exercise really works as a medical treatment; limited knowledge of exercise prescription; lack of training in exercise prescription	Physical activity prescription	Qualitative	(55)
2. Individual health professional factors	Barriers	PROFESSIONALS:<br>Cognitions / attitudes: physical activity was not always seen as a priority prevention strategy by health professionals (qual);	Physical activity referral scheme	Mixed-methods	(56)
2. Individual health professional factors	Barriers	PHC PHYSICIANS| <br>Knowledge and skills: Not adequately trained in the area (38,09%); too difficult to evaluate and manage (5,44%).<br><br>Professional profile: Physicians who had graduated from domestic universities were less likely to provide general counselling (OR,0.39;95%CI,0.14–1.13;p = 0.08), written prescription (OR,0.34;95%CI,0.09–0.1.33;p = 0.12), or systematically track/follow-up PA in patients without chronic disease (OR,0.33;95%CI,0.11–0.99;p = 0.05), than were physicians who had graduated from international universities - this difference was not statistically significant for patients with chronic disease; graduates from domestic universities were statistically less likely to promote PA to adult patients (OR, 0.12; 95%CI, 0.017–0.80; p =0.03) and less likely to promote PA to pediatric patients (OR, 0.62; 95% CI, 0.23–167; p=0.13) than were those who had attended international universities.<br><br>Cognitions / attitudes: fear of offending the patient (2,7%).	Physical activity counselling	Quantitative	(57)

		Scope of practice/professional role: not part of my role (3,4%).<br><br>Sociodemographic characterisctics: Physicians aged 31yrs or more were less likely to assess and promote PA in pregnant women (OR:0.41, 95% CI 0.15–1.13, p = 0.08),(OR:0.18,95%CI0.04–0.86,p = 0.03), respectively, and less likely to refer patients without chronic disease for further evaluation and management for PA (OR:0.44, 95% CI 0.21–0.93, p = 0.03); geographic location.<br><br>Professional behaviour: physicians who saw fewer adult patients (20 adult patients per day or less) were less likely to provide general counseling to patients without chronic disease (OR, 0.33; 95%CI, 0.15–0.72; p=0.005).			
2. Individual health professional factors	Barriers	NURSES| Cognitions / attitudes: Nurses' beliefs about the use of the intervention in their routine practice strongly depended on their beliefs about the effectiveness of the intervention to increase patients' level of physical activity and health outcomes. Nurses were convinced that the effectiveness of the intervention relied on patients' engagement to set goals and having a reasonable level of health literacy to understand the intervention materials.<br><br>Knowledge and skills: PCPs are not certais that exercise really works as a medical treatment; limited knowledge of exercise prescription; lack of training in exercise prescription	Physical activity counselling	Qualitative	(58)
2. Individual health professional factors	Barriers	CARE SPORTS CONNECTORS| Knowledge and skills: PHC professionals lack of knowledge of suitable PA activities; sport clubs collaborators lack of knowledge and experience of working with the target group;<br><br>Professional behaviour: GP referral towards a physiotherapist was easier; some physiotherapists had their own exercise lessons for their patients and preferred to refer their patients towards their own sport offer instead of to a Care Sport Connector.<br><br>Expectations: not to have high expectations of the sport clubs because the volunteers lacked time and knowledge.	Physical activity referral scheme	Qualitative	(60)
2. Individual health professional factors	Barriers	STAKEHOLDERS| Knowledge and Skills: need for increased knowledge on how to talk about health behaviours in patient consultations; need for increase knowledge and belief in the SPAP method; SPAP was beyond their competence, specifically where patients with health disorders including comorbidity were concerned.	Physical activity prescription	Qualitative	(62)
2. Individual health professional factors	Barriers	PHYSICIANS| Knowledge and skills: Personal knowledge (p = .026); Lack of exercise education in medical school (sig PTPT change p = .001)<br>Cognitions/attitudes: Other lifestyle changes more important (sig PTPT change p = .006)	Physical activity counselling and prescription	Quantitative	(64)
2. Individual health professional factors	Barriers	STAKEHOLDERS|Cognitions / attitudes: Exercise Professionals suggested that primary care health professionals did not take ERS seriously as a treatment option (lack of engagement, limited involvement after their referral, etc.).	Physical activity referral scheme	Qualitative	(65)
2. Individual health professional factors	Barriers	PHC HEALTH PROFESSIONALS| Knowledge and skills: lack of knowledge and skills in patients with a chronic condition and/or with multiple comorbidities.<br><br>Scope of practice/professional role: no consensus on who should take the responsibility of PA for adults with diabetes in PHC (dieticians did not feel that it could be their responsibility, however, other HPs thought that PA responsibilities should be allocated to the dieticians).	Physical activity counselling	Qualitative	(67)
2. Individual health professional factors	Barriers	FAMILY PHYSICIANS| Cognitions / attitudes: Lack of conviction about the effectiveness of simply write an exercise prescription, unless they could also provide patients with behavioural support; it required more time to properly explain a written physical activity prescription when compared with explaining other types of prescriptions like medication.<br><br>Professional behaviour: PA prescriptions are not part of their routine.	Physical activity prescription	Qualitative	(68)
2. Individual health professional factors	Barriers	GENERAL PRACTITIONERS|Knowledge and skills: insufficient expertise, uncertainty about the most appropriate type of exercise; uncertainty about the effects of exercise; uncertainty about the safety of exercise	Physical activity counselling	Quantitative	(69)
2. Individual health professional factors	Barriers	NURSES| Cognitions / attitudes: Nurses' own belief of the required effort was used to explain low patient take-up of exercise; discussing exercise goals is not prioritised because within the primary care setting, the pursuit of changing other be-haviours (e.g. diet) is perceived as more realistic.	Physical activity counselling	Qualitative	(71)

		Knowledge and skills: nurces' felt lack of motivational interviewing skills.			
2. Individual health professional factors	Barriers	PHC PROFESSIONALS:<br>Knowledge and skills: Lack of exercise education in medical school; lack of knowledge.<br>Cognitions/attitudes: Other lifestyle changes more important	Physical activity counselling and prescription	Quantitative	(72)
2. Individual health professional factors	Barriers	HEALTH PROFESSIONALS:<br>Knowledge and skills: 6,3% of Community Health Workers reported being unprepared or not trained enough to speak with their patients about maintaining exercise.	Physical activity counselling	Quantitative	(74)
2. Individual health professional factors	Barriers	FAMILY PHYSICIANS:<br>Knowledge and skills: period of adaptation and heightened understanding of the concepts of PA assessment.For less knowledgeable clinicians, the time taken to complete an assessment is likely to be longer, particularly if the instrument does not support limited knowledge/competenc	Physical activity assessment and counselling	Qualitative	(76)
2. Individual health professional factors	Barriers	HEALTH PROFESSIONALS:<br>Knowledge and skills: knowledge about the PA offer, unfamiliarity with the CSC role.<br><br>Cognitions / attitudes: own interest; professionals perceived the target group themselves as a barrier.	Physical activity counselling and referral	Qualitative	(77)
2. Individual health professional factors	Barriers	PROFESSIONALS:<br>Knowledge and skills: memory, attention and decision processes (i.e. difficulties recalling specific components of the intervention).<br><br>Scope of practice/professional role: professional role and identity (i.e. belief that nurses and not GPs should be delivering the intervention).	Physical activity counselling	Qualitative	(78)
2. Individual health professional factors	Barriers	HEALTHCARE PROFESSIONALS:<br>Knowledge and skills: HCPs feel they lack sufficient knowledge about what to recommend after asking about physical activity; Many HCPs are unfamiliar with physical activity guidelines or local programs for referrals, and are not comfortable with tailoring recommendations for chronic conditions.	Physical activity assessment	Qualitative	(79)
2. Individual health professional factors	Barriers	PHYSICIANS:<br>Scope of practice/professional role: believing other professionals should provide PA counselling.	Physical activity counselling	Quantitative	(82)
2. Individual health professional factors	Barriers	GENERAL PRACTITIONERS: Knowledge and skills: Physicians had insufficient knowledge regarding general<br>PA guidelines as well as those for people with diseases. Moreover, communication skills for PA counselling were also insufficient. Physicians could not follow PA patterns of many patients.	Physical activity counselling	Qualitative	(84)
2. Individual health professional factors	Barriers	PHC PHYSICIANS:<br>Cognitions / attitudes: In terms of attitude and belief barriers, 10.0% selected “I don't think counseling is effective,”.<br>Knowledge and skills: 4.2% selected “I don't know what to recommend.”	Physical activity counselling	Quantitative	(85)
2. Individual health professional factors	Barriers	GPs:<br>Cognitions / attitudes: During the consultation there are typically other things which are more important.	Physical activity counselling and referral	Qualitative	(87)
2. Individual health professional factors	Barriers	GPs:<br>Cognitions / attitudes: Not a reason for a consultation (medium: 1.21; scale 0-4)	Physical activity counselling	Quantitative	(91)
2. Individual health professional factors	Barriers	SCHEME DELIVERERS:<br>Cognitions / attitudes: Recognising the value of the scheme as a worthwhile ex-perience for patients was also discussed as a determinantof referral.<br><br>Health profile: Referrers with a higher BMI were considered less likely to refer due to a perceived fear of judgement.	Physical activity referral scheme	Qualitative	(92)
2. Individual health professional factors	Barriers	PATIENTS:<br>Professional behaviour: HP manner and approach to topic in a way that do not make patients feel guilty because they are not doing enough physical activity.	Physical activity referral scheme	Qualitative	(49)
2. Individual health professional factors	Barriers	POTENTIAL CLIENTS:<br>Health profile: Afraid that the counsellor may be “too athletic”.<br>Professional behaviour: afraid that the counsellor does not understand the clients’ restrictions regarding PA.	Physical activity counselling and referral	Mixed-methods	(51)
2. Individual health professional factors	Barriers	PHC PROFESSIONALS:<br>Scope of practice/professional role: GPs and community pharmacists do not believe they should be solely responsible for delivering this information and would prefer to signpost patients to the appropriate sources (barrier).	Physical activity counselling	Qualitative	(48)
2. Individual health professional	Barriers	PROFESSIONALS |	Physical activity	Qualitative	(78)

factors		Established professional habits: social influences (i.e. improving diabetes care is considered to be a practice norm and something healthcare professionals are already doing);	counselling		
2. Individual health professional factors	Barriers	GPs and NURSES:<br>Knowledge: Self-perception in the lack of knowledge for exercise prescription.<br><br>NURSES:<br>Scope of practice/professional role: the leading role in prescription is not clear.<br>Knowledge/skills: Lack of academic training in some PHC professionals about PAP as a preventive and rehabilitation resource.<br><br>GPs:<br>Knowledge/skills: Lack of awareness for any health professionals regarding PAP as a preventive and rehabilitation resource; Lack of knowledge about external PA resources.	Physical activity prescription	Qualitative	(95)
2. Individual health professional factors	Barriers	GPs:<br>Lack of knowledge: “did not feel fully knowledgeable” about exercise counselling and prescriptions.<br>Scope of practice / professional role: Some GPs also did not consider themselves as primarily responsible for prescribing physical activity.	Physical activity counselling	Qualitative	(96)
2. Individual health professional factors	Barriers	QUANTITATIVE PHASE:<br>Lack of knowledge/skills: Lack of knowledge on referral pathways (36% GPs; 79% Eps; 19% total).	Physical activity referral scheme	Mixed-methods	(103)
2. Individual health professional factors	Facilitators	PHC PATIENTS | Professional behaviour: Trustworthy and non-judgemental advice by the diabetes nurse, complementing the activity tracker (facilitator).	Physical activity counselling	Qualitative	(43)
2. Individual health professional factors	Facilitators	GENERAL PRACTITIONERS | Health profile: GPs prescribing PA had a significantly better PA level (OR = 2.83, CI95[1.28; 7.00], p = 0.0151) (facilitator).<br><br>Motivation: A high motivation score was a significant determinant in the profile of effective prescribers (OR = 2.57, CI95[1.10; 6.11], p = 0.03) (facilitator); physician-related barriers decreased with a high motivation score (p=0,03) (facilitator).<br><br>Knowledge and skills: Additional training about adapted PA prescription (mean=4,72 - from 1 to 10) (facilitator)	Physical activity prescription	Quantitative	(44)
2. Individual health professional factors	Facilitators	PHC HEALTH PROFESSIONALS| Cognitions/attitudes: perception of no barriers to PA counselling (OR=3,49; CI: 2,17-5,62); higher self-efficacy for counselling (OR=1,79; CI: 1,18-2,68); higher attitude towards PA couselling (OR=1,69; CI: 1,11-2,45) (facilitators).<br><br>Health profile: positive self-rated health (OR=2,02; CI:1,33-3,08).	Physical activity counselling	Quantitative	(45)
2. Individual health professional factors	Facilitators	GENERAL PRACTITIONERS | Knowledge and skills: Knowledge of effectiveness (14%; quant.); atending local information events about the local referral scheme encourages to refer more (qual.) (facilitators)	Physical activity counselling and referral	Mixed-methods	(47)
2. Individual health professional factors	Facilitators	PHC PROFESSIONALS:<br>Cognitions/attitudes: Health professional-patient interaction, perceived by the health professional (barrier or facilitator); perceived self position of influence (facilitator).	Physical activity referral scheme	Qualitative	(49)
2. Individual health professional factors	Facilitators	HEALTH PROFESSIONALS:<br>Knowledge and skills: need to train everyone in the health center (qual.)	Physical activity prescription	Mixed-methods	(50)
2. Individual health professional factors	Facilitators	HEALTH PROFESSIONALS<br>Knowledge and skills: health care professionals with the skills to select those clients who have the opportunities to benefit from a physical activity counselling process; having good background information about the client; personnal experience on how the conselling and referral process works makes easier the use of counselling referrals in general; good social skills of the PA cousellors (e.g penness, friendliness, enthusiasm, patience, reliability, ability to motivate and a positive attitude).<br><br>Professional behaviour: enthusiastic health professionals; give some time to patients after te referral and before the first appointment (opportunity for them to think about their PA makes them more motivated)<br><br>POTENTIAL CLIENTS:	Physical activity counselling and referral	Mixed-methods	(51)

		Professional behaviours: Good social skills of the PA cousellor (e.g. attitude, personality and genuine interest are fundamental, professional knowledge is not enough); understandable short-term goals.			
2. Individual health professional factors	Facilitators	FAMILY MEDICINE PHYSICIANS| Knowledge and skills: There was statistically significant difference between prescribing exercise frequency and learning at medical faculty and Family Medicine specialty training (Z=-2,25 p=0.024, and Z=-3,67 p<0.001, respectively) - exercise prescription frequency of physicians who learned at medical faculty or specialty training is in higher rate.<br><br>Health profile: significant correlation between the participants' own exercising and prescribing exercise frequencies (r=0.169, p=0.042).	Physical activity prescription	Quantitative	(53)
2. Individual health professional factors	Facilitators	PHC PHYSICIANS| Professional profile: family physicians were more likely to assess and promote PA to adult patients (p<0.05) compared to the general physicians; Family physicians differed significantly from other specialists in the (higher) frequency of providing general counseling and verbal behavioral counseling as well as in following up and tracking patients with chronic diseases; physicians who graduated from domestic universities were more likely to assess (p= 0.01) and promote PA (p=0.002) among pregnant women than physicians who graduated from international universities; physicians who saw less patients per day were more likely to systematically track/follow-up on the PA of patients (OR, 3.13; 95% CI, 1.14–8.58; p = 0.03) and to promote PA to pediatric patients (OR, 2.87; 95% CI, 1.37–6.00; p = 0.005).<br><br>Knowledge and skills: Physicians who received training in medical school or followed a PA counseling specialty program were more likely to assess PA in adults than those who had not received such training (p= 0.04); Physicians with good or excellent level of knowledge of PA guidelines and recommendations were more likely to promote PA in pediatric patients, and those with excellent level of knowledge were more likely to assess PA in pregnant women (OR,5.16;95%CI,0.68–39.36).<br><br>Health profile: physicians meeting the recommendation of 7 or more hours of sleep per night, they were more likely to assess PA in pregnant women (OR, 4.42; 95% CI, 1.12–17.35; p=0.03).<br><br>Sociodemographic characteristics: geographic location.	Physical activity counselling	Quantitative	(57)
2. Individual health professional factors	Facilitators	NURSES| Knowledge and skills: the importance of acquiring knowledge and skills.	Physical activity counselling	Qualitative	(58)
2. Individual health professional factors	Facilitators	NURSES<br>Knowledge and skills: BCTs training welcomed and improved their practice during the trial, but also provided them with enhanced skills to take into practice beyond and after the trial	Physical activity counselling	Mixed-methods	(59)
2. Individual health professional factors	Facilitators	CARE SPORTS CONNECTORS| Motivation: professionals' willingness to participate (PHC ans sport professionals' enthusiasm).	Physical activity referral scheme	Qualitative	(60)
2. Individual health professional factors	Facilitators	PHYSICAL ACTIVITY ASSESSMENT:<br>Professional profile: Family physicians with less years of experience (OR: 0.96; CI:0.94 to 1.00; p=0.002), female famility physicians (OR: 2.70; CI: 1.61 to 4.50; p< 0.001), more nurse encounters (OR: 1.26; CI:1.12 to 1.43; p<0.001) were associated with higher PA level assessments provided by the PHC professionals during the past 18 months.<br><br>PHYSICAL ACTIVITY COUSELLING:<br>Professional behaviour: assessment of PA level (OR: 4.32; CI: 2.37 to 7.85; p < 0.01).<br>Professional profile: less FP experience (OR: 0.97; CI: 0.94 to 0.99; p=0.01) and more nurse encounters (OR: 1.22; CI: 1.10 to 1.35; p < 0.01) were associated with higher PA counselling.	Physical activity assessment<br>Physical activity counselling	Quantitative	(61)
2. Individual health professional factors	Facilitators	STAKEHOLDERS| Cognitions / attitudes: Increased understanding of the importance of targeting and supporting physical activity in healthcare	Physical activity prescription	Qualitative	(62)
2. Individual health professional factors	Facilitators	PHYSICIANS| Cognitions / attitudes: Decrease of counselling barrier identification.<br>Knowledge and skills: Confidence; Knowledge.	Physical activity counselling and prescription	Quantitative	(64)
2. Individual health professional factors	Facilitators	PHC HEALTH PROFESSIONALS| Motivation: health professionals were highly motivated to undertake PA promotion in diabetes care and willing to share the responsibilities of promoting PA with colleagues and patients.	Physical activity counselling	Qualitative	(67)

		Knowledge and skills: gathering data from patients through research on perceptions and barriers to PA in order to identify potentially effective PA interventions; extensive training for the team involved in diabetes care.			
2. Individual health professional factors	Facilitators	FAMILY PHYSICIANS| Cognitions / attitudes: family physicians' confidence in their ability to write PA prescriptions (facilitator at the begining, but confidence dissipated with the passing of time - intention-behaviour gap).<br><br>Professional behaviour: participants tried to assess patients' readiness to change, and if they felt their patient was ready to become active, they were more inclined to write a PA prescription.	Physical activity prescription	Qualitative	(68)
2. Individual health professional factors	Facilitators	GENERAL PRACTITIONERS| Cognitions/attitudes: GPs should educate patients with CKP about how to change their lifestyle for the better.	Physical activity counselling	Quantitative	(69)
2. Individual health professional factors	Facilitators	PHYSIOTHERAPISTS| Knowledge and skills: Further training in motivational interviewing and population screening required.	Physical activity counselling and referral	Mixed-methods	(70)
2. Individual health professional factors	Facilitators	NURSES| Knowledge and skills: nurses appreciated the HPEP session because it offered them tools to assist with feeling more in control of trying to encourage patients to consider increasing their exercise	Physical activity counselling	Qualitative	(71)
2. Individual health professional factors	Facilitators	FAMILY PHYSICIANS | Knowledge and skills: clinician 's ability to motivate the patient for successful behaviour change; Confidence and familiarity; professional responsibility to facilitate PA assessment.; Professional training, knowledge and competencies provided clinical knowledge of the benefits associated with PA.; Patient selection; clinicians' prior knowledge, clinicians' perceived confidence, clinicians'personal experience with PA.	Physical activity assessment and counselling	Qualitative	(76)
2. Individual health professional factors	Facilitators	HEALTH PROFESSIONALS:<br>Knowledge and skills: to have a bird 's eye view of the whole neighbourhood; knowledge about the PA offer.<br><br>Cognitions / attitudes: own interest; interest in PA and a belief in PA promotion as a means of stimulating a healthy lifestyle.	Physical activity counselling and referral	Qualitative	(77)
2. Individual health professional factors	Facilitators	PROFESSIONALS:<br>Knowledge and skills: knowledge (i.e. completing the online training programme has improved knowledge of diabetes and the value of PA for glycaemic control); skills (i.e. the training programme has facilitated acquisition of behaviour change skills); intentions (i.e. completing the training programme has increased the likelihood that PA will be targeted in future consultations); and optimism (i.e. practice will improve delivery of the intervention during routine consultations and lead to beneficial changes in patient behaviour); beliefs about capabilities (i.e. practice delivering the intervention to patients will make it easier to use);	Physical activity counselling	Qualitative	(78)
2. Individual health professional factors	Facilitators	PHYSICIANS:<br>Cognitions / attitudes: Respondents reported a greater perception of belief of effectiveness of PA counseling;<br><br>Knowledge and skills: adequate training	Physical activity counselling	Quantitative	(82)
2. Individual health professional factors	Facilitators	GENERAL PRACTITIONERS:<br>Cognitions / attitudes: GPs do the counseling due to the benefits from PA in health.<br><br>Knowledge and skills: Training was intended to improve physician practices.	Physical activity counselling	Qualitative	(84)
2. Individual health professional factors	Facilitators	PHC PHYSICIANS: Sociodemographic characteristics: The prevalence of the discussion about PA with most at-risk patients was higher among PCPs who identified as non- Hispanic white compared to "Other," among PCPs working in the South compared to those in the Northeast, among nurse practitioners compared to internists, and among those in practice > 20 years compared<br>to those in practice 3–5 years and 11–20 years.	Physical activity counselling	Quantitative	(85)
2. Individual health professional factors	Facilitators	PRACTITIONERS:<br>Knowledge and skills: Training, mentoring, appraisals, and on-going CPD were consistently referenced to by practi-tioners as essential operating procedures forimplementation.<br>Professional behaviour: person-centred climate; cognizant practitioners; GP enthusiasm for ERS.	Physical activity referral scheme	Qualitative	(89)
2. Individual health professional factors	Facilitators	PATIENTS:<br>Professional behaviour: Support and accountability provided by the coach was invaluable; Appreciated that clinicians/coaches guided them to be active safely.<br>COACHES AND CLINICIANS:<br>Scope of practice/professional role: felt that their role of encouraging behavior change (coach) and safety monitoring (clinician) aligned well with	Physical activity counselling	Mixed-methods	(90)

		their clinical expertise, and was professionally rewarding.			
2. Individual health professional factors	Facilitators	GPs:<br>Cognitions / attitudes: to be convinced of the interest of prescription (42%).	Physical activity counselling	Quantitative	(91)
2. Individual health professional factors	Facilitators	STAKEHOLDERS:<br>Health profile: Referrers who were known to have an active life-style were perceived to be more likely to refer to the scheme.	Physical activity referral scheme	Qualitative	(92)
2. Individual health professional factors	Facilitators	HCPs AND PATIENTS:<br>Professional behaviour: direct promotion of PA and PARS information by HCPs would foster the functionality of the PARS process and enhance the delivery; use of information sessions, campaigns, and media to promote the programme/raise awareness; Rapport building between HCPs and patients was viewed by participants as pivotal to improving the functionality of PARS; GPs felt that spending more time with patients could help them better promote PA to the patient; Patients emphasised the importance of rapport building between HCPs and patients as this is essential for patient uptake and adherence.<br><br>GPs:<br>Professional behaviour: GPs urged Exercise Physiologists to use forums, such as information sessions to inform the public about distinctions between their roles and other allied health professionals; need to improve the media promotion of Exercise Professional services through multiple channels.<br><br>Knowledge and skills: Participants proposed the inclusion of PA and PARS training in the curriculum of prospective medical graduates - GPs felt that being knowledgeable about interventions that could be useful to their patients and implementing them would be invaluable to their practice; Exercise Professionals argued that including PA and PARS information into the medical curriculum would help GPs to effectively deliver quality PA and PARS care to their patients; Patients corroborated these views.<br><br>EXERCISE PROFESSIONALS:<br>Professional behaviour: Exercise Professionals corroborated the views of the GPs by saying that the dissemination of PARS information could help enlighten the public on the benefits of taking up PA interventionalprogrammes, improve awareness about the roles and services EPs provide and help patientsseek referrals themselves.<br><br>PATIENTS:<br>Professional behaviour: promotional materials, such as pamphlets,available to GPs could help the doctors promote the programme better.	Physical Activity Referral Scheme	Qualitative	(93)
2. Individual health professional factors	Facilitators	HCPs:<br>Professional behaviour: increase confidence when counselling patients.	Physical activity counselling	Qualitative	(94)
2. Individual health professional factors	Facilitators	PATIENTS:<br>Professional behaviour: exercise specialists created a safe and supportive environment, instilling confidence in participants from initial consultation to the end of the programme (qual).	Physical activity referral scheme	Mixed-methods	(56)
2. Individual health professional factors	Facilitators	PATIENTS:<br>Professional behaviour: reinforcement (i.e. feedback and social support from healthcare professionals during consultations is an incentive to taking part); social influences (i.e. practical and emotional support from healthcare professionals at the primary care practice is important for maintaining motivation);	Physical activity counselling	Qualitative	(78)
2. Individual health professional factors	Facilitators	PATIENTS:<br>Professional behaviour: Staff support	Physical activity referral scheme	Qualitative	(83)
2. Individual health professional factors	Facilitators	STAKEHOLDERS:<br>Knowledge and skills: Many participants viewed the availability of people with expert knowledge in SPAP at the primary healthcare centre as important for implementation	Physical activity prescription	Qualitative	(62)
2. Individual health professional factors	Facilitators	PROFESSIONALS:<br>Cognitions/attitudes: a key influence on effective GP referral implementation was the presence of a practice manager or staff member who had an appreciation for the value of PA for patients (qual)	Physical activity referral scheme	Mixed-methods	(56)
2. Individual health professional factors	Facilitators	NURSES:<br>Scope of practice/professional role: nurses should assume leading in PA promotion.	Physical activity prescription	Qualitative	(95)
2. Individual health professional factors	Facilitators	Behaviour: GPs: You must be familiar with these patients and you must be able to reach them with the necessary sensitivity (I-28m)	Physical activity counselling	Qualitative	(96)

2. Individual health professional factors	Facilitators	EXERCISE PRACTITIONER:<br>Knowledge and skills: the training and development from the academic teams (and behaviour change skills training) was really beneficial.	Physical activity referral scheme	Qualitative	(97)
2. Individual health professional factors	Facilitators	PHC PHYSICIANS:<br>Sociodemographic characteristics: Female physicians reported counseling a higher percentage oftheir patients than male physicians (p < 0.001; median for female physicians = 70 (50-80) vs. Median for male physicians = 50 (30-70)).	Physical activity counselling	Quantitative	(98)
2. Individual health professional factors	Facilitators	Knowledge and skills: In the bivariate analysis, there was a greater chance of counseling among those with undergraduate degree, with a graduate degree (vs. no graduate degree) (p<0.001), and with academic experience in PHC (p<0.001); After adjustment for all covariates, the analy-<br>sis showed that who had academic experiences in PHC during initial training remained associated with a higher chance of counseling (OR=2.68; 95%CI: 1.32;5.92) .<br>Professional profile: In the bivariate analysis, there was a greater chance of counseling among those who belonged to the Family Health Support Centers' team (p=0.012) and with a graduate degree in Public Health (p<0.001); After adjustment for all covariates, the analy-<br>sis showed that professionals with a graduate degree in Public Health remained associated with a higher chance of counseling (OR=3.71; 95%CI:1.69;9.37) and who belonged to the Family Health Support Centers' team (OR=4.52; 95%CI: 1.31;28.50) .<br>Behaviour: In the bivariate analysis, there was a greater chance of counseling among those who were physically active (p=0.009); After adjustment for all covariates, the analysis showed that professionals who were physically active remained associated with a higher chance of counseling (OR=1.80; 95%CI: 1.01;3.27).	Physical activity counselling	Quantitative	(100)
3. Patient factors	Barriers	PATIENTS:<br>Preferences: Deslike of being dictated to do something and that, in particular, formal prescribing of PA may not be always be taken positively by some patients.<br>Needs: lack of opportunity to ask questions about what is involved and to meet with people before turning up for the first time to the PA opportunity/program.	Physical activity referral scheme	Qualitative	(49)
3. Patient factors	Barriers	PATIENTS (1/4; qual.):<br>Motivation(?): lack of time to attend consultations	Physical activity prescription	Mixed-methods	(50)
3. Patient factors	Barriers	PATIENTS: Expectations: PAP becomes an eye opener - some participants were not motivated by PAP, and the discovery of a metabolic risk factor made them feel disappointed or depressed (however, continued follow-ups contributed to a sense of support and security and motivated them to continue)	Physical activity prescription	Qualitative	(54)
3. Patient factors	Barriers	Sociodemographic characteristics: patients' with higher annual family income were associated with less PA Counselling (OR: 0.56; CI: 0.32 to 0.97; p = 0.04) by PHC professionals during the last 18 months.	Physical activity counselling	Quantitative	(61)
3. Patient factors	Barriers	PHYSICIANS: Preferences: Patient prefer medication management (sig PTPT change p = .001);	Physical activity counselling and prescription	Quantitative	(64)
3. Patient factors	Barriers	GENERAL PRACTITIONERS: Preferences: patient prefer other management options	Physical activity counselling	Quantitative	(69)
3. Patient factors	Barriers	PHC PROFESSIONALS: Motivation: Patients not interested in exercise; lack of interest.<br>Preferences: Patients prefer medication management.<br>Health status: patient competing illness.	Physical activity counselling and prescription	Quantitative	(72)
3. Patient factors	Barriers	Patients:<br>Awareness/attitudes: memory, attention and decision processes (i.e. it takes time to fully understand the nature of the intervention and its components).	Physical activity counselling	Qualitative	(78)
3. Patient factors	Barriers	PATIENTS:<br>Beliefs and knowledge: misunderstanding of the invitation to participate.<br><br>Health status: Where current health or health behaviours were considered good, the lack of any pressing need meant that many interviewees did not necessarily see value in allotting time to receiving or acting on advice to increase activity levels; Interviewees tended to view their surgery as a place to go only when already sick (health prevention seen as a less legitimate use of resources than for treatment of an existing condition); provision of advice on physical activity was something that would only be welcomed if relevant to an existing health condition.<br><br>Motivation: lack of personal relevance of the trial (self-identified as already sufficiently active or felt that the information they would receive would be of no use to them); For interviewees with a pre-existing health condition necessitating considerable contact with the healthservice, the idea of attending primary care for further testing was a deterrent (no additional time; anxiety about the potential for new tests to reveal yet more health	Physical activity counselling	Qualitative	(80)

		problems); concern that measurement, and participation more generally, may be daunting and potentially embarrassing; feeling of being dictated to or patronised by health professionals giving information that interviewees were already familiar with.			
3. Patient factors	Barriers	Health status: illness and comorbidities (e.g. stroke) made it harder for participants to fully engagewith e-coachER (qual.).<br><br>Adverse events and contingencies: bereavement, injury and other health issues as a barrier to ERS commitment (qual.); participants'working patterns; unexpected life events made it difficult to engage with e-coachER.<br><br>No statistically significant differences between IG (ERS+web-based digital support) and CG (only ERS) regarding ERS attendance (primary analysis/Intention-To-Treat (ITT) analysis complete-case comparisonat 12 months: OR 1.13 (95%CI: 0.72 to 1.79);p=0.58; secondary analysis/ITT imputed comparison at12 months: OR 1.09 (95% CI: 0.70 to 1.71);p=0.70) (quant.).	Physical activity referral scheme	Mixed-methods	(81)
3. Patient factors	Barriers	PHYSICIANS:<br>Motivation: Respondents reported a greater perception of priority to counsel patients about PA in the contemplation and preparation stages, compared with the pre-contemplation and maintenance stages.	Physical activity counselling	Quantitative	(82)
3. Patient factors	Barriers	PATIENTS:<br>Previous experiences: Previous negative experience of PA.<br>Awareness/attitudes: Negative perceptions of PA within personal social context; perceived lack of support.<br>Adverse events and contingencies: complex life circumstances.<br>Health status: poor health, social anxiety.	Physical activity referral scheme	Qualitative	(83)
3. Patient factors	Barriers	GPs:<br>Preferences: Patient preferences.<br>Expectations: Some patients expect GPs to perform an examination, write a prescription for medicine and/or refer to a specialised diagnosing and treatment in the healthcare system, not make a signpost to PA.<br>Compliance/engagement: GPs regarded psychological and social health-issues to be less accepted by society than biological health factors and that stigmatisation associated with non-medical needs of patients may act as a barrier for patients' participation in Social Prescription.	Physical activity counselling and referral	Qualitative	(87)
3. Patient factors	Barriers	HEALTH CARE PRACTITIONERS:<br><br>Compliance/Engagement: Many HCPs hesitated about prescribing exercise, believing patients do not have time or the energy to do it (the burden of patients'daily lives made it difficult for them); Many HCPs pointed out that the very health conditions that brought patients to their clinics were ones that hindered them from exercising.<br><br>Preferences: patients were stuck in a"viciouscycle"of wanting to use pills or ice for aching joints insteadof exercising	Physical activity counselling	Qualitative	(88)
3. Patient factors	Barriers	PATIENTS:<br>Expectations: Felt the program was missing more intense exercise training options relevant for them.	Physical activity counselling	Mixed-methods	(90)
3. Patient factors	Barriers	GPs:<br>Compliance/Engagement: Patients' predicted non-compliance (medium: 2.08; scale 0-4); refusal of the patients (medium: 1.36; scale 0-4).<br>Sociodemographic characteristics: Language barrier (medium: 1.22; scale 0-4).	Physical activity counselling	Quantitative	(91)
3. Patient factors	Barriers	PATIENTS AND REFERRERS:<br>Motivation: Low motivational status of a patient at the time of referral.<br>Beliefs and knowledge: patients have low recognition of scheme value (perceive many other problems to be more prominent in their lives).<br>REFERRERS:<br>Behaviour and feedback: the absence of formal scheme feedback as a key barrier.	Physical activity referral scheme	Qualitative	(92)
3. Patient factors	Barriers	GENERAL PRACTITIONERS | Motivation: Patients are not motivated, GPs said (mean=4,64 - score from 1 to 10) (barrier).	Physical activity prescription	Quantitative	(44)
3. Patient factors	Barriers	GENERAL PRACTITIONERS | Expectations - perception that patient expectation for that consultation is not PA (qual.) (barrier).	Physical activity counselling and referral	Mixed-methods	(47)
3. Patient factors	Barriers	PHP PROFESSIONALS | Awareness/attitudes: PHC professionals think patients may not be aware of the importance of preconception PA and/or they seek other information sources (barrier)	Physical activity counselling	Qualitative	(48)

3. Patient factors	Barriers	PHC PROFESSIONALS | - Compliance/engagement: Patients receptivity; health professional beliefs about patients engagement with the referral (barrier or facilitator); Hps perceptions about the barriers for their patients, such as lack of time, availability, accessibility, and suitability (barrier).<br>- Awareness/attitudes: HPs perceptions that many patients did not think that the PA opportunities available were for them (barrier).<br>- Awareness/attitudes: HPs perceptions that many patients did not think that the PA opportunities available were for them (barrier).	Physical activity referral scheme	Qualitative	(49)
3. Patient factors	Barriers	HEALTH PROFESSIONALS | Previous experiences: reluctant clients (perception that many clients do not want to talk about PA because they had bad experiences of physical education at school, or they are afraid of attending an exercise group).	Physical activity counselling and referral	Mixed-methods	(51)
3. Patient factors	Barriers	PHC PHYSICIANS| Motivation: patients' lack of motivation to exercise<br>Expectations: PCPs perceived patients expect a passive treatment	Physical activity prescription	Qualitative	(55)
3. Patient factors	Barriers	PROFESSIONALS:<br>Health status: patients' concerns regarding potential for exacerbating existing health issues were perceived as barriers for participation in PA sessions (qual)..<br><br>Expectations: and the fear of embarrassment were perceived as barriers for participation in PA sessions (qual).	Physical activity referral scheme	Mixed-methods	(56)
3. Patient factors	Barriers	PHC PHYSICIANS| Motivation: Patients are not interested in improving their PA (32,65%);<br>Expectations: patients expect drug treatments when they visit their GP (15,64%).<br>Behaviour and feedback: too dificult for patients to change their behaviour (27,21%).	Physical activity counselling	Quantitative	(57)
3. Patient factors	Barriers	PHC PROVIDERS| Motivation - patients are unlikely to be motivated to follow advice to be more active (56,5% physicians, 70,1% nurses, 76,9% nurse assistants, 61% dietitians and 53,9% health educators Agree or Strongly agree);<br>Expectations: Patient expect drug treatment when they visit their GP practice (54,7% physicians, 60,1% nurses, 88,4% nurse assistants, 61,3% dietitians and 92,3% health educators Agree or Strongly agree)	Physical activity counselling	Quantitative	(63)
3. Patient factors	Barriers	PHC PROVIDERS | Motivation - Patients not interested in exercise (sig PTPT change p = .001)	Physical activity counselling and prescription	Quantitative	(64)
3. Patient factors	Barriers	PHC HEALTH PROFESSIONALS| Motivation: patients' lack of motivation, laziness, with no self-confidence, not willing to accept PA.	Physical activity counselling	Qualitative	(67)
3. Patient factors	Barriers	FAMILY PHYSICIANS | Compliance/engagement: perception of low compliance by their patients with the PA prescriptions (which would require having to make more significant lifestyle changes), compared with drug prescriptions.	Physical activity prescription	Qualitative	(68)
3. Patient factors	Barriers	3.Patient factors:<br>PATIENTS| Health status: Type 2 Diabetes patients may not feel there is enough in it for them to motivate them to change behaviour, because the 'perceived threat' is not great enough;<br>NURSES| Beliefs and knowledge: Nurses believe that patients may see little 'perceived benefit' in the adoption of exercise, since medication will provide the same benefit	Physical activity counselling	Qualitative	(71)
3. Patient factors	Barriers	HEALTH PROFESSIONALS | Health status: Fewer than 50 % of physicians recommended physical activity to their patients with breast cancer or cervical cancer; only 66,5% of nurses recommended physical activity to their patients with breast or cervical cancer.	Physical activity counselling	Quantitative	(74)
3. Patient factors	Barriers	HEALTHCARE PROFESSIONALS | Expectations: if patients go to the doctors with a problem, they are not interested in discussing physical activity.<br>Beliefs and knowledge: one HCP feel that gaps in patient knowledge pertaining to physical activity can create a disconnect between a HCP and patient.	Physical activity assessment	Qualitative	(79)
3. Patient factors	Barriers	PHC PHYSICIANS | Compliance/engagement: 35.4% of PCPs selected "Patients won't do it"	Physical activity counselling	Quantitative	(85)
3. Patient factors	Barriers	HEALTH PROFESSIONALS:<br>Compliance/engagement: adherence to appointments.<br>Behaviour and feedback: Participant don't attend on time (qual.)	Physical activity prescription	Mixed-methods	(50)

3. Patient factors	Barriers	PATIENTS | Adverse events and contingencies: environmental context and resources (i.e. a period of ill health or adverse health events could create barriers to increasing levels of PA)	Physical activity counselling	Qualitative	(78)
3. Patient factors	Barriers	PATIENTS| Adverse events and contingencies: time constraints (work and/or family commitments).	Physical activity counselling	Qualitative	(80)
3. Patient factors	Barriers	PATIENTS:<br>Compliance/Engagement: ERS difficult to commit to, perhaps more so than other self-selected activities, whereas others expressed a lack of interest in the types of exercise being offered.	Physical activity referral scheme	Mixed-methods	(81)
3. Patient factors	Barriers	PHC PROFESSIONALS| Compliance/engagement: Reluctant to give guidance as it will not translate into behaviour change, in those patients who may not be open to it (barrier).<br><br>Behaviour and feedback: patients did not frequently approach them for preconception PA guidance (barrier).	Physical activity counselling	Qualitative	(48)
3. Patient factors	Barriers	HEALTH PROFESSIONALS:<br>Behaviour and feedback: difficult to bring up PA with the client because clients are usually unwilling to talk about PA.	Physical activity counselling and referral	Mixed-methods	(51)
3. Patient factors	Barriers	PHC PHYSICIANS| Motivation: Patients lack motivations (66.1%).<br>Preferences: Patient prefers drugs (35.8%).	Physical activity counselling	Quantitative	(52)
3. Patient factors	Barriers	NURSES:<br>Awareness: Lack of awareness (and education) on PA benefits in some patients.<br>Knowledge and beliefs: Lack of (awareness and) education on PA benefits in some patients.<br><br>GPs:<br>Knowledge and beliefs: Lack of information in some patients.	Physical activity prescription	Qualitative	(95)
3. Patient factors	Barriers	Health status: GPs: (PA advice) is particularly challenging in case of older people, as more specifics (e.g. previous diseases, compatibility with ongoing therapies) must be taken into consideration and the need for care would be greater.	Physical activity counselling	Qualitative	(96)
3. Patient factors	Barriers	PATIENTS:<br>Awareness/attitudes: "I know what I need to do. It isn't the Dr's responsibility to ensure people exercise. Exercise is a personal choice."	Physical activity prescription	Qualitative	(99)
3. Patient factors	Barriers	QUANTITATIVE PHASE:<br>Motivation: Patients not motivated to take up PARS referral (5% GPs; 55% Eps; 10% total).	Physical activity referral scheme	Mixed-methods	(103)
3. Patient factors	Facilitators	Preferences: Phone calls were appreciated because they provided an opportunity for individual guidance (facilitator).<br><br>Motivation: Participants did not want to break the agreement they had made with the diabetes nurse, they wanted to avoid feelings of embarrassment and guilt; regular telephone follow-ups helped them stick to their plan; Reaching the 'goal' in the activity tracker was satisfying and seen as a reward; improved physical wellbeing was seen as a reward (facilitators).	Physical activity counselling	Qualitative	(43)
3. Patient factors	Facilitators	PATIENTS:<br>Motivation: Perceived HP as a facilitator/role of influence.<br>Preferences: Being formally referred to a PA oppotunity would legitimate patients to go to that PA opportunity.<br>Needs: social support (e.g. "buddy system") to help motivate and support patients towards taking the first step towards the PA opportunity/program.<br><br>PHC PROFESSIONALS:<br>Compliance/engagement: patient's receptivity, perceived by the health professional (barrier or facilitator); health professional beliefs about patients engagement with the referral (barrier or facilitator); perceived self position of influence (facilitator).	Physical activity referral scheme	Qualitative	(49)
3. Patient factors	Facilitators	PATIENTS:	Physical activity	Mixed-methods	(50)

		Preferences: want to keep pedometers	prescription		
3. Patient factors	Facilitators	PATIENTS:<br>Expectations: PAP becomes an eye opener - some participants felt surprised and motivated by PAP (expected only pharmacological treatment).<br>Motivation: Positive effects of PA on health contribute to motivation (monitoring/follow-ups).<br>Beliefs and knowledge: support from a physiotherapist would be more appropriate than support from a nurse.	Physical activity prescription	Qualitative	(54)
3. Patient factors	Facilitators	PATIENTS:<br>Sociodemographic characteristics: There was a significantly higher proportion of those aged 70 or above attending a session com-pared with those that did not (55.2% vs 46.5%, respectively; p= 0.01).<br>Trust: signposting to a range of alternative local physical activity classes were best received when personally recommended by an exercise specialist, as they were seen to provide trusted advice (qual).	Physical activity referral scheme	Mixed-methods	(56)
3. Patient factors	Facilitators	PATIENTS| motivation: the nurses were seen to provide motivation and encouragement when the 'novelty' of the intervention was waning and participants were at risk of lapsing<br><br>NURSES | Motivation: BCT's that were especially evident in our participants' comments were (1) the provision of information, (2) monitoring and feedback and (3) strategies for relapse prevention/overcoming challenges	Physical activity counselling	Mixed-methods	(59)
3. Patient factors	Facilitators	PHYSICAL ACTIVITY ASSESSMENT:<br>Health status: patients' higher physical component summary of quality of life (OR: 1.03; CI:1.00 to 1.05; p=0.03), and higher mental component summary of quality of life (OR: 1.03; CI: 1.00 to 1.05; p=0.04) were associated with higher PA level assessments provided by the PHC professionals during the past 18 months.<br><br>PHYSICAL ACTIVITY COUNSELLING:<br>Health status: Overweight or obese individuals (OR: 3.21; CI: 1.46 to 7.12; p < 0.01), those with type 2 diabetes or pre diabetes (OR: 2.84; CI: 1.51 to 5.37; p < 0.01), and those who had a higher physical component summary of quality of life (OR: 1.06; CI: 1.03 to 1.10; p < 0.01) were more likely to receive PA counselling by PHC professionals during the last 18 months.	Physical activity assessment	Quantitative	(61)
3. Patient factors	Facilitators	PHC PROFESSIONALS: Health status: PHC professionals were consistent about the fact that they advise PA, if if linked to their presenting health problem (p=0.001); All healthcare professionals were likely to provide PA advice 'always' to overweight patients (54–77%) than any other medical conditions.	Physical activity counselling	Quantitative	(63)
3. Patient factors	Facilitators	PATIENTS: Sociodemographic characteristics: Age, gender, marital status, education, employment status were not signifcant predictors.<br>Health status: Depression, fatigue, BMI, chronic disease was not significant predictors.<br>Expectations: Positive outcome expectations was a positive and significant predictor (OR=1.71 (1.09; 2.69). Goal setting, action plan, self-efficacy, negative outcome expectation, social support and quality of life were not significant predictors.<br>Preferences: no significant preferences between face to face interventions, face to face intervention plus telephone calls, and e-mail intervention	Physical activity counselling	Quantitative	(66)
3. Patient factors	Facilitators	Patients:<br>Beliefs and knowledge (i.e. the intervention increased knowledge and awareness of the benefits of PA for management of type 2 diabetes); skills (i.e. the intervention equipped patients with the self-management skills to plan, monitor and overcome barriers to PA); beliefs about capabilities (i.e. increasing the levels of PA was not as difficult as anticipated when using the intervention); beliefs about consequences (i.e. increasing the levels of PA will have important health benefits); optimism (i.e. the intervention will help to increase and maintain the levels of PA); intentions (i.e. the intentions to attempt to increase the levels of PA have become stronger as a result of the intervention); goals (i.e. setting personal goals is an important aspect of the intervention); behavioural regulation (i.e. self-monitoring activity levels have facilitated an increase in PA).	Physical activity counselling	Qualitative	(78)
3. Patient factors	Facilitators	PATIENTS:<br>Motivation: Perceived personal gains (e.g. health status check; receiving novel advice on PA) were more relevant motivators for participation in preventative interventions generally; perceived social benefits (e.g. desire to give back to others or seeking recognition or praise for participation) were more salient influences on the decision to participate in a research trial specifically.	Physical activity counselling	Qualitative	(80)
3. Patient factors	Facilitators	PATIENTS:<br><br>Previous experiences: Previous positive experience of PA.<br><br>Awareness/attitudes: positive perceptions of PA within personal social context; physical and mental health.	Physical activity referral scheme	Qualitative	(83)

		Motivation: motivation to improve health status; Peer support; improved exercise confidence; enjoyment; improvement or maintenance of health; improved fitness			
3. Patient factors	Facilitators	GENERAL PRACTITIONERS:<br>Health status: Presence of NCDs	Physical activity counselling	Qualitative	(84)
3. Patient factors	Facilitators	GPs:<br>Motivation: works best for patients who are confident and skilled enough to find their own way to PAs after the consultation.	Physical activity counselling and referral	Qualitative	(87)
3. Patient factors	Facilitators	HEALTH CARE PROFESSIONALS:<br>Health status: Eight (out of 10) of the providers explained that they discuss PA more with patients who are overweight.	Physical activity counselling	Qualitative	(88)
3. Patient factors	Facilitators	PATIENTS:<br>Motivation: Many perceived functional improvements and a “healthy aging” mindset as a benefit; Some perceived benefits of improved overall health/type 2 diabetes (T2D) care.	Physical activity counselling	Mixed-methods	(90)
3. Patient factors	Facilitators	STAKEHOLDERS:<br>Awareness/attitudes: increased patient awareness leading to self-referrals; patient awareness of the scheme as a facilitator to referral, with an increasing number of patients requesting to be referred.<br>PATIENTS AND REFERRERS:<br>Motivation: High motivational status of a patient at the time of referral.<br>Behaviour and feedback: a patient shared their positive scheme experiences with their referrer was described as a key facilitator for referral (key motivator for making future referrals).	Physical activity referral scheme	Qualitative	(92)
3. Patient factors	Facilitators	PATIENTS:<br>Beliefs and knowledge: information about the services of Exercise Professionals be made readily available in the community, particularly in key healthcare centres(e.g., hospitals)	Physical Activity Referral Scheme	Qualitative	(93)
3. Patient factors	Facilitators	PROFESSIONALS:<br>Motivation: patients' personal motivation to lose weight facilitated participation in a PA session (qual).	Physical activity referral scheme	Mixed-methods	(56)
3. Patient factors	Facilitators	STAKEHOLDERS| Preferences: it was frequently assumed that patients would find more mainstream spaces of exercise (such as gyms) intimidating compared with ERS PA resources.	Physical activity referral scheme	Qualitative	(65)
3. Patient factors	Facilitators	FAMILY PHYSICIANS| Compliance/engagement: patients follow up in which physicians realized they follow the prescription (patients' drive).<br>Health status: patients at risk of developing certain chronic diseases.	Physical activity prescription	Qualitative	(68)
3. Patient factors	Facilitators	GENERAL PRACTITIONERS| Motivation: It is the patient’s own responsibility to continue doing their exercise programme	Physical activity counselling	Quantitative	(69)
3. Patient factors	Facilitators	HEALTH PROFESSIONALS<br>Health status: more than 95% of physicians recommended that their patients with high BMI, dyslipidemia,hypertension, and type 2 diabetes practice physical activity; More than 95% of nurses recommended that their patients with high BMI, dyslipidemia, hypertension, and type 2 diabetes actively participate in physical activity.	Physical activity counselling	Quantitative	(74)
3. Patient factors	Facilitators	GPs:<br>Compliance/Engagement: Being convinced that their recommendations had an impact was significantly associated with the use of PA prescription as a non-medicinal therapeutic choice ($p=0.002$) (81% vs. 30%).	Physical activity counselling	Quantitative	(91)
3. Patient factors	Facilitators	GPs:<br>Sociodemographic characteristics: About one-third of the GPs (28) reported that they would advise elderly patients more often with regard to physical exercise; for one-third (26), this is occasionally the case.<br>Behaviour: We have had older patients who are dissatisfied with their physical constitution and address us for this reason.<br>Health status: the group of chronically ill people who need special attention; A larger fraction of the interviewed GPs (50) estimated that in most cases the subject of exercise and sports is initially addressed by themselves and counselling ensues on this basis - this applies in particular if exercise prescribed as a longer term measure—promises a stabilisation or improvement of the patient’s health condition (e.g. obesity, diabetes).<br>Motivation: Others in turn search for activities that keep them occupied and bring them in contact with other people.<br>Trust: older patients are unsure to what extent sports programmes could exacerbate pre-existing maladies - need of this patient clientele “to get a kind of sense of security”, and the GPs’ advice is taken seriously by most patients and submitted recommendations are accepted; addressing the already mentioned “need for safety” by giving perfectly appropriate recommendations and advising their patients not to take any risks when	Physical activity counselling	Qualitative	(96)

		engaging in physical activities - that is why the elderly choose to rely on professional guidance.<br>Motivation: of central importance not just to appeal to the patient to do exercises, but rather encourage an “intrinsic motivation” in him or her, which can be “internalised quickly in the sense of a natural daily or weekly rhythm”.			
3. Patient factors	Facilitators	Sociodemographic charactaristics: In the multivariate analysis, sedentary behaviour counseling remained positively associated with the sociodemographic characteristics: female sex (PR: 1.77; 95% CI: 1.10–2.83) and age group ≥60 yrs (PR: 1.84; 95% CI: 1.14–2.98).<br><br>Health status: The probability of receiving counseling was higher among users who BMI ≥ 30.0 kg/m2 (PR: 2.60; 95% CI: 1.31–5.17) and who consumed ≥3 continuous medications (PR: 2.21; 95% CI: 1.06–4.59).<br><br>Behaviour: The probability of receiving counseling was higher among those sitting ≥1.8 h/day. A higher magnitude of association was found among those who remained seated ≥6.2 h/day (PR: 3.44; 95% CI: 1.88–6.31).	Physical activity (sedentary behaviour) counselling	Quantitative	(101)
3. Patient factors	Facilitators	QUALITATIVE PHASE:<br>Motivation: Participants’ perceptions about PARS showed that motivating patients regarding PARS is essential for effective uptake of the programme.	Physical activity referral scheme	Mixed-methods	(103)
4. Professional interactions	Barriers	GPs:<br>Team processes: Practice nurse makes referrals (n=3; quant.).<br>Team communication: no mechanism of feedback from the referral scheeme about patient progress (qual.) (barrier).<br>Referral processes: Unclear referral pathway (n=1; quant.); Paper referral form asks unnecessary questions (n=1; quant.) (barriers).	Physical activity counselling and referral	Mixed-methods	(47)
4. Professional interactions	Barriers	HEALTH PROFESSIONALS:<br>Team communication: physicians and PA counsellors“do not speak the same language”	Physical activity counselling and referral	Mixed-methods	(51)
4. Professional interactions	Barriers	PHC PHYSICIANS: Team processes: Physicians who worked with 15 or more nurses per PHC provide less verbal behavioral counseling to patients with chronic disease (OR, 0.27(CI 0.08–0.91), p<0.04) and systematically track/follow up less patients with chronic disease (OR, 0.1; (CI 0.03–0.31); p<0.001).	Physical activity counselling	Quantitative	(57)
4. Professional interactions	Barriers	STAKEHOLDERS:<br>Team processes: need for cooperation with a physiotherapist, i.e. a health professional perceived to be an expert on physical activity; physiotherapist was mentioned by several participants as particularly suitable for the role of local SPAP coordinator, as an expert on physical activity for people with complex health disorders; limited collaboration between healthcare and physical activity organisers; Lack of local written routines at healthcare centres was an important barrier to prescribing SPAP.	Physical activity prescription	Qualitative	(62)
4. Professional interactions	Barriers	STAKEHOLDERS:<br>Networks: lack of reciprocity, or co-operative bonds of association in ERS at the strategic level; difficulty of developing mutuality/reciprocity at steering group meetings (in which all ERS stakeholders, including third parties, were invited to network fora, indicing lack of familiarity) consisting of a large and frequently changing set of stakeholders was prohibitive to the development of the programme; county-level ‘Public Health’ and county’s sports partnership perceived at district level as ‘weak leadership’ instead reflected a deliberate strategy designed to enable district managers to set their own targets and to deliver locally tailored programmes.<br><br>Team communication: lack of communication between the county and district/local ERS levels, and between exercise professionals and primary care health professionals; due to perceptions of disinterest amongst primary health care professionals, a number of Exercise Professionals confirmed they had not been asked for, and so did not offer, feedback on the performance of individual patients<br><br>STAKEHOLDERS| Mutual trust: Exercise professionals perceived do not be trusted by primary care health professionals (primary health care professionals made no comment upon the professional competence of Exercise Professionals).	Physical activity referral scheme	Qualitative	(65)
4. Professional interactions	Barriers	PHC PROFESSIONALS: Networks: professionals did not have a clear shared mission on the CSC role and the connection between the sectors.	Physical activity counselling and referral	Qualitative	(77)
4. Professional interactions	Barriers	PATIENTS: Referral processes: problems with ERS oversubscription; delays in the referral process or staffing level; opportunistic sampling via general practice was slow (qual.).	Physical activity referral scheme	Mixed-methods	(81)
4. Professional interactions	Barriers	PATIENTS:<br>Referral process: duration between referral and the initial counseling session (OR= 0.95 (0.93; 0.98))	Physical activity counselling	Quantitative	(66)

4. Professional interactions	Barriers	HEALTH PROFESSIONALS:<br>Referral processes: poor PA referrals	Physical activity counselling	Qualitative	(67)
4. Professional interactions	Barriers	HEALTH PROFESSIONALS:<br>Networks: other PA stakeholders (sectors) were not supporting the Ministry of Health in PA promotion or implementing opportunities effectively.	Physical activity counselling	Qualitative	(67)
4. Professional interactions	Barriers	PHC PROFESSIONALS:<br>Team processes: The professionals expected to have a more structural form of collaboration, with regular contact with other professionals, a clear referral scheme, and more involvement of other organizations; Welfare professionals and sport professionals commented on their possible position as rivals.	Physical activity counselling and referral	Qualitative	(77)
4. Professional interactions	Barriers	GPs:<br>Mutual trust: GPs concerned regarding PA standards and confidentiality in the third sector, in comparison with the professional health sector (sports clubs<br>unsolicited access to patient charts); volunteers' competences (third/sports sector), including lack of knowledge and experience of working with people with diseases, may be potential barriers in times where evidence-based professionalism has been an increasingly important aim in the public sector; High staff turnover in the third sector organisations was mentioned by all GPs as a potential barrier for collaboration, because it will weaken the development of trusting relationships.	Physical activity counselling and referral	Qualitative	(87)
4. Professional interactions	Barriers	CARE SPORTS CONNECTORS| Mutual trust: PHC professionals' apprehensiveness about sport clubs (reason for the lack of referral, perceived by the care sport connectors).	Physical activity referral scheme	Qualitative	(60)
4. Professional interactions	Barriers	NURSES:<br>Team processes: the leading role in PA prescription is not clear.<br>Networks: Lack of knowledge about external PA resources within external policies relationships in PAP; in collaborative exercise prescription, they demand/lack of help from specialists in patients with co-morbidities or specific pathologies.<br><br>GPs:<br>Networks: they demand/lack of help by other specialists for individualized and effective PA treatments of exercise prescription; They need/lack of help by staff inside and outside of PHC settings, within a collaborative PA promotion; lack of/They demand help from specialist physicians,<br>mainly in some specific patients, within a collaborative exercise prescription.<br>Team processes: Doubts about how PA promotion and prescription is considered by the rest of physicians; clarify the tasks.	Physical activity prescription	Qualitative	(95)
4. Professional interactions	Barriers	GPs:<br>Networks: absence of alliances ("Where are higher-level alliances?"); "I perceive no comprehensive network, in which we as GPs are able to participate. As in so many other fields, we are lone fighters. It deprives us of many opportunities".	Physical activity counselling	Qualitative	(96)
4. Professional interactions	Barriers	EXERCISE PRACTITIONERS:<br>Networks: co-production process - One key challenge included different views on who could attend the referral scheme and who was responsible for these patients.<br>Referral processes: having the patient book their own induction is too daunting [which would be the case for self-referral] - I have seen clients standing outside not knowing whether to go and book one or how to do<br>it - This isn't an issue if they are referred by a healthcare professional and an induction is booked for them.<br>Team processes: the more staff we involved, the more difficult it became to organise the intervention.<br><br>PUBLIC HEALTH COMMISSIONER AND GP:<br>Referral processes: "In terms of challenges, I guess I had a very particular point of view; that the referral should be very easy for GPs to refer, or even make it so that GPs were not necessary to refer. However, the council seemed more averse to this, potentially representative of different cultures and the more risk averse view of those who wear a council hat. This issue of risk aversion versus innovation played out quite regularly, from my perspective. (...) we are often constrained by preconceived rules, which are simply constructs, rather than<br>immutable laws (i.e. GP refers patient, patient goes to referral, etc). We need to ask more 'Do we really have to stick to X rules?'"	Physical activity referral scheme	Qualitative	(97)
4. Professional interactions	Barriers	QUANTITATIVE PHASE:<br>Networks: Lack of national collective goal or coordination process on referral pathways (20% GPs; 41% Eps; 10% total).<br><br>QUALITATIVE PHASE:<br>Networks: (Inter)organisational Mechanism - Major (inter)organisational obstacle to the success of the PARS programme included poor EP accessibility, knowledge gaps, complicated administrative processes and time constraints.	Physical activity referral scheme	Mixed-methods	(103)

		Team processes: Relational coordination - Both participant groups indicated that an improved interprofessional relationship could be beneficial in the coordination of optimum care for patients. They stressed the need for feedback and information sharing to foster trust and improved functionality ofthe PARS programme. GPs indicated that they don't receive feedback from EPs on the patients they refer to the EPs, while the EPs claimed that the GPs were not proactive enough in following up with the feedback from the PARS consultation. Instead, the feedback is often filed away by administrative staff, and this might prevent information from getting across to the doctors.<br>Referral processes: Exercise professionals expressed concerns about the delayed referral of patients to PARS and how this could make it difficult for the clients to achieve their health goals.			
4. Professional interactions	Facilitators	DIETITIANS: Team processes: Dynamics of the Family Health Teams (e.g. Family Health Team focus on preventing chronic diseases; being seen as health promoters) (facilitators)	Physical activity counselling	Qualitative	(46)
4. Professional interactions	Facilitators	GPs: Referral processes: Clearer marketing information about content (46%; quant.); Clearer marketing information about eligibility (45%; quant.); If the scheme was more physical activity focused, rather than only based at fitness centres (30%; quant.); Referral system needs to be quick and easy, shouldn't need patient data like height and weight or pulse (n=1; quant.); Self-referral (n=6; quant) (facilitators).<br><br>Team communication: Better feedback about patient progress (27%; quant.); Most effective ways to communicate with GPs - Hard copy leaflets (61%; quant.); Hard copy posters (54%; quant.); Leaflets for referrers with exercise referral scheme information on (41%; quant.); Short instructional video (18%; quant.); Exercise referral scheme representatives to present details at GP forums (29%; quant.); An additional event for GPs (4%; quant.); Online circulation of initiative information (61%; quant.); Receptionists and other non-clinical staff involvement (GPs not the only people in contact with patients) (n=1; quant) (facilitators).	Physical activity counselling and referral	Mixed-methods	(47)
4. Professional interactions	Facilitators	PATIENTS:<br>Referral processes: patients 'meet and greet' with organisers and members of PA resources in the area	Physical activity referral scheme	Qualitative	(49)
4. Professional interactions	Facilitators	HEALTH PROFESSIONALS:<br>Team processes: cooperation between physical activity counsellors and health care professionals is one of the most important issues in a successful PA referral process; appreciated the opportunity to ask for help and discuss the problems in their work with other PA counsellors	Physical activity counselling and referral	Mixed-methods	(51)
4. Professional interactions	Facilitators	PROFESSIONALS:<br>Team communication: good communication across all levels of programme delivery (qual).	Physical activity referral scheme	Mixed-methods	(56)
4. Professional interactions	Facilitators	STAKEHOLDERS:<br>System/organizational characteristics: primary healthcare centres that had clear written routines for cooperation and referral pathways were reported by the participants to be more successful in the implementation of SPAP.	Physical activity prescription	Qualitative	(62)
4. Professional interactions	Facilitators	STAKEHOLDERS: Team processes: importance of reciprocity in local face-to-face, long-term and embodied partnership work, including good communication, supportive attitudes and consistency at the interpersonal level, between exercise professionals and primary care health professionals	Physical activity referral scheme	Qualitative	(65)
4. Professional interactions	Facilitators	HEALTH PROFESSIONALS: Team processes: Family physician doctors suggested a team approach to promote PA in diabetes care.	Physical activity counselling	Qualitative	(67)
4. Professional interactions	Facilitators	PHC PROFESSIONALS:<br>Team processes: Internal collaboration in PA counselling	Physical activity counselling	Quantitative	(75)
4. Professional interactions	Facilitators	PHC PROFESSIONALS:<br>Networks: Primary care professionals in particular stressed the importance of a connection between sectors, because more collaboration may result in increased referral of patients; helping sport professionals in developing new PA activities; Professionals in three partnerships mentioned a shared mission, which facilitated the collaboration; be a driving force and/or initiator in the connection between both sectors.<br>Team processes: Providing an insight into the current PA opportunities.	Physical activity counselling and referral	Qualitative	(77)
4. Professional interactions	Facilitators	PATIENTS: Referral processes: Engaging the ERS team to help gave a significant improvement in recruitment rates (qual.)	Physical activity referral scheme	Mixed-methods	(81)
4. Professional interactions	Facilitators	GPs:<br><br>Networks: Cross-sector collaboration between general practice and voluntary associations; potential facilitator of successful Social Prescribing that the PAs take place; equal partnership and mutual respect between parties were essential for the collaboration.<br><br>Team processes: outside the healthcare setting (incorporate PA as a natural and permanent part of daily life, it should be experienced as a pleasant way to spend one's leisure time rather than a temporary medical treatment); GPs can recruit new members to the local sports club;	Physical activity counselling and referral	Qualitative	(87)

4. Professional interactions	Facilitators	PRACTITIONERS:<br>Team communicaton: GeneralPractitioners (GPs)/ referrers through phone calls, email,documentation, meetings, role modelling, and vicarious learning influences their motivation and capability ("you had a set structure and you had people to consult with like to bounce ideas off it was so much more motivating like when you were in a team to like enhance your practice); integration would influence the motivation of practitioners when GPs understand, value, and are interested in ERSs and behaviour change, and when there is a perceived shared effort across professions; feedback loops.	Physical activity referral scheme	Qualitative	(89)
4. Professional interactions	Facilitators	PATIENTS/COACHES/CLINICIANS:<br>Team processes: recommendation to simplify staff documentation.	Physical activity counselling	Mixed-methods	(90)
4. Professional interactions	Facilitators	GPs:<br>Team processes: collaboration between the physician and medical-sport educators, physiotherapists and sports doctors (89%).	Physical activity counselling	Quantitative	(91)
4. Professional interactions	Facilitators	REFERRERS:<br>Team communication: introduction of a formal feedback mechanism was requested as a facilitator for future referrals.	Physical activity referral scheme	Qualitative	(92)
4. Professional interactions	Facilitators	EXERCISE PROFESSIONALS:<br>Team processes: GPs' awareness of the roles and services exercise professionals render is critical to the programme's success.<br><br>HCPs AND PATIENTS:<br>Team processes: EPs substantiated the views of the GPs by noting that collaboration between a GPand an EP who share a common goal would enhance quality PA and PARS care delivery; Patients recommended that frontline HCPs, such as GPs, should be constantly reminded of available EP services and provided with printed information to be disseminated to their patients.<br><br>Team communication: Clear and effective communication among HCPs and between HCPs and patients were viewed as vital to achieving success in delivering quality care in PA and PARS services - GPs emphasised the need to maintain a good information exchange channel to help them keep up to date with the care of the patients they referred, EPs substantiated the views of the GPs and suggested an overview of current communication pathways to include useful tools, such as templates to help guide the information exchange between them and frontline HCPs (e.g., GPs).	Physical Activity Referral Scheme	Qualitative	(93)
4. Professional interactions	Facilitators	CARE SPORTS CONNECTORS| Team processes: inviting physiotherapists to attend a sport lesson	Physical activity referral scheme	Qualitative	(60)
4. Professional interactions	Facilitators	STAKEHOLDERS| Networks: county's sports partnership considered themselves a network facilitator – e.g. through the creation of county network group fora.	Physical activity referral scheme	Qualitative	(65)
4. Professional interactions	Facilitators	PHC PROFESSIONALS:<br>Networks: alternative connecting solutions that rely on an intermediary or resource including practice champions, link workers within practices, and community hubs (facilitator)	Physical activity referral scheme	Qualitative	(49)
4. Professional interactions	Facilitators	HEALTH PROFESSIONALS:<br>Networks: need to organise common meetings with various actors; opening and enlarging the network that develops health promotion and PA counselling (e.g. involving sports clubs, municipal council, childhood education, schools, third sector organisations and the local media)	Physical activity counselling and referral	Mixed-methods	(51)
4. Professional interactions	Facilitators	CARE SPORT CONNECTORS:<br>Team communication: showing the result after referral and having regular contact with primary care professionals about their role increased their involvement.	Physical activity referral scheme	Qualitative	(60)
4. Professional interactions	Facilitators	GPs:<br>Networks: Sports-Health network (86%).	Physical activity counselling	Quantitative	(91)
4. Professional interactions	Facilitators	HEALTH PROFESSIONALS:<br>Networks: Involve all stakeholders (qual.)	Physical activity prescription	Mixed-methods	(50)
4. Professional interactions	Facilitators	CARE SPORT CONNECTORS:<br>Networks: making use of ambassadors as a way to get in contact with primary care professionals; shared goal and a concrete plan of action were general facilitating factors in establishing a connection between the primary care and the PA sector.	Physical activity referral scheme	Qualitative	(60)
4. Professional interactions	Facilitators	PHC PROFESSIONALS:<br>Networks: representatives from municipal PA services or community centre.	Physical activity counselling	Quantitative	(75)
4. Professional interactions	Facilitators	NURSES:<br>Networks: Use of network team and public resources outside the health-care center: Sport medicine physicians and inside with psychologists and physiotherapists; Help from specialists in patients with co-morbidities or specific pathologies within a PAP networking modus operandi; Send patients to public sport centers using economic offers through health-care prescription; Mandatory relationship nurse - exercise professional of public sport	Physical activity prescription	Qualitative	(95)

		centers; Improving relationship between Town Hall and healthcare services.<br>Team processes: To work with health professionals inside the system, like physiotherapist and specialist physicians; Mandatory relationship between a nurse and family physician; Use of first consultation by nurses.<br><br>GPs:<br>Networks: Network team and public system relationship (Community and Town hall); Help from specialists in patients with co-morbidities or specific pathologies; Improve relationships inside and outside of the health-care centers; They demand help by other specialists to individualized effective PA treatments; Improving relationship with public institutions; Use of external resources: sports centers, town hall, and sport medicine schools.<br>Team processes: Use of nurses as a physician assistant to PA promotion and prescription; Use nurse consultations as the first resource to the PA promotion and prescription in the current System.			
4. Professional interactions	Facilitators	GPs<br>Networks: especially physicians who had been trained in sports medicine, were active in healthcare networks and, referred their patients to selected health and sports centres and physiotherapists, as needed and if patients were interested; transparency and contact persons are needed, particularly when it is about physical fitness in old age; the communities and the federal states have a duty to do more - they have to build a network-like, interdisciplinary structure in which many actors come together; the communities and the federal states have a duty to do more - they have to build a network-like, interdisciplinary structure in which many actors come together.<br>Team processes: possibilities of delegation to further qualified office staff members able to support, inform, and motivate patients, for which the general practitioner is not necessarily needed.	Physical activity counselling	Qualitative	(96)
4. Professional interactions	Facilitators	PATIENTS:<br>Networks: process of iterative co-production (i.e. to work with local stakeholders to develop an intervention that addressed the shortcomings of the original intervention by drawing on scientific evidence, practical ‘craft’ knowledge, and service user expertise) - "the most important thing on is to listen to the people that actually use the service and those on the ground who deliver the service – see what they want, see if they actually want the change. Make sure they are included and feel listened to, as we did.".<br><br>EXERCISE PRACTITIONER:<br>Networks: "(the co-production process) allowed us to get a variety of information - For example, I was able to provide operational information, like the realistic delivery perspectives, the service users helped us understand what they needed, the commissioners funding, and the academics for the science."; you need other organisations to help create multidisciplinary processes that work together to provide better options for patients.<br>Referral processes: patients referred by a healthcare professional and an induction is booked for them.<br>Team processes: "have a set of core practitioners run the intervention. This worked much more effectively." (than have too much staff involved).<br><br>FITNESS CENTRE AREA MANAGER:<br>Networks: Co-production process - Obvious strengths were getting the perspectives from everyone involved. For me, the key stakeholders were the service users, and getting their feedback direct to the practitioners was invaluable. (...) It’s a better model from<br>a business and individual health perspective; Giving the stakeholders authentic buy-in is critical; involving multiple stakeholders from day one and making sure they are working towards the same goal - I believe that’s what made this project successful, within funding constraints.<br><br>PUBLIC HEALTH COMMISSIONER AND GP:<br>Networks: co-production - a good process, which was collaborative and included many different points of view from clinical and commissioning perspectives to those representing the delivery of the scheme.<br><br>ACADEMIC:<br>Networks: co-production process brought people together who wouldn’t otherwise have been having those conversations - it provided time or headspace to come away and ask ‘how can we work together in this, and is there a better way we could do this?’ The co-production meetings enabled participants to voice their concerns and ideas within a psychologically safe space, practitioners to be in a room with commissioners they rarely have contact with, and collaborative group decisions to be made that reflected everyone’s needs; importance of working with those on the ground who will be delivering and receiving services; co-production process - A key strength of the iterative, phased development approach was that it allowed the intervention to be embedded in practice during the research process itself. We were able to tackle teething problems as and when they arose, and we developed a culture of mutual learning and respect – if something wasn’t working, we had an open conversation and worked out a better way to do things; stakeholder involvement lies at the heart of successful intervention design; value of working with, and listening to the voices of those who will be delivering and benefiting from the intervention.	Physical activity referral scheme	Qualitative	(97)

4. Professional interactions	Facilitators	Networks: In the bivariate analysis, there was a greater chance of counseling among those who participated in matrix meetings on physical activity (with a Physical Education Professional integrated in these teams/in the PHC) ($p<0.001$); After adjustment for all covariates, the analysis showed that professionals who participated in physical activity matrix meetings remained associated with a higher chance of counseling (OR=1.91; 95%CI: 1.08;3.44).	Physical activity counselling	Quantitative	(100)
4. Professional interactions	Facilitators	QUANTITATIVE PHASE:<br>Team communication: Ongoing interactions between GPs and EPs (65% GPs; 93% Eps; 23% total).	Physical activity referral scheme	Mixed-methods	(103)
5. Incentives and resources	Barriers	GPs:<br>Assistance tools and materials: lack of instructional materials (37%).<br>Human resources: lack of exercise experts for conducting counseling (30%).<br>Physical activity opportunities: lack of neighborhood features for physical activity practice (21%) (barriers)	Physical activity prescription	Quantitative	(44)
5. Incentives and resources	Barriers	DIETITIANS: Human resources: not having a PA expert in the health team (barrier).	Physical activity counselling	Qualitative	(46)
5. Incentives and resources	Barriers	GPs: Continuing education system: lack of education provision both with regards to PA counselling and the local exercise referral scheme (qual.) (barrier)	Physical activity counselling and referral	Mixed-methods	(47)
5. Incentives and resources	Barriers	PHC PROFESSIONALS:<br>Assistance tools and materials: Lack of up-to-date resources with information about PA opportunities (barrier).	Physical activity referral scheme	Qualitative	(49)
5. Incentives and resources	Barriers	HEALTH PROFESSIONALS:<br>Health facilities: physical challenges (e.g no dedicated room, not welcoming space, no proper aiting areas).<br>Human resources: lack of staff/staff turnover (qual.).<br>Assistance tools and materials: clinic require more supportive physical activity tools (qual.)	Physical activity prescription	Mixed-methods	(50)
5. Incentives and resources	Barriers	PGC PHYSICIANS: Limited resources (53.2%).	Physical activity counselling	Quantitative	(52)
5. Incentives and resources	Barriers	PATIENTS: Financial (des)incentives: expensive memberships in PA facilities	Physical activity prescription	Qualitative	(54)
5. Incentives and resources	Barriers	PROFESSIONALS:<br>Financial (des)incentives: concerns about engagement from GP surgeries beyond project funding (qual).	Physical activity referral scheme	Mixed-methods	(56)
5. Incentives and resources	Barriers	PHC PHYSICIANS:<br>Assistance tools and materials: lack of adequate referral services for PA (54,42%); lack of effective tools and information to give to patients (26,53%).<br>Physical activity opportunities: lack of effective treatment options (12,24%).<br>Financial (des)incentives: Inadequate reimbursement (3,4%).	Physical activity counselling	Quantitative	(57)
5. Incentives and resources	Barriers	STAKEHOLDERS:<br>Assistance tools and materials: not having a computer and printer close by; Contact lists of activity organisers were available at some of the primary healthcare centres, but were not updated and therefore not helpful.<br>Financial (des)incentives: reimbursement based on the number of written SPAP prescriptions (barriers and facilitator)	Physical activity prescription	Qualitative	(62)
5. Incentives and resources	Barriers	PHC PROVIDERS:<br>Assistance tools and materials: educational material for patients are insufficient (53,1% physicians, 51,4% nurses, 57,7% nurse assistants, 54,8% dietitians and 69,3% health educators Agree or Strongly agree).<br><br>Financial (des)incentives: would be more likely to promote physical education if there was a financial incentive (27,5% physicians, 28,1% nurses, 50% nurse assistants, 16,1% dietitians and 38,5% health educators Agree or Strongly agree).<br><br>Continuing education system: there is a lack of available education for health professional regarding PA promotion (55% physicians, 56,2% nurses, 65,4% nurse assistants, 74,2% dietitians and 76,9% health educators Agree or Strongly agree).	Physical activity counselling	Quantitative	(63)
5. Incentives and resources	Barriers	PHYSICIANS:<br>Assistance tools and materials: Lack of guidance/resources in exercise for those with chronic disease (sig PTPT change $p = .001$)	Physical activity counselling and prescription	Quantitative	(64)
5. Incentives and resources	Barriers	STAKEHOLDERS: Financial (des)incentives: Lack of funding to deal with ERS participants in some circumstances (e.g. cardiac rehab); strategic managers claimed they lacked the means and the budget to regulate programmes, and local delivery mechanisms remained unchallenged.	Physical activity referral scheme	Qualitative	(65)

5. Incentives and resources	Barriers	HEALTH PROFESSIONALS:<br>Health facilities: lack of facilities and overall limited space available for patient instruction.<br><br>Assistance tools and materials: lack of educational materials.<br><br>Human resources: inadequate manpower.<br><br>Physical activity opportunities: lack of PA facilities particularly safe walking areas; potential PA facilities within the community, in schools, are underutilized by the public<br><br>Information system: PA is not integrated in the electronic primary health care information system limiting operationalization of PA services.	Physical activity counselling	Qualitative	(67)
5. Incentives and resources	Barriers	FAMILY PHYSICIANS:<br>Assistance tools and materials: lack of visible prescription pads in every consultation room;.<br><br>Information system: use of two systems to make prescriptions and then take the time to enter the information in the patients' dossier.	Physical activity prescription	Qualitative	(68)
5. Incentives and resources	Barriers	PHYSIOTHERAPISTS: Financial (des)incentives: private physiotherapists reported that may be difficult to recruit fee paying patients	Physical activity counselling and referral	Mixed-methods	(70)
5. Incentives and resources	Barriers	PHC PROFESSIONALS: lack of resources.	Physical activity counselling and prescription	Quantitative	(72)
5. Incentives and resources	Barriers	PHC PROFESSIONALS:<br>Assistance tools and materials: provided with supportive material for PA counselling, but its use was not explored	Physical activity counselling	Quantitative	(75)
5. Incentives and resources	Barriers	HEALTH PROFESSIONALS:<br>Financial (des)incentives: time and money; the lack of remuneration in the current health insurance system for preventive work; discontinuation of funding was mentioned as a barrier.<br>Human resources: adequate sport instructors	Physical activity counselling and referral	Qualitative	(77)
5. Incentives and resources	Barriers	PATIENTS:<br>Financial (des)incentives: gym costs	Physical activity referral scheme	Mixed-methods	(81)
5. Incentives and resources	Barriers	PHYSICIANS:<br>Assistance tools and materials: Regardless of patients' stage of change, respondents reported lowest agreeance with having adequate resources provide PA counselling. Respondents reported a greater perception of adequate time during the contemplation, preparation and action stages of the TTM	Physical activity counselling	Quantitative	(82)
5. Incentives and resources	Barriers	GPs:<br>Financial (des)icentives: GPs predicted a risk of increasing social inequality through Social Prescribing, since some patients could not afford contingent to the PA, sports equipment and/or sportswear.	Physical activity counselling and referral	Qualitative	(87)
5. Incentives and resources	Barriers	GPs:<br>Physical activity opportunities: lack of adapted structure (medium: 2.47; scale 0-4).<br>Team support/supervision: lack of support (medium: 1.75; scale 0-4).<br>Continuing education system: Lack of training and knowledge (medium: 1.74; scale 0-4).<br>Financial (des)incentives: No dedicated pricing (1.34; sclae 0-4)	Physical activity counselling	Quantitative	(91)
5. Incentives and resources	Barriers	REFERRERS:<br>Information system: paper-based referral forms, referrers with accounts of data entry errors, duplication of information and an overall time intensive referral activity.	Physical activity referral scheme	Qualitative	(92)
5. Incentives and resources	Barriers	HCPs AND PATIENTS:<br>Financial(des)incentives: Participants reported cost as a barrier to HCPs coordinating PARS care for clients and patients' uptake of PA and PARS initiatives; GPs urged the government to subsidise PA and PARS intervention cost for patients, particularly the elderly; Exercise Professionals suggested the delivery of affordable care by specialists; Patients advocated for cost subsidies to help patients afford the preventative benefits of the programme, rather than paying a huge cost to seek an overdue solution; increase chronic disease management sessions rebate (GPs - from 5 to 10 sessions; Exercise professionals - no limit, to allow specialist enough sessions for behavioural change; patients - extra free sessions to helpmaximise	Physical Activity Referral Scheme	Qualitative	(93)

		the gains of PA and PARS interventions).			
5. Incentives and resources	Barriers	HCPs:<br>Assistance tools and materials: A bug-fixing process was critical for improving the validity and reliability of the application, as well as fixing apps' language issues.	Physical activity counselling	Qualitative	(94)
5. Incentives and resources	Barriers	GENERAL PRACTITIONERS<br>Financial (des)incentives: Patients do not have the material means, GPs said (mean=4,1 - score from 1 to 10) (barrier).	Physical activity prescription	Quantitative	(44)
5. Incentives and resources	Barriers	CARE SPORT CONNECTORS<br>Physical activity opportunities: lack of suitable sport activities for the target group (e.g. too vigorous activities)	Physical activity referral scheme	Qualitative	(60)
5. Incentives and resources	Barriers	PHYSICIANS: Financial (des)incentives: Regardless of patients' stage of change, respondents reported lowest agreeance with feeling adequately reimbursed for PA counselling.	Physical activity counselling	Quantitative	(82)
5. Incentives and resources	Barriers	PATIENTS (1/4; qual.):<br>Trial (dis)incentives: Long and exhausting measurement tools; accelerometers cause discomfort and/or skin irritation; too much instructions.	Physical activity prescription	Mixed-methods	(50)
5. Incentives and resources	Barriers	PHC PROFESSIONALS:<br>Financial (des)incentives: Competing variables (financial, etc).	Physical activity counselling and prescription	Quantitative	(72)
5. Incentives and resources	Barriers	PHC PROFESSIONALS:<br>Physical activity opportunities: Referral 11.4% selected "Referral services aren't available"	Physical activity counselling	Quantitative	(85)
5. Incentives and resources	Barriers	PHC PROFESSIONALS:<br>Financial (des)incentives: 12.1% selected "Insurance doesn't cover it,"	Physical activity counselling	Quantitative	(85)
5. Incentives and resources	Barriers	NURSES:<br>Continuing education system: Lack/Need of training courses' facilities offered by the manager staff and the system (Kind of training courses: motivational interview, assess PA and fitness patterns, time management.).<br>Information system: issues with / Need to change the informatic tool to assess PA/sedentary behavior and fitness as a vital sign to patient.<br>Assistance tools and materials: Lack of material to assess PA patterns and fitness.<br>Physical activity opportunities: Lack of individualized treatments and follow-up to change dangerous lifestyles behaviors in patients.<br>Health facilities: Lack of space resources/need to reorganize the space<br>Financial desincentives: Professional, academic, and economic incentives are not always according to the professional category.<br><br>GPs:<br>Continuing education system: Mandatory PAP training courses and must have priority to the training courses by different methods (online, face-to-face, etc.; Kind of training courses: Motivational interview,<br>time management)<br>Information system: Inefficient tool and no relationship with informatic staff to assess PA patterns and fitness.<br>Health facilities: lack of space resources/Space should be reorganized.<br>Assistance tools and materials: Lack of material to assess PA patterns and fitness.	Physical activity prescription	Qualitative	(95)
5. Incentives and resources	Barriers	GPs:<br>Physical activity opportunities: "Where are the systematic support programmes to which I can refer my patients?".<br>Assistance tools and materials: "Where can I get an overview and information?".<br>Financial desincentives: GPs' unsatisfactory remuneration situation.<br>Lack of team support: GPs, left to their own devices, they would not have a sufficient overview of local sports offers and could not refer their patients to them quickly and free of complications.	Physical activity counselling	Qualitative	(96)
5. Incentives and resources	Barriers	EXERCISE PRACTITIONER:<br>Trial desincentives: "The academics got carried away with the number of measures (i.e. questionnaires) that they wanted patients to complete during consultations. We [exercise referral practitioners] felt they were very similar and repetitive. At first, the data collection was really labour intensive. We had to tell the academic team there were too many things to do at the first consultation".	Physical activity referral scheme	Qualitative	(97)
5. Incentives and resources	Barriers	QUANTITATIVE PHASE:<br>PA opportunities: Scarcity of referral pathways (50% GPs; 38% Eps; 14% total).<br>(Cost and lack) of finantial incentives: Lack of financial incentive (34% GPs; 31% Eps; 11% total).<br>Assistance tools and materials: Lack of reference materials (14% GPs; 27% EPs; 7% total).	Physical activity referral scheme	Mixed-methods	(103)

5. Incentives and resources	Facilitators	Continuing education system: two 1-hour education sessions on exercise prescription based on the ACSM guidelines - significant increase in the rate of exercise prescription with a medium effect size ($p <0.001$; Phi =0.379) post-education<br>Assistance tools and materials: Dissemination of a practitioner toolbox, activated within the electronic medical record (EMR) - significant increase in the rate of exercise prescription with a medium effect size ($p <0.001$; Phi =0.379) post-education	Physical activity prescription	Quantitative	(42)
5. Incentives and resources	Facilitators	GPs: Financial (des)incentives: Reimbursement of adapted PA prescription (mean=4,61 - score from 1 to 10) (facilitator).<br>Assistance tools and materials: Information about where to refer patients (mean=6,7 - score from 1 to 10) (facilitator); Support information for patients, GPs said (mean=4,87 - score from 1 to 10) (facilitator).	Physical activity prescription	Quantitative	(44)
5. Incentives and resources	Facilitators	DIETITIANS: Information system: Dynamics of the Famlily Health Teams (e.g. having access to patients'interdisciplinary health care charts helps in tailoring counselling) (facilitator)	Physical activity counselling	Qualitative	(46)
5. Incentives and resources	Facilitators	GPs: Information system: Electronic referral system (68%; quant.) (facilitator); e-referral system (qual.).	Physical activity counselling and referral	Mixed-methods	(47)
5. Incentives and resources	Facilitators	HEALTH PROFESSIONALS:<br>Information system: Include PA in the Health information system (qual.); Whatsapp Communications may be useful for future PA interventions.	Physical activity prescription	Mixed-methods	(50)
5. Incentives and resources	Facilitators	HEALTH PROFESSIONALS:<br>Assistance tools and materials: need to develop new tools for PA counselling (e.g. virtual/technological tools).	Physical activity counselling and referral	Mixed-methods	(51)
5. Incentives and resources	Facilitators	PATIENTS: Financial (des)incentives: economic subsidies to reduce the cost of joining an exercise facility; trial period before membership	Physical activity prescription	Qualitative	(54)
5. Incentives and resources	Facilitators	PATIENTS:<br>Financial (des)incentives: subsidised rates would be welcomed (qual)	Physical activity referral scheme	Mixed-methods	(56)
5. Incentives and resources	Facilitators	CARE SPORT CONNECTORS: Information system: opting for na easily accessible form of collaboration, for example an online referral form.	Physical activity referral scheme	Qualitative	(60)
5. Incentives and resources	Facilitators	STAKEHOLDERS:<br>Financial (des)incentives: reimbursement based on the number of written SPAP prescriptions (barriers and facilitator)<br><br>Team support/supervision: Having an easily available central supporting function would facilitate implementation; Central support for organising cooperation with activity organisers outside healthcare.<br><br>Continuing education system: Providing inspiration and coaching, distributing written information and organising educational activities	Physical activity prescription	Qualitative	(62)
5. Incentives and resources	Facilitators	PHYSICIANS: Continuing education system: EIMC workshop (A full-day educational workshop increased confidence by 40%,decreased the impact of barriers by 20%, and increased thepercentage of PCPs who prescribe exercise from 20% to 74%3 months later.)	Physical activity counselling and prescription	Quantitative	(64)
5. Incentives and resources	Facilitators	HEALTH PROFESSIONALS:<br>Assistance tools and materials: Availability of necessary resources: "community mapping" for healthworkers to be aware of PA facilities within the geographical catchment areas of primary health care centres, in order to facilitate PA referrals when advised.<br>Non-financial (des)incentives: utilize health groups to promote PA.	Physical activity counselling	Qualitative	(67)
5. Incentives and resources	Facilitators	FAMILY PHYSICIANS:<br>Assistance tools and materials: having prescription pads everywhere would serve as a reminder for them to prescribe physical activity as well as for their patients to ask about PA prescriptions.<br><br>Information system: electronic version or template of the physical activity prescription pads to be included within the electronic systems to improve accessibility.	Physical activity prescription	Qualitative	(68)
5. Incentives and resources	Facilitators	PHYSIOTHERAPISTS:<br>Human resources: Additional physiotherapy and administrative staff required to support pathway.<br>Assistance tools and materials: Additional patient resources required to support pathway.	Physical activity counselling and referral	Mixed-methods	(70)
5. Incentives and resources	Facilitators	PHC PROFESSIONALS:<br><br>Continuing education system: training in knowledge and skills (combined with provision of standard prescription pads) improved overall frequency of	Physical activity prescription	Quantitative	(73)

		prescribing (from the previous level of 34.6% to 65.0% - X^2 = 8.365,P= 0.004).<br>Assistance tools and materials: Assistance tools and materials: provision of standard prescription pads (in combination with training in knowledgs and skills) improved overall frequency of prescribing (from the previous level of 34.6% to 65.0% - X^2 = 8.365,P= 0.004)<br><br>"intervention (which included training in knowledge and skills - see knowledge and skills section - combined with the provision of standard exercise prescription forms) improved overall frequency of prescribing (from the previous level of 34.6% to 65.0% - X^2 = 8.365,P= 0.004), and also improved the use of some prescription elements (frequency, intensity, and timing) from pre-intervention to post-intervention (p<0.05). No significant differences in exercise prescribing frequency in the post-intervention group were noted when correlated with age, BMI, or chronic illness burden. Documentation of specific exercise type deteriorated and there was no improvement in the use of progression in exercise prescription from pre-intervention to post-intervention."			
5. Incentives and resources	Facilitators	PHC PROFESSIONALS:<br>Continuing education system: 2-hour training session held by the researchers at the beginning of the study; weekly training on PA or PA counselling at their health centre during the development work<br><br>Information system: changing the way to enter information on PA counselling to the electronic patient record system by modifying the record template and user authorisations.	Physical activity counselling	Quantitative	(75)
5. Incentives and resources	Facilitators	PHC PROFESSIONALS:<br>Assistance tools and materials: HCPs indicated that standardizing documentation of patient physical activity levels would improve their current practices (e.g., allow for follow up on patients' progress; HCPs want prompts and messages that cater to a broad range of patients; HCPs want a decision algorithm, similar to algorithms used for diabetes.<br><br>PATIENTS:<br>Human resources: patients acknowledged that HCPs are not experts in physical activity and other resources are needed to supplement what the HCPs provid	Physical activity assessment	Qualitative	(79)
5. Incentives and resources	Facilitators	STAKEHOLDERS:<br>Assistance tools and materials: Stakeholders felt that the EAM is carefree, easy to wear, and it has some attractive features (i.e. competition, Smartcoach).<br><br>Financial (des)incentives: They felt that counseling is already a part of practice behavior and the other aspects of the study can be implemented into the clinic if there was a funding source, like grants or insurance, to supply patients with monitors.	Physical activity counselling	Mixed-methods	(86)
5. Incentives and resources	Facilitators	GPs:<br>Assistance tools and materials: The GPs requested a systematic tool to support the signposting-process and to provide patient-tailored and specific PA counselling (e.g. digital platform, a website or an application for mobile devices), to create an overview of PAs in the patient's local community, readily accessible to both the GPs and patients; a shared website with updated information about PAs in the local community would be a potential facilitator for collaboration.<br><br>Financial (des)incentives: GPs requested a financial contribution from the municipality to the PAs of the financially weakest patients.	Physical activity counselling and referral	Qualitative	(87)
5. Incentives and resources	Facilitators	PATIENTS/COACHES/CLINICIANS:<br>Assistance tools and materials: recommendation to provide a video training to patients on uploading PA data.	Physical activity counselling	Mixed-methods	(90)
5. Incentives and resources	Facilitators	GPs:<br>Information system: Realization of patient files (84%).<br>Assistance tools and materials: website/help software (52%).<br>Continuing education system: Training organization (70%).<br>Financial (des)incentives: compensation/reimbursement for PA registration fees for patients (71%); compensation for GPs (37%).	Physical activity counselling	Quantitative	(91)
5. Incentives and resources	Facilitators	STAKEHOLDERS:<br>Financial (des)incentives: the subsidised cost of the scheme with patients seeking an opportunity to access leisure facilities cheaper than traditional memberships.<br>REFERRERS:<br>Information system: need for an automated system which would reduce both the time and effort required when making a referral.	Physical activity referral scheme	Qualitative	(92)
5. Incentives and resources	Facilitators	HCPs AND PATIENTS:	Physical Activity	Qualitative	(93)

		Financial (des)incentives: consensus among respondents on the need to use incentives as a strategy to facilitate HCPs' provision of quality care in PA and PARS to enhance uptake and adherence to intervention goals by patients; GPs argued for increases in payment as an incentive for coordinating PARS; Exercise Professionals and patients supported this notion and emphasised the importance of holistic approach to healthcare delivery (incentivising GPs; manage all of patients' care and to coordinate it all and to look at a person's overall health file rather than just the acute things).<br>Continuing education system: GPs proposed a general orientation on the services provided by Exercise Professionals; Exercise Profesionals substantiated the views of the GPs by suggesting that PA and PARS education for frontline HCPs, such as GPs, be incentivised to make it worthwhile for the gatekeepers; Patients suggested that GPs may not be fully aware of the promotional incentives provided by the government - they proposed more educational/awareness programmes to help GPs promote the initiative effectively.	Referral Scheme		
5. Incentives and resources	Facilitators	HCPs:<br>Information system: Integrating the application (app) into the electronic medical record system would facilitate its use; PA counselling should be an optional module of the electronic medical record system.<br>Team support/supervision: more feasible if technical support were provided.	Physical activity counselling	Qualitative	(94)
5. Incentives and resources	Facilitators	STAKEHOLDERS| Patient safety systems: importance of careful supervision of the prescribed exercise was considered a key faced – need for 'safety' and 'security' amongst 'patients' during the exercise treatment.	Physical activity referral scheme	Qualitative	(65)
5. Incentives and resources	Facilitators	PHC PROFESSIONALS | Physical activity opportunities: Identifying the need for PA activities for residents is therefore an important prerequisite.	Physical activity counselling and referral	Qualitative	(77)
5. Incentives and resources	Facilitators	PATIENTS:<br>Trial (des)incentives: Measurement forming part of trial protocols was generally seen as useful by interviewees who considered themselves in good health, and so expected positive feedback.	Physical activity counselling	Qualitative	(80)
5. Incentives and resources	Facilitators	NURSES:<br>Information system: Modify PAP vital sign tool, improving relationships with informatic staff through a face-to-face each week to know the exact problems.<br>Health facilities: new space resources solutions - Space resource management in each health-care center, use of external spaces or manage the actual ones.<br>Financial incentives: Send patients to public sport centers using economic offers through health-care prescription.<br>Continuing education system: Modify the actual training course rules, offering more facilities to the professionals; Training courses' offer in coaching, leading, and time management.<br>Trial incentives: Need of a pilot study to establish PAP in PHC settings.<br>Assistance tools and materials: Buy materials (Weight machine, handgrip, and different materials to measure fitness level of patients).<br>Human resources: Increase human resources to improve quality of treatments.<br><br>GPs:<br>Continuing education system: To transfer similar strategies used with smoking, obesity, treatments in PHC settings, i.e. they received the training courses and then they trained the rest of PHC staff, in the same way as the PHC system idiosyncrasy; Training courses' offer for everybody and with different modalities; Mandatory and priority to the training courses by different methods (online, face-to-face, etc.).<br>Information system: The PAP vital sign app should be improved and optimized for health professionals.<br>Health facilities: new space resources solutions - Space resource management in each health-care center, use of external spaces or manage the actual ones.<br>Assistance tools and materials: Improve material conditions.<br>Finantial incentives: Improve economic conditions to the adhered professionals to PAP.	Physical activity prescription	Qualitative	(95)
5. Incentives and resources	Facilitators	GPs<br>Assistance tools and materials: Common was the support of the interviewees' recommendations with aids such as brochures and information leaflets (sports for senior citizens, less often community-based opportunities) or reference to health pages on the internet.<br>Physical activity opportunities: importance of a realistic applicability of the recommendations - the physical exercise options must therefore be perceived as "low threshold" as possible; appropriate structures and programmes; health insurance companies should make their insured elderly members more aware of preventive services - this would be a contribution to relieve the physicians from having to search and find suitable offers.<br>Continuing education system: "a broad range of attractive further qualification formats for physicians and medical office staff" (I-58f).	Physical activity counselling	Qualitative	(96)

5. Incentives and resources	Facilitators	PATIENTS:<br>Physical activity opportunities: "We [service users in the group] had formed a tight social group during the referral scheme. I really don't know if others built social groups like we did – and this would have a very different impact on their experience. For me, it was the friendships that we made that was key.".<br><br>EXERCISE PRACTITIONER:<br>Trial incentives: more streamlined approach for research questionnaires, with only the most important information for the research included.<br>Physical activity opportunities: you need to think about the bigger picture, in our case, it wasn't just increasing exercise, it was about developing a community group that can support themselves socially.<br><br>FITNESS CENTRE AREA MANAGER:<br>Finantial incentives: sustainability is also really important - "it is the long-term affordability that can determine long-term engagement. As the scheme is heavily subsidised, it's trying to develop opportunities for long-term access. As we amended the membership packages (...), we came up with packages that we believed were affordable and met certain population needs, e.g. those that had dependents to look after and could only attend during certain hours. We later found that clients did seem to be transitioning well from the ERS to the longer-term membership – but more is needed to be done here."<br><br>ACADEMIC:<br>Team support/supervision: My role became one of facilitator, providing a structure around which the practitioner team could come up with logistical solutions to enhance delivery. I learned that sometimes the support needed from others isn't academic expertise, but a facilitator to bring out the craft expertise in others.	Physical activity referral scheme	Qualitative	(97)
5. Incentives and resources	Facilitators	PATIENTS:<br>PA opportunities: "liked the [walk in the park program suggested by their PCP] and hopes to try it out."	Physical activity prescription	Qualitative	(99)
5. Incentives and resources	Facilitators	PHC PHYSICIANS:<br>PA opportunities: community program availability (21%).<br>Continuing education system: educational opportunities for health care professionals (20%).<br>Human resources: qualified exercise professional availability (11%).<br>Financial incentives: reduce patient financial barriers (19%).<br>Assistance tools and materials: tools/resources for health care professionals to use (12%); tools/resources for patients (10%).<br><br>PHC NURSES:<br>PA opportunities: community program availability (29%).<br>Continuing education system: educational opportunities for health care professionals (25%).<br>Human resources: qualified exercise professional availability (12%).<br>Financial incentives: reduce patient financial barriers (31%).<br>Assistance tools and materials: tools/resources for health care professionals to use (12%); tools/resources for patients (10%).	Physical activity counselling	Quantitative	(102)
5. Incentives and resources	Facilitators	QUANTITATIVE PHASE:<br>Non-financial incentives: Improved visibility of EPs (72% GPs; 84% Eps; 23% total).<br>Continuing education system: Education about referral pathways (37% GPs; 66% Eps; 15% total); An overview of available referral pathways (43% GPs; 48% EPs; 13% total).<br>Assistance tools and materials: Simplify PARS documentation process (documentation should be optimised for disease management) (20% GPs; 28% EPs; 7% total).<br>Financial incentives: Financial incentives or subsidies for patients (8% GPs; 33% EPs; 6% total).<br><br>QUALITATIVE PHASE:<br>Continuing education system: Participants' perceptions about PARS showed that providing adequate knowledge regarding PARS is essential for effective uptake of the programme.	Physical activity referral scheme	Mixed-methods	(103)
6. Capacity for organizational change	Barriers	GPs:<br>Priority of necessary change: PA promotion is not a strategic priority (qual.) (barrier)	Physical activity counselling and referral	Mixed-methods	(47)

6. Capacity for organizational change	Barriers	PHC PROFESSIONALS:<br>Priority of necessary change: Preventative care is not a priority in primary care (barrier).	Physical activity counselling	Qualitative	(48)
6. Capacity for organizational change	Barriers	STAKEHOLDERS: Capable leadership: The participants claimed that primary healthcare management did not have the time or capability to provide central supporting function.<br><br>PHC PROFESSIONALS:<br>Capable leadership: None of the interviewed primary healthcare professionals perceived that management expressed the importance of providing health promotion counselling, nor did central management explicitly show that they prioritised health promotion; There was a discrepancy between the management stating that there were policy documents available on disease preventive methods, and most of the interviewed primary healthcare professionals being unaware of the content in those documents, and in some cases of their very existence	Physical activity prescription	Qualitative	(62)
6. Capacity for organizational change	Barriers	STAKEHOLDERS: Capable leadership: lack of clarity regarding aims and purpose of the program, lack of common language, shared agendas and collaboration; lack of clarity about roles and responsibilities coming from the strategic level.<br>Organizational regulations, rules, policies: policy centralization (county-level Public Health shift from NHS control to local authority control) caused a shift from a more fluid relationship and less prescriptive relationship into a more prescriptive one, which lead to an apparent resistance to change at several levels of ERS provision.	Physical activity referral scheme	Qualitative	(65)
6. Capacity for organizational change	Barriers	HEALTH PROFESSIONALS: Organizational regulations, rules, policies: Lack or poorly communicated physical activity guidelines and physical activity standards from the central (ministerial) level to the clinical (practical) levels.	Physical activity counselling	Qualitative	(67)
6. Capacity for organizational change	Barriers	STAKEHOLDERS: Organizational regulations, rules, policies: treatment protocol - the accepted treatment direction for those newly diagnosed shows a belief in medicalisation amongst practitioners	Physical activity counselling	Qualitative	(71)
6. Capacity for organizational change	Barriers	PRACTITIONERS:<br>Planning: lack of organisational planning created difficulties when trying to implement.	Physical activity referral scheme	Qualitative	(89)
6. Capacity for organizational change	Barriers	HEALTH PROFESSIONALS:<br>System/organizational functioning: diabetes clinics restricted to morning shifts.	Physical activity counselling	Qualitative	(67)
6. Capacity for organizational change	Barriers	PATIENTS | System/organizational functioning: some interviewees did in fact attempt to take part in the trial, but were prevented from doing so as they could not reach their GP surgery by telephone or book a Health Check appointment during the recruitment period; dissatisfaction with appointment scheduling systems and reception staff of the GP surgeries.	Physical activity counselling	Qualitative	(80)
6. Capacity for organizational change	Barriers	GPs:<br>System/organizational functioning: "Practically, I stand all alone with this. Where are the helping hands?".	Physical activity counselling	Qualitative	(96)
6. Capacity for organizational change	Facilitators	HEALTH PROFESSIONALS:<br>Capable leadership: knowledge sharing - 1.the organization/management should provide presentations about the role of physical activity counselling and referral in health promotion and how it should be done.	Physical activity counselling and referral	Mixed-methods	(51)
6. Capacity for organizational change	Facilitators	PROFESSIONALS:<br>Capable leadership: a surgery's decision to adopt the CLICK into Activity programme was associated witha range of engagement activities with practice staff arranged in advance of programme sign-up and delivery (e.g. importance of the presence of a GP staff member visibly recommending and championing the programme in advance of programme adoption) (qual)	Physical activity referral scheme	Mixed-methods	(56)
6. Capacity for organizational change	Facilitators	STAKEHOLDERS:<br>Capable leadership: Keeping the organisation updated on changes and news; Importance of all health professionals sharing responsibility to advocate health promotion; local coordinator was the champion for SPAP, providing reminders, advice and support to the others, thus pushing the development forward; Many of the informants emphasised the importance of the management's endorsement and attitude for the implementation of SPAP.<br><br>Priority of necessary change: Giving genuine priority to SPAP would generate resources because something else would lose priority.	Physical activity prescription	Qualitative	(62)
6. Capacity for organizational change	Facilitators	STAKEHOLDERS:<br>Monitoring and feedback: Importance of feedback about service quality; face-to-face interaction was considered crucial to ensure effective feedback provision.	Physical activity referral scheme	Qualitative	(65)
6. Capacity for organizational change	Facilitators	PHC PROFESSIONALS:<br>Capable leadership: the member of management was regularly present in the working group	Physical activity counselling	Quantitative	(75)

6. Capacity for organizational change	Facilitators	PRACTITIONERS:<br>Capable leadership: supportive leadership (provision of supportive leadership activities is suggested to influence motivation through identity, intentions, and the emotions of practitioners; supportive leadership through role modelling, clear behaviour change expectations, and the allocation of resources to improve the practice of behaviour change was important).	Physical activity referral scheme	Qualitative	(89)
6. Capacity for organizational change	Facilitators	COORDINATOR:<br>Capable leadership: the onus for enhancing referrer awareness should be placed on national bodies as opposed to a responsibility for local teams.	Physical activity referral scheme	Qualitative	(92)
6. Capacity for organizational change	Facilitators	HCPs AND PATIENTS:<br>Capable leadership: nomination of a specialist HCP whose primary duty will be to coordinate PARS for patients - All respondents (GPs, patients, exercise professionals) nominated a nurse as the best suited HCP for that role.	Physical Activity Referral Scheme	Qualitative	(93)
6. Capacity for organizational change	Facilitators	PHC PROFESSIONALS:<br>Priority of necessary change: The referral of primary care patients and residents towards local PA facilities was also highly prioritized.	Physical activity counselling and referral	Qualitative	(77)
6. Capacity for organizational change	Facilitators	STAKEHOLDERS:<br>System/organizational functioning: several of the participants pointed out that written local routines facilitated the raising of issues of health promoting physical activity with patients and that a written local routine for SPAP was of the utmost importance in the guidance and structure of local work.	Physical activity prescription	Qualitative	(62)
6. Capacity for organizational change	Facilitators	NURSES:<br>System/organizational functioning: To maintain Spanish PHC philosophy - no specify professional task and multi-training among nurses; To decrease the ratio patients attended by<br>nurses daily or increase nurse staff in order to increase PAP consultation time.<br><br>GPs:<br>System/organizational functioning: To maintain Spanish PHC philosophy - no specify professional task and multi-training among physicians; To decrease the ratio patients attended by physicians daily.	Physical activity prescription	Qualitative	(95)
6. Capacity for organizational change	Facilitators	ACADEMIC:<br>Capable leadership: someone needs to lead to make things happen.	Physical activity referral scheme	Qualitative	(97)
7. Social, political and legal factors	Barriers	STAKEHOLDERS: Economic constraints on the health care budget: extra money is needed to establish networks, central supporting structures and cooperation with activity organisers.	Physical activity prescription	Qualitative	(62)
7. Social, political and legal factors	Barriers	GPs:<br>Economic constraints on the health care budget: inadequate supports in French Guiana (medium: 1.8; scale 0-4)	Physical activity counselling	Quantitative	(91)
7. Social, political and legal factors	Barriers	GENERAL PRACTITIONERS | Geographic accessibility: perception that patients are unable to access facilities (n=1; quant.)(barrier)	Physical activity counselling and referral	Mixed-methods	(47)
7. Social, political and legal factors	Barriers	SCHEME DELIVERERS AND PATIENTS:<br>Geographic accessibility: Location, of the referrer and scheme delivery, was identified as a barrier to referral, with key factors concerning area deprivation and scheme accessibility; Poor transport options and greater travel distances to scheme facilities; Requiring multiple bus journeys, uncertainty of travel timetables and disruption to routes were cited as barriers.<br><br>Neighborhood socioeconomic profile: Engaging GP practices within areas of deprivation in referring patients was recognised as a key challenge for scheme deliverers (persistent a lack of referrer engagement despite an increasing number of services available within pockets of deprivation).	Physical activity referral scheme	Qualitative	(92)
7. Social, political and legal factors	Barriers	PATIENTS:<br>Geographic accessibility: difficulties for some participants in accessing the facilities or services being offered by the ERS - geographical location, transport issues (qual.).	Physical activity referral scheme	Mixed-methods	(81)
7. Social, political and legal factors	Barriers	PROFESSIONALS:<br>Public safety: in rural settings evenning sessions were not very participated due to the participants' perception of neighborhood unsafety (qual).	Physical activity referral scheme	Mixed-methods	(56)
7. Social, political and legal factors	Barriers	EXERCISE PRACTITIONER:<br>Health care budget: The way the public health funding is all separated is difficult - Some of our initial ideas in the development meetings were not possible because of politics and funding.<br><br>FITNESS CENTRE AREA MANAGER:	Physical activity referral scheme	Qualitative	(97)

		Funding policies: As always, the key challenge is the funding. Having the funding available to do what's best for those that need support. Trying to deliver an intervention with limited funding, to provide the necessary options and intensity patients need, was an ongoing challenge.			
7. Social, political and legal factors	Barriers	QUANTITATIVE PHASE:<br>Lack of funder policies: Physical activity support services are highly undervalued (40% GPs; 67% Eps; 18% total) - small government funding.<br><br>QUALITATIVE PHASE:<br>Lack of funder policies: limited government funding support, with increased burden of cost (extra sessions) and poor continuity of care for patients.	Physical activity referral scheme	Mixed-methods	(103)
7. Social, political and legal factors	Facilitators	Legislation: The legislative framework was associated with an increase in GP using written physical activity prescriptions (+11.7%, p = 0.0002) (facilitator).	Physical activity prescription	Quantitative	(44)
7. Social, political and legal factors	Facilitators	HEALTH PROFESSIONALS:<br>Funder policies: A specific budget needs to be allocated to maintain PA services in diabetes care (qual).	Physical activity prescription	Mixed-methods	(50)
7. Social, political and legal factors	Facilitators	PHC PROFESSIONALS:<br>Influencial people: Municipal policy was perceived by the professionals as a facilitator when the policy was supportive of organizations participating in the connection.	Physical activity counselling and referral	Qualitative	(77)
7. Social, political and legal factors	Facilitators	GPs:<br>Influencial people: Media communication campaign (78%).	Physical activity counselling	Quantitative	(91)
7. Social, political and legal factors	Facilitators	HEALTH PROFESSIONALS:<br>Geographic accessibility: need for easily accessible PA opportunities.	Physical activity counselling and referral	Mixed-methods	(51)
7. Social, political and legal factors	Facilitators	NURSES:<br>Influencial people: Use of mass media to increase the awareness of PA benefits and PAP at health-care settings.<br>Health care budget: Increase economic resources (to buy materials and human resources).<br><br>GPs:<br>Influential people: with regard to PA advisement policies, use of population awareness to implant PA promotion in health-care centers.<br>Health care budget: Increase economic resources (to buy materials and human resources).	Physical activity prescription	Qualitative	(95)
7. Social, political and legal factors	Facilitators	GPs:<br>Funder policies: opportunities in the federal government's recent ability to prescribe licensed digital health apps (DiGA).	Physical activity counselling	Qualitative	(96)
7. Social, political and legal factors	Facilitators	EXERCISE PRACTITIONER:<br>Funder policies: With the correct funding (and procedures) in place, it can be done.<br><br>ACADEMIC:<br>Funder policies: funding opportunities for 'implementation science', broadened beyond the traditional linear research model.	Physical activity referral scheme	Qualitative	(97)
7. Social, political and legal factors	Facilitators	PHC PHYSICIANS:<br>Influential people: greater public promotion (18%).<br>Health care budget: improvements in billing structure (21%).<br><br>PHC NURSES:<br>Influential people: greater public promotion (12%).<br>Health care budget: improvements in billing structure (4%).	Physical activity counselling	Quantitative	(102)
