# Supplementary material for: Implementation determinants of physical activity interventions in primary health care settings using the TICD framework: a systematic review
Source: BMC Health Serv Res. 2023 Oct 11;23:1082. doi: 10.1186/s12913-023-09881-y (PMC10568782; doi:10.1186/s12913-023-09881-y)
Supplement: Supplementary file 6 — Additional file 6. Certainty assessment of the systematic review (SURE checklist). [file 12913_2023_9881_MOESM6_ESM.pdf]

S6 File – Certainty assessment of the systematic review (SURE checklist)

A - Identification, selection and appraisal of studies

A1) Were selection criteria reported?

Yes

A2) Was the search comprehensive?

Partially (language bias was not avoided, only included published studies)

A3) Is the review up-to-date?

Yes

A4) Was biased selection of articles avoided?

Yes

A5) Were appropriate criteria used to assess the risk of bias?

Yes (JBI checklists and MMAT tool)

A6) Overall identification, selection and appraisal of studies

Reliable

B - Analysis of the findings

B1) Were characteristics and results of included studies reliably reported?

Yes

B2) Were methods used to analyse the findings reported?

Yes

B3) Was the extent of heterogeneity described?

Yes

B4) Were the findings combined (or not combined) appropriately?

Yes

B5) Were factors that could explain heterogeneity explored?

Yes

B6) Overall analysis of findings

Reliable

C - Overall assessment of the reliability of the review

C1) Other considerations

No other quality issues identified

C2) Overall reliability of the review

Reliable: This is a good quality systematic review with only minor limitations
